# Toxins from scratch? Diverse, multimodal gene origins in the predatory robber fly *Dasypogon diadema* indicate a dynamic venom evolution in dipteran insects

**DOI:** 10.1093/gigascience/giz081

**Published:** 2019-07-09

**Authors:** Stephan Holger Drukewitz, Lukas Bokelmann, Eivind A B Undheim, Björn M von Reumont

**Affiliations:** 1 Institute for Biology, University of Leipzig, Talstrasse 33, 04103 Leipzig, Germany; 2 Project group Bioresources, Animal Venomics, Fraunhofer Institute for Molecular Biology and Applied Ecology, Winchesterstrasse 2, 35392 Gießen, Germany; 3 Evolutionary Genetics Department, Max Planck Institute for Evolutionary Anthropology, Deutscher Platz 6, D-04103 Leipzig, Germany; 4 Centre for Advanced Imaging, The University of Queensland, St. Lucia, QLD 4072, Australia; 5 Centre for Ecology and Evolutionary Synthesis, Department of Biosciences, University of Oslo, PO Box 1066 Blindern, 0316 Oslo, Norway; 6 LOEWE Centre for Translational Biodiversity Genomics (LOEWE-TBG), Senckenberganlage 25, 60325 Frankfurt, Germany; 7 Institute for Insect Biotechnology, Justus Liebig University, Heinrich Buff Ring 58, 35394 Gießen, Germany

**Keywords:** toxin gene evolution, orphan genes, venom evolution, single gene co-option, gene duplication, comparative venom-genomics

## Abstract

**Background:**

Venoms and the toxins they contain represent molecular adaptations that have evolved on numerous occasions throughout the animal kingdom. However, the processes that shape venom protein evolution are poorly understood because of the scarcity of whole-genome data available for comparative analyses of venomous species.

**Results:**

We performed a broad comparative toxicogenomic analysis to gain insight into the genomic mechanisms of venom evolution in robber flies (Asilidae). We first sequenced a high-quality draft genome of the hymenopteran hunting robber fly *Dasypogon diadema*, analysed its venom by a combined proteotranscriptomic approach, and compared our results with recently described robber fly venoms to assess the general composition and major components of asilid venom. We then applied a comparative genomics approach, based on 1 additional asilid genome, 10 high-quality dipteran genomes, and 2 lepidopteran outgroup genomes, to reveal the evolutionary mechanisms and origins of identified venom proteins in robber flies.

**Conclusions:**

While homologues were identified for 15 of 30 predominant venom protein in the non-asilid genomes, the remaining 15 highly expressed venom proteins appear to be unique to robber flies. Our results reveal that the venom of *D. diadema* likely evolves in a multimodal fashion comprising (i) neofunctionalization after gene duplication, (ii) expression-dependent co-option of proteins, and (iii) asilid lineage-specific orphan genes with enigmatic origin. The role of such orphan genes is currently being disputed in evolutionary genomics but has not been discussed in the context of toxin evolution. Our results display an unexpected dynamic venom evolution in asilid insects, which contrasts the findings of the only other insect toxicogenomic evolutionary analysis, in parasitoid wasps (Hymenoptera), where toxin evolution is dominated by single gene co-option. These findings underpin the significance of further genomic studies to cover more neglected lineages of venomous taxa and to understand the importance of orphan genes as possible drivers for venom evolution.

## Introduction

The predominant scenario for the evolution of a new gene function presumes that gene duplication is followed by neo- or sub-functionalization of 1 of the copies, resulting in a novel gene function [1, 2]. To differentiate mechanisms of gene origin, a larger taxon sampling and high quality of utilized whole-genome data are mandatory. This objective is now more achievable because of the fast development in next-generation sequencing technology. However, whole-genome data for comparative analyses are still sparse in evolutionary venomics (Supp. Table 1) and, as a consequence, the relative importance of the underlying mechanisms in the evolution of venom proteins and peptides remains to be addressed in more detail.

Venoms have evolved across a wide range of animal lineages as important evolutionary traits that are used for predation, defense, or competition [3–6]. They are cocktails of bioactive molecules that are usually composed mainly of peptides and proteins, collectively referred to as “toxins,” that often exhibit a variety of pharmacological properties linked to their toxicity. These venom proteins and peptides have evolved new toxic functions from non-toxic ancestral versions, and they are thus ideal candidates to test classical hypotheses on the evolution of new gene functions.

However, only a few comparative studies based on whole-genome data have explored the different mechanisms that instigate the origin of toxin genes. In general, toxin evolution by gene duplication represents a widely accepted hypothesis and receives support as a major mechanism of toxin origin from genomic analyses of the king cobra (*Ophiophagus hannah*), the Chinese scorpion (*Mesobuthus martensii*), and the Brazilian white-knee tarantula (*Acanthoscurria geniculata*) [7–9]. In contrast, analyses of the genomes of the platypus (*Ornithorhynchus anatinus*) and parasitic wasps (*Nasonia vitripennis, Trichomalopsis sarcophagae*) found that in these lineages, co-option of single-copy genes reflects the dominating process that shapes toxin evolution [10, 11]. Nevertheless, the available genomes of venomous taxa often reflect improper sampling densities of the respective lineages (Supp. Table 1). As a consequence, there is a need for comparative approaches, which add more genome data to clades of interest and suitable outgroups, to provide a better understanding of general processes in toxin evolution.

In this study, we examine the processes that drive toxin evolution in robber flies (Asilidae, Diptera), which is one of the largest extant fly groups and includes >7,000 species [6, 12]. Asilids are also the only known clade within dipteran insects in which both sexes use venom for an adult predatory lifestyle [6, 12]. We first characterized the venom system of male and female specimens of *Dasypogon diadema* using a combination of functional morphology, venom gland transcriptomics, and venom proteomics. *D. diadema* is of particular interest because it specializes in hunting hymenopterans, which possess venom that can be used in defense and thus represent potentially dangerous prey [13, 14]. We also utilized transcriptome and proteome data from the venom of 2 additional European asilids (*Eutolmus rufibarbis* and *Machimus arthriticus*) to determine major venom components in robber flies [12], and compared our results with a third, recently published study of the Australian giant robber fly (*Dolopus genitalis*) [15].

The mechanisms by which the identified venom proteins evolved in *D. diadema* were subsequently inferred by performing an extensive comparative genomics analysis. To reveal the evolutionary origin of asilid venom proteins, we sequenced, assembled, and annotated a high-quality draft genome of *D. diadema*, and co-annotated a recently published genome of the asilid *Proctacanthus coquillettii* [16]. We then compared these to publicly available high-quality genomes of 10 dipteran and 2 lepidopteran model organisms. Our results reveal a complex, multimodal pattern for the origin of venom proteins, and that the venom of *D. diadema* evolved dynamically through mechanisms that include both gene duplication and single gene co-option. The venom proteins partly originate from genes with ancestral variants already present in the protein-coding genome of the last common ancestor (LCA) of Diptera and Lepidoptera. Other putative toxins are lineage-specific to robber flies and show no detectable homologues outside the asilid genomes. Our results are based on the largest comparative genomics data set in evolutionary venomics to date and demonstrate the potential and necessity of comparative genomics to understand venom evolution in a broader context.

## Results

### The venom system of*Dasypogon diadema*

**Fig. 1:**
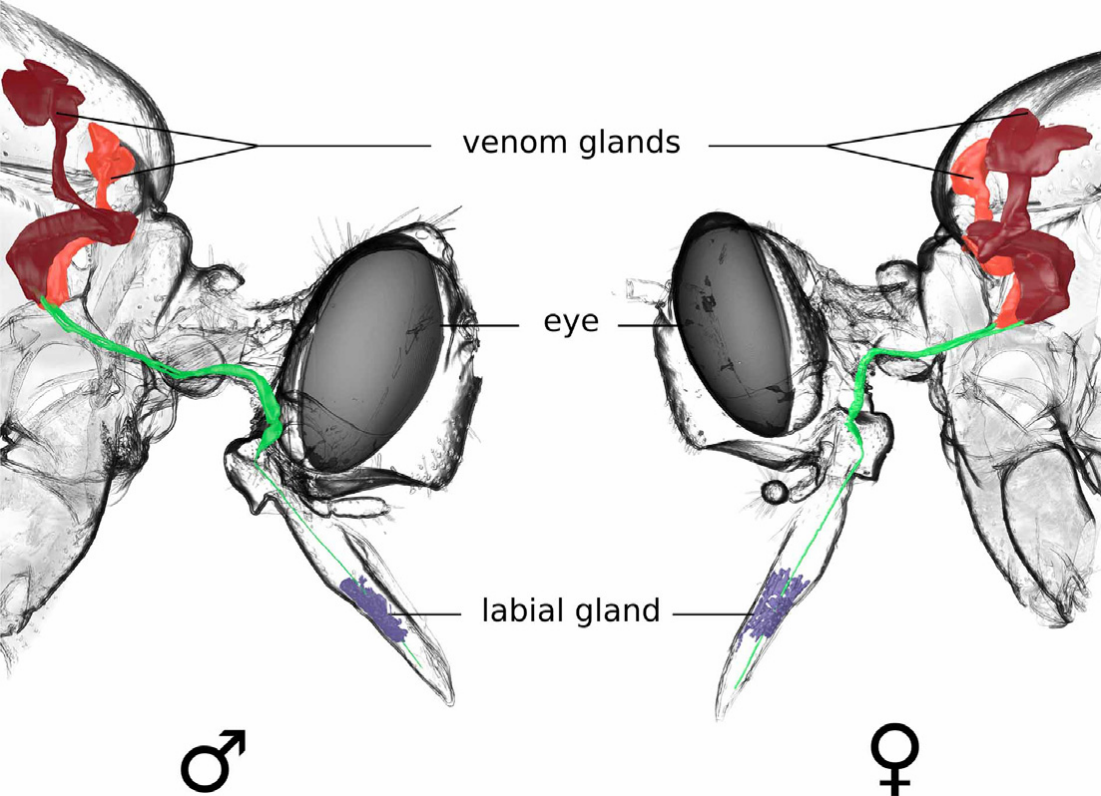
The 3D reconstructed venom delivery system of female and male *Dasypogon diadema*. The general anatomy of *D. diadema* is similar between both sexes and to the structures described for *Eutolmus rufibarbis*.A pair of elongated sac-like glands located in the first and second thoracic segments (right and left glands coloured red and orange, respectively) open separately into ducts (coloured green), which fuse just before entering the head capsule and continue to the tip of the proboscis. Compared with the glands of *E. rufibarbis*, the glands of *D. diadema* are more elongated, featuring a larger volume and sub-compartmentalization. The labial glands (coloured blue) are located in the middle part of the proboscis and open into the lumen between theca and the labium at the tip of the proboscis.

To compare the venom delivery system of *D. diadema* with those of previously described asilid species, we examined the morphology of its venom apparatus by performing synchrotron-based microcomputer tomography reconstructions of both a male and a female specimen. We found no differences between the compared male and female specimen of *D. diadema;* however, to discount sexual dimorphism in asilid venom systems, this result should be combined with a larger sampling size per sex for definite conclusions. The venom apparatus of *D. diadema* appears generally similar to the previously described structures of *E. rufibarbis* [12], with the exception that the venom apparatus of *D. diadema* features more complex and elongated, sub-structured thoracic venom glands (Fig. 1).

**Fig. 2:**
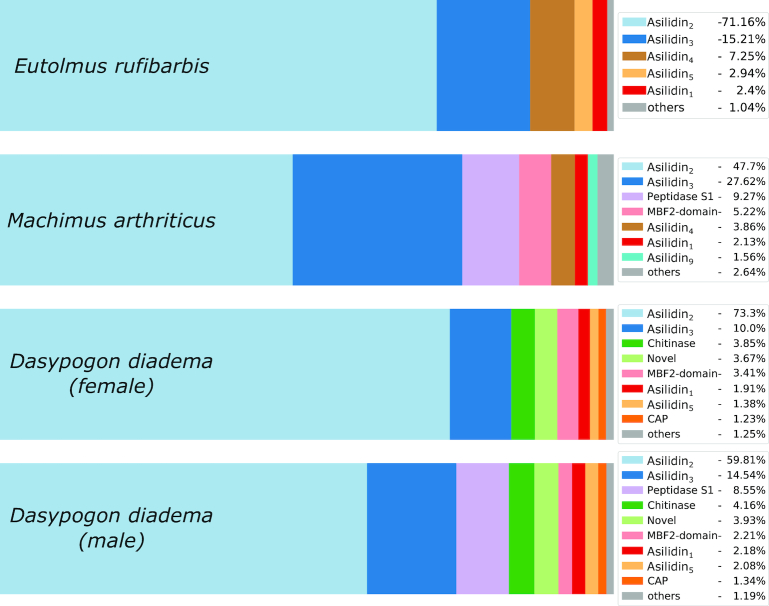
Relative expression of putative toxin families in *Dasypogon diadema* (male and female), compared to *Eutolmus rufibarbis* and *Machimus arthriticus*. The expression levels of protein families secreted in the venom glands are given in percent. Only sequences with matches from proteomics and a threshold >1 transcripts per million (TPM) are included. Protein classes with an expression value <1% of the depicted TPM are summarized in the category “others.”

Complementing our morphological analysis, the venom composition of *D. diadema* was investigated by applying a combination of venom gland, proboscis, and body tissue transcriptomics and a proteomic analysis of venom gland extracts from both sexes. Apart from a more complex morphology, the venom cocktail of *D. diadema* showed a number of differences compared with the described venom of *E. rufibarbis* and *M. arthriticus* [12]. The most striking disparity is that the venom of *D. diadema* contained chitinase-like proteins and proteins that belong to the catabolite gene activator protein (CAP) superfamily, which were absent in the venoms of *E. rufibarbis* and *M. arthriticus* (Fig. 2). The expression level of transcripts coding for chitinase-like proteins were ranked third (female) and fourth (male) among all identified venom proteins (male: TPM, 4.16%; female: TPM, 3.85%, percentage of the summed TPM value of all identified venom proteins), while CAP-like proteins were expressed at a comparably low level in both sexes (male: TPM, 1.34%; female: TPM, 1.23%) (Fig. 2). We also identified 5 families of novel venom proteins among the 30 predominant putative toxins, which we named asilidin_11–15_, according to existing robber fly toxin nomenclature [12, 17] (Figs 2, 4, Supp. Table 2 and Supplementary File 4). Last, we identified peptidase S1 in the venom of *D. diadema*, which is also abundant in the venoms of *E. rufibarbis* and *M. arthriticus*.

While we observed differences between species, there were also a number of families with similar expression levels across the examined species, which we define as major venom components of asilids. One such component is the previously described family asilidin_1_ (*E. rufibarbis*: 2.4%; *M. arthritcus*: 2.13%; female *D. diadema*: 1.91%; male *D. diadema*: 2.18%) [12]: its putative cysteine inhibitor knot peptides (ICKs) were shown to have neurotoxic effects on the European honeybee (*Apis mellifera)* [12]. As for *E. rufibarbis* and *M. arthriticus*, we also identified members of the asilidin_5_ family and MBF2-domain–like proteins in the venom of *D. diadema*. However, the 2 most dominantly expressed venom gland protein families for all species are asilidin_2_ and asilidin_3_, which account for 75% (*M. arthriticus*), 75% (male *D. diadema*), 83% (female *D. diadema*), and 86% (*E. rufibarbis*) of the toxin-assigned TPM values (Fig. 2).

### Genome data quality and completeness

To assess the evolutionary origin of the venom proteins of *D. diadema*, we combined the protein-coding genome of high-quality genomes from Diptera and Lepidoptera with our venomic data from the female and male specimen of *D. diadema* (Table 1; Supp. Table 2) [18, 19]. We also used our venomic data to re-annotate the first high-quality robber fly genome, of *P. coquillettii* [16], and to annotate the *D. diadema* genome sequenced and assembled in the present study (Table 1; Supp. Table 3, accession numbers of SRA and BioSample entries for transcriptome and genome data are linked to the BioProject PRJNA361480, see also Data availability section). Both robber fly genome annotations were refined by including all transcriptomic and proteomic data of asilid venom glands during the annotation.

**Table 1:** Overview of all analysed genomes and their gene completeness

Order	Species	No. of analysed CDSs	BUSCO completeness (%)	
Lepidoptera	*Bombyx mori*	14,623	84.5	***
	*Danaus plexippus*	15,128	94.8	***
	*Culex quinquefasciatus*	19,032	89.9	***
	*Aedes aegypti*	17,158	95.5	***
	*Anopheles gambiae*	14,916	98.6	***
	*Anopheles darlingi*	10,519	90.1	***
	*Mayetiola destructor*	22,410	86.7	***
Diptera	*Dasypogon diadema*	15,480	91.1	*
	*Proctacanthus coquillettiii*	10,942	96.7	**
	*Drosophila grimshawi*	19,429	99.4	***
	*Drosophila melanogaster*	30,429	99.7	***
	*Drosophila simulans*	24,119	99.2	***
	*Teleopsis dalmanni*	16,570	68.9	***
	*Lucilia cuprina*	14,452	91.7	***

To infer the quality of the annotation, a BUSCO analysis was performed using the transcriptome mode and the holometabolous dataset.

^*^Genome was sequenced and annotated for this study.

^**^Genome from Dikow et al. [16] was reannotated.

**Fig. 3:**
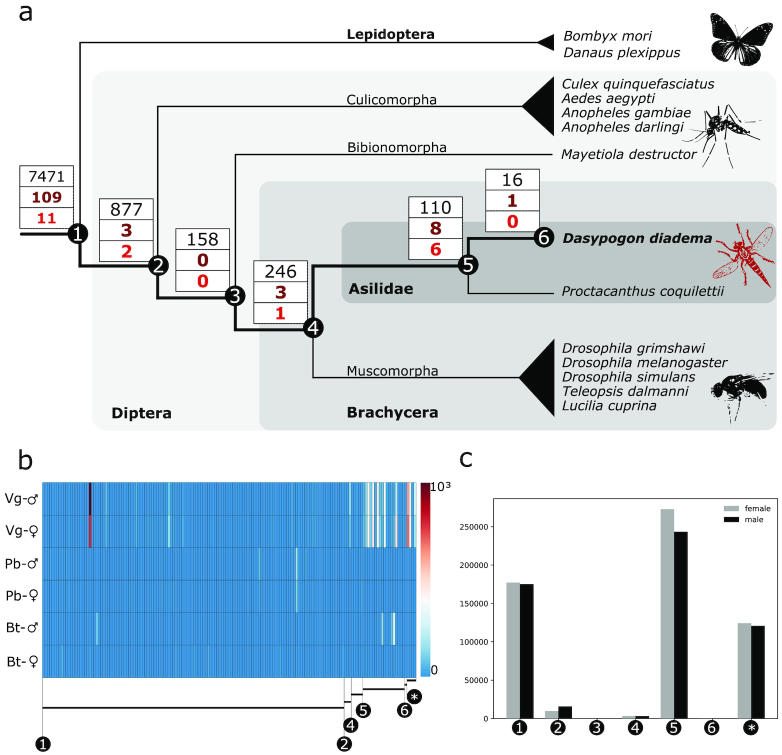
(a) Phylogenetic relationships of the included taxa. *Dasypogon diadema* was used as the focal species for the analyses of the orthogroups. Boxes on the split show the number of orthogroups shared by *D. diadema* and the respective clade of the split (upper number: number of shared orthogroups; middle number: number of orthogroups with putative toxins; lower number: number of orthogroups associated with the 30 predominant putative toxins). (b) Heat map showing the expression level (TPM) in the 3 tissues of the putative toxins of both sexes. The white numbers in the black circle refer to the affiliated orthogroups and splits in 3a (Vg-♂: venom gland male; Vg-♀: venom gland female; Pb-♂: proboscis male; Pb-♀: proboscis female; Bt-♂: body tissue male; Bt-♀: body tissue female). (c) Summarized expression level (TPM) of the putative toxin transcripts in the venom gland of both sexes. The white numbers in the black circle refer to the affiliated orthogroups and splits in 3a (number of putative toxins for all nodes: Node 1: 130; Node 2: 3; Node 3: 0; Node 4: 5; Node 5: 18; Node 6: 1; *no orthogroup: 4).

^***^Protein dataset from ENSEMBL. The order of the species in this table matches the species order in the cladogram in Fig. 3a.

Gene sets of dipterans and lepidopterans obtained from ENSEMBL scored a 68.9–99.7% completeness when analysed with BUSCO (Table 1) [20, 21]. The presented sets of protein-coding genes of the robber flies *P. coquillettii* and *D. diadema* match this range, scoring 96.7% and 91.1% completeness, revealing high-quality annotations and assembly completeness (Table 1).

### Assessing ancestral gene variants

The protein-coding genomes of *D. diadema* and *P. coquillettiii*, 10 non–robber fly dipterans, and 2 lepidopterans were compared and sorted using the Orthofinder pipeline (Table 1) [18]. Orthofinder performs a BlastP similarity search followed by normalization for sequence length, creation of an orthogroups graph, and Markov cluster algorithm clustering to sort the genes according to their likeliest homology relationships. The recovered orthogroups comprise protein-coding genes that originated from a single gene in the LCA of all analysed species or lineage-specific genes in a certain clade. An orthogroup can comprise several or only parts of a single gene family, which might change with the analysed taxa and the depth of the considered evolutionary splits. Genes without homologues in any of the included genomes cannot be assigned to orthogroups.

The final annotation of the *D. diadema* genome consists of 15,480 protein-coding genes, of which 13,981 genes were sorted into 8,878 orthogroups. The remaining 1,499 protein-coding genes did not match any of the assigned orthogroups (Fig. 3a, Supp. File. 2). In our analysis *D. diadema* served as the focal organism; the origin of the protein-coding genes was inferred from their first-time emergence. For instance, genes of *D. diadema* with homologues in the lepidopterans *Bombyx mori* or *Danaus plexippus* or both were assigned to originate in the LCA of Diptera and Lepidoptera, or earlier. Following this concept, orthogroups were sorted to the considered phylogenetic splits (Fig. 3a).

The split between the Diptera and Lepidoptera lineages is the oldest one considered in our analyses. These 2 clades share 84% (7,471) of the orthogroups assigned to *D. diadema* (Fig. 3) [22], meaning the ancestral versions of these protein-coding genes already existed in the LCA of the dipteran and lepidopteran clade. Of the remaining orthogroups, 877 are unique for the clade of Diptera, 158 are unique for the split between the gall midge *Mayetiola destructor* and the brachyceran clade, 246 are unique for Brachycera, and 110 orthogroups are shared only between the 2 robber flies (Fig. 3a). Sixteen orthogroups are constituted of protein-coding genes found exclusively in *D. diadema* (Fig. 3a).

The venom gland proteins identified via proteomics were sorted to their associated orthogroups. We then tested whether the non-toxic ancestral version of a putative toxin was already present in the protein-coding genome of the LCA of the compared species, or whether the protein is a unique novelty for a certain clade. A total of 109 orthogroups, which were already present in the LCA of Lepidoptera and Diptera, are associated with ≥1 venom protein of the female and male *D. diadema*. Three orthogroups with venom proteins were unique to each of Diptera and Brachycera, while 8 orthogroups with putative toxins were shared only between the 2 robber fly genomes (Fig. 3a). The majority of proteins identified in the venom gland can be assigned to protein-coding genes present in the orthogroups shared between the Lepidoptera and the Diptera clade. The transcripts of venom proteins assigned to orthogroups, which arise on Node 2, Node 3, or Node 4, are expressed on a low level in the venom glands of both sexes. Putative toxin transcripts of Node 1, Node 5, and the ones assigned to no orthogroup are expressed on a high level in the venom glands of both sexes (Fig. 3b and c, Supp. Figs 3 and 4).

### Evolutionary pattern of the predominant venom proteins

To prevent an over-interpretation of the data, the process of venom evolution in *D. diadema* based on whole-genome data was analysed by using a stricter threshold and focusing exclusively on the dominant putative toxin transcripts. For this purpose, we included only putative toxin transcripts that were detected via proteomics, display an expression level in the venom gland of ≥500 TPM, and show a 4-fold higher expression level in the venom gland compared with the respective body tissue. Two independent tools (Segemehl and Salmon) were applied to perform the RNA quantification and to test the robustness of the results [23, 24]. Both quantification approaches using identical thresholds reveal similar results. All 28 putative toxin transcripts identified via Segemehl were also identified with Salmon. Salmon, however, reported 2 further transcripts that still met the threshold. Further downstream analyses were based on the results from the quantification with Salmon, which resulted in a top 30 set of predominant putative toxins that are discussed further (Fig. 3b and c, Supp. Figs 3 and 4).

**Fig. 4:**
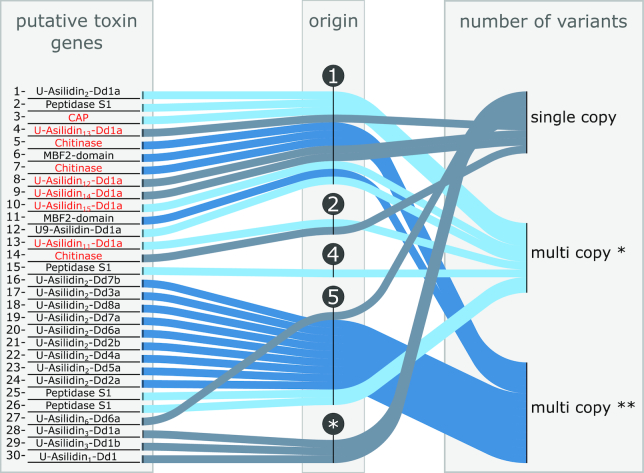
The evolutionary pattern and the origin of the top 30 putative toxins. The node numbering refers to the nodes in Fig. 3a. Putative toxins present in *Dasypogon diadema* but missing in *Eutolmus rufibarbis* or *Machimus arthriticus* are coloured red. Single-copy genes: putative toxins with only 1 copy on the protein-coding genome of *D. diadema*; multi-copy genes*: protein-coding genes that belong to orthogroups assembled of ≥2 protein-coding genes in *D. diadema*. Only 1 member of the orthogroup is present in the venom; multi-copy genes**: protein-coding genes that belong to orthogroups assembled of ≥2 protein-coding genes in *D. diadema*. Two or more members of the same orthogroup are present in the venom.

For 3 of those top 30 predominant putative toxins (U-Asilidin_3_-Dd1a, U-Asilidin_3_-Dd1b, and U-Asilidin_1_-Dd1a) no orthogroup was assigned, suggesting that these genes are unique to *D. diadema* (Figs 3 and 4, Supp. File 3, Supp. Table 6). The remaining 27 putative toxin transcripts were distributed among 20 different orthogroups (Supp. Table 6, Supp. File 3). While 11 of these orthogroups are shared between the lepidopteran and dipteran clade, 2 orthogroups are unique to the dipteran clade, 1 to the brachycerans, and 6 are shared only between the asilids. In general, 22 putative toxins can be categorized as multi-copy genes (Fig. 4). They are distributed between 15 different orthogroups, each composed of ≥2 protein-coding genes of *D. diadema*. Five of these groups contain 2 or more of the 30 predominant putative toxins. In 2 orthogroups (OG009368, OG0011154), all members are putative toxins and are present in the venom gland (Supp. Table 6). For 10 orthogroups, only 1 member is a putative toxin present in the venom gland while the others are not. The newly identified putative toxins U-Asilidin_12_-Dd1a, U-Asilidin_13_-Dd1a, and U-Asilidin_14_-Dd1a are all single-copy genes, while the U-Asilidin_11_-Dd1a and U-Asilidin_15_-Dd1a are categorized as multi-copy genes (Supp. Table 6).

Members of the asilidin_2_ protein family are distributed across 4 different orthogroups; 3 of these are shared only between *D. diadema* and *P. coquillettii*, while the remaining 1 is shared between the Lepidoptera and Diptera (Fig. 4). A similar picture is revealed in larger protein families like PS1 and chitinase-like, for which distinct versions of putative toxin from different orthogroups were identified (Fig. 4, Supp. Table 6).

### Transposable elements

Transposable elements were identified in 11 of the 30 predominant toxins of *D. diadema*, including the protein families asilidin_2_, peptidase S1, chitinase, MBF2-domain, asilidin_6_, asilidin_9_, asilidin_11_, asilidin_12_, asilidin_13_, and asilidin_15_ (Supp. Table 7). In the dominant component asilidin_2_, the variants U-Asilidin_2_-Dd1a and U-Asilidin_2_-Dd2a harbour transposable elements in the intron sequence. In contrast, no gene variants classified as asilidin_3_, the second most highly expressed venom component, feature transposable elements. The majority of the transposable elements resemble retrotransposons classified as long terminal repeat retrotransposons of currently unknown groups. Other identified elements are retrotransposons classified as long interspersed nuclear elements and DNA-transposons classified as Mariner-like elements (Supp. Table 7).

## Discussion

### General aspects on the venom biology and composition

*D. diadema* is a widely distributed robber fly that is known to hunt honeybees (*A. mellifera)* and other hymenopterans [13, 14]. To overpower such dangerous prey, venom with neurotoxic components for rapid paralysis is advantageous. Trophic specialization has also been shown to affect venom composition and even venom apparatus morphology in other predatory venomous lineages, such as snakes [25, 26] and spiders [27]. We therefore expected the venom composition of *D. diadema* to contain substantial differences compared to the previously studied, more generalist species *E. rufibarbis* and *M. arthriticus*. Indeed, their venoms differ in some aspects, such as the presence of chitinase and CAP proteins in *D. diadema*, which were not detected in the venoms of *E. rufibarbis* and *M. arthriticus*. Similar to *D. diadema*, the venom composition of the Australian robber fly *Dolopus genitalis* also appears to contain a larger fraction of enzymatic proteins than those of *E. rufibarbis* and *M. arthriticus* [15]. *D. genitalis* venom also contained all asilidin families and major venom components that we discuss here [15]. Last, Asilidin_2_ is an especially highly expressed component in all asilids, including *D. genitalis*. The observed slight sex-specific variation of the venom composition in our pooled samples of male and female individuals might be explained by the known differing ecology of males and females. However, this hypothesis is speculative and requires further testing with additional replicates.

In general, the venom of *D. diadema* shares the major components with *E. rufibarbis* and *M. arthriticus*. Additionally, the most dominant protein families in the venoms of all 3 species are asilidin_2_ and asilidin_3_, and all species also express asilidin_1_ transcripts (Fig. 2). The phylogenetic distance between *E. rufibarbis*and*M. arthriticus* (members of the larger subfamily Asilinae) compared to *D. diadema* (representative of the subfamily Dasypogoninae) [16, 28] suggests that these 3 protein classes resemble a lineage-specific toxin arsenal of robber flies, a conclusion that is corroborated by the study of Walker and colleagues [15].

In the present study the *de novo* assembly of transcriptome data was performed using a single assembler, Trinity, which is one of the most established programs to assemble transcriptome data sets [29]. Nevertheless, *de novo* transcriptome assembly is challenging and different assembly software packages often construct differing sets of transcripts. It has been shown in snakes and scorpions that the number of assembled toxin transcripts may vary depending on the chosen assembler [30]. Thus, applying only 1 assembler as a base for our analyses may mean that some of our putative toxins may include false-positive results and that we might have missed some toxins that represent false-negative results.

To avoid false-negative results and an over-interpretation of our data, we used only transcripts that were recovered in the proteome and then identified in the whole genome as baseline to discuss possible toxins. We also used 2 additional transcriptome assemblers, rnaSPAdes [31] and Trans-ABySS [32], and assessed their ability to recover our top 30 predominant toxins identified using Trinity. Except for a few candidates, the majority of the top 30 candidate toxins were recovered with identical or highly identical sequence similarity in the additional assemblies. Our conclusion is therefore that the pattern of venom protein evolution that we discuss here for the most highly expressed, and hence ecologically probably most important, putative toxins is rather robust. (All details are shown in Supplementary Tables 8 and 9, and all visualized alignments comparing the contigs from different assemblers are provided in the GigaScience data cloud).

Determining the frequency of false-negative results would require extensive additional work: specifically, using multiple other *de novo* assemblers on all the data to see whether anything had been missed in the Trinity assembly. In principle, because our toxin evolution findings were attained using analyses on only the top 30 identified toxins, the impact of false-negative results on our findings is likely to be limited. However, if any missed (false negative) toxins have to be added to our current top 30 toxins, our conclusions could be affected. Additional details on the processes of venom evolution in robber flies will also be revealed by further genome data and deeper, more detailed proteomic analyses of milked venom from single specimens.

### The evolution of the neurotoxic component asilidin_1_

Asilidin_1_ peptides resemble a cystine inhibitor knot-like fold (ICK), and 1 representative, U-asilidin_1_-Mar1a, was shown to induce neurotoxic effects on the European honeybee (*A. mellifera)* [12]. Facilitating a fast and efficient paralysis of prey, asilidin_1_ probably represents a biologically important component in robber fly venom. ICK peptides have been convergently recruited as neurotoxic venom components in a range of venomous lineages, including scorpions, spiders, assassin bugs, cone snails, and possibly also remipede crustaceans [33, 34, 43, 35–42]. The identification of ancestral versions of short neurotoxins, such as ICK peptides, that feature a conserved cysteine scaffold with variable positions between the cysteines remains a challenge [38]. Indeed, while our complementary proteomic and transcriptomic analyses of the venom gland proteins of *D. diadema* revealed 3 different asilidin_1_ variants, only 1 protein-coding gene was detected at the genome level (U-Asilidin_1_-Dd1a). The U-Asilidin_1_-Dd1a gene is not a member of a gene family with several duplicates but represents a single-copy gene. Differences in the coding sequences derived from transcriptome data thus likely reflect allelic variation in specimens that had to be pooled for proteome and transcriptome analyses to achieve sufficient tissue quantities. This finding highlights the possible bias of predicting toxin diversity in data from pooled samples.

### General patterns of venom protein evolution

The evolutionary origin of the major venom proteins in *D. diadema* can be classified into 2 major categories. The first category comprises variants of both single- and multi-copy genes with ancient origin. These robber fly toxins have homologous genes in the lepidopterans or non-asilid dipterans, and originate from ancestral protein versions, which occur in the LCA of asilids and the respective clade.

Four single-copy genes of the protein families asilidin_12_ (U-Asilidin_12_-Dd1a), asilidin_13_ (U-Asilidin_13_-Dd1a), asilidin_14_ (U-Asilidin_14_-Dd1a), and chitinase with homologues outside the asilid clade provide examples of venom protein evolution without gene duplication. These genes (13.3% of the predominant venom proteins) most likely feature an expression-dependent single gene co-option–type functional recruitment. Under this scenario, an up-regulation of expression in the venom gland tissue and the injection of the otherwise physiological protein as a venom component might lead to a toxic effect in the prey species. In contrast, putative toxins of the protein families asilidin_2_, asilidin_9_, CAP, chitinase, peptidase S1, and MBF2-domain–like proteins are present as multi-copy genes. The revealed pattern of 1 or more duplication events in the history of these genes supports the widely proposed hypothesis of toxin evolution by gene duplication [3, 4, 44].

The second category of venom proteins includes putative toxins without homologues outside the asilid lineage. Multi-copy genes dominate this category (asilidin_2_, peptidase S1), although single-copy genes are also present (asilidin_6_). Particularly asilidin_2_ shows a pattern of intense gene duplication, and several transcripts in this family from different orthogroups are secreted in the venom glands. These single- and multi-copy genes are robber fly lineage-specific and their ancestry is enigmatic. Intriguingly, we identified transposable elements in 11 venom proteins, including 2 variants of the highly expressed asilidin_2_. Two-thirds of the venom proteins do not show any presence of transposable elements. We can only speculate here that the evolution of single toxins might be influenced by transposable elements and that this might be an explanation for the diversity of asilin_2_ variants. However, to provide a profound analysis on the influence of transposable elements on the evolution of venom proteins, the analysis design needs to be adapted and whole-genome data and venom protein data of more species needs to be included.

## Conclusion

The insects include several venomous lineages and comprise the greatest number of venomous species within the animal kingdom [4]. For many of these, the venom compositions and putative toxins remain unknown [6]. Besides hymenopteran and heteropteran taxa, insects also harbor predatory and venomous asilid dipterans. Despite some differences between studied species, our results suggest that the major components of asilid venom constitute new putative toxins that are likely to be restricted to asilids. These include the asilidin_1_ family, which contains the recently described neurotoxic component U-asilidin_1_-Mar1a, and has been identified in all 4 studied asilid venoms, including *D. diadema* (U-Asilidin_1_-Dd1a) [12, 15].

The present study includes the currently most comprehensive species set of genomes to assess the evolution of venom proteins in *D. diadema* as a representative in the previously uncovered dipteran lineage of robber flies. Our analysis is further strengthened by the implementation of gene-sets from model organisms and closely related species, maximizing our ability to detect toxin homologues and identify the processes that underlie their evolution. This approach revealed that the processes that contribute to the evolution of toxins in *D. diadema* venom are multimodal and include (i) expression-depending co-option of housekeeping genes, (ii) neofunctionalization after gene duplication events, and (iii) highly expressed lineage-specific orphan genes. Intriguingly, several of these lineage-specific genes of venom proteins remain of enigmatic origin. The role of these orphan genes as possible drivers in venom evolution represents an intriguing topic for future studies. Our findings highlight the value of studying neglected venomous lineages to improve our understanding of the evolution of venoms and their toxins, and hence the evolutionary mechanisms involved in the evolution of protein function.

## Methods

### Robber fly collection and sample preservation

Specimens were collected in June 2014 in France at the riverbanks of the river Têt north of Millas in the Département Pyrénées-Orientales (Occitanie) and the vineyards around Brûlat in the Département Var (Provence-Alpes-Côte d'Azur). For transcriptome sequencing, samples from body tissue, thoracic gland tissue, and proboscis tissue of 6 males and 6 females were separately dissected and preserved in RNAlater (Ambion, Thermo-Fisher, Waltham, MA, USA). All dissected individuals were preserved in 94% ethanol as voucher specimens. In addition, thoracic glands from 7 males and 5 females were crushed after dissection in 1× phosphate-buffered saline buffer with proteinase inhibitor tablets (Complete Ultra, ROCHE, Mannheim, Germany) for proteomic work. See also Supplementary Fig. 5 for the general workflow. Two individuals for both sexes were deposited in Bouin liquid to perform synchrotron-based microcomputer tomography.

### Venom apparatus

The functional morphology of the venom delivery system in both sexes of *D. diadema* was investigated using synchrotron-based microcomputer tomography. Bouin-preserved samples were critical point dried, mounted on a specimen holder, and scanned at the Swiss Light Source electron synchrotron accelerator. Morphological structures were segmented in aligned image stacks using ITK-snap v.3.60 [45]. The visualization of the reconstructed 3D model was carried out using Blender v.2.79 [46].

### Transcriptomics

Total RNA of thoracic glands, proboscis tissue, and body tissue was extracted following the standard protocol for Trizol Reagent by Thermo Fisher, Waltham, MA, USA. For both sexes, the gland and proboscis tissues of 6 specimens were pooled to guarantee sufficient RNA quantity, while body tissue was extracted from 1 individual per sex. All 6 samples for male and female *D. diadema* specimens were prepared for sequencing at the Core Unit DNA Technologies of the University of Leipzig using the Illumina poly-A selection protocol. Sequencing was performed on the Illumina HiScanSQ platform with 100 bp paired-end reads (Supp. Table 5). All generated data are accessible via the BioProject PRJNA361480, including all BioSample and SRA entries (see also Supp. Table 4). In addition to our own data, all available asilid transcriptomes were mined in the SRA archive for later genome annotation (Supp. Table 4). All transcriptome raw reads were processed in the same way after visual inspection in FastQC [47]. Quality filtering and trimming was then applied in Trimmomatic v.033 with a minimum length of 60 bp and a minimum phred score of 30 [48]. All pre-processed datasets were finally assembled using Trinity v.2.4 with default settings except a minimum contig length of 138 [29]. The transcript abundance in all *D. diadema* tissue samples was estimated by mapping the trimmed RNA reads with Segemehl (alignment accuracy, 98%) [24, 49] and by comparatively quantifying reads with Salmon (default settings). The TPM (transcripts per million) values for each coding domain sequence were visualized with a customized Python script and the Seaborn package; see also Identification of venom proteins section.

### Proteomics

The lyophilized venom from the thoracic glands preserved in proteinase inhibitor was dissolved in water and prepared for proteomic analysis as described in Drukewitz et al. [12]. Briefly, the samples were desalted by means of acetone precipitation, proteins reduced with dithiotheitol, alkylated with iodoacetamide, and digested by overnight incubation with trypsin. The digested venom was desalted using a C18 ZipTip (Thermo Fisher, Waltham, MA, USA), dried in a vacuum centrifuge, and dissolved in 0.5% formic acid before 2 µg of each sample was analysed by liquid chromatography with tandem mass spectrometry (MS/MS) on an AB Sciex 5600TripleTOF (Framingham, MA, USA) equipped with a Turbo-V source heated to 550°C and coupled to a Shimadzu Nexera UHPLC (Kyoto, Japan). The digested venom was fractionated with an Agilent Zorbax stable-bond C18 column (2.1 × 100 mm, 1.8 μm particle size, 300 Å pore size), across a gradient of 1–40% solvent B (90% acetonitrile, 0.1% formic acid) in 0.1% formic acid over 60 min, using a flow rate of 180 µL/min (all solvent concentrations are in volume to volume). MS1 survey scans were acquired at 300–1,800 m/z over 250 ms, and the 20 most intense ions with a charge of +2 to +5 and an intensity of ≥120 counts/s were selected for MS2. The unit mass precursor ion inclusion window was ±0.7 Da, and isotopes within ±2 Da were excluded from MS2, which scans were acquired at 80–1400 m/z over 100 ms and optimized for high resolution.

For protein identification, MS/MS spectra were searched against sequence lists consisting of both the translated venom gland and body transcriptomes of *D. diadema* using ProteinPilot v5.0 (AB Sciex, Framingham, MA, USA). Searches were run as thorough identification searches, specifying urea denaturation, tryptic digestion, and cysteine alkylation by iodoacetamide. Amino acid substitutions and biological modifications were allowed in order to identify potential post-translational modifications and to account for chemical modifications due to experimental artefacts. Decoy-based false-discovery rates (FDRs) were estimated by ProteinPilot, and for our protein identification we used a protein confidence cut-off corresponding to a local FDR of <0.5%. Spectra were also manually examined to further eliminate any false-positive results.

### Genome sequencing and assembly

DNA was extracted from 30 mg of muscle tissue of a female specimen of *D. diadema*. The tissue was dissolved in 500 µL lysis buffer (10mM Tris-HCl pH 8, 0.5% [w/v] sodium dodecyl sulfate, 2.4 mg/mL proteinase K, 1mM ethylenediaminetetraacetic acid [EDTA] pH 8) for 50 min at 50°C while shaking. Chitinous debris was spun down in a table centrifuge, and the DNA was extracted from the supernatant using MinElute silica spin columns (MinElute PCR Purification Kit, Qiagen, Hilden, Germany) according to the manufacturer's specifications. Two aliquots of 3 µg isolated DNA were sheared to 200 and 400 bp average length in a Covaris S220 Focused Ultrasonicator (200 bp settings: 10 dc, 5 i, 200 cpb, fs 180 s; 400 bp settings: 10 dc, 4 i, fs 55 s). 100 ng sonicated DNA served as input for library preparation as described by Meyer and Kircher [50]. Both libraries were double-indexed with 2 unique barcodes of 7 bp and amplified as described by Kircher et al. [51]. Paired-end reads were subsequently sequenced with 150 bp on an Illumina MiSeq platform. All raw reads were visually inspected in FastQC [47] and then quality filtered and trimmed applying Trimmomatic v.033 with a minimum length of 70 bp and a minimum phred score of 30 [48]. An overview of sequenced raw reads and processed transcripts is given in Table 2.

**Table 2:** Overview of DNA libraries generated for the *Dasypogon diadema* genome assembly

Library name	Fragment length (nt)	No. of sequenced read pairs	Theoretical genome coverage (fold)
D1130	200	9,119,970	5
D1131	400	167,137,385	89

Number of read pairs and fragment size of the libraries used for the genome assembly are shown. The theoretical genome coverage was calculated with a genome size estimate of 450 Mb and a read length of 120 nt (nucleotides) after processing.

The genome assembly was performed with Maryland Super Read Cabog Assembler (MaSuRCA) v.3.1.3 with the linking mates option set to 1 and the cgwErrorRate set to 0.15; all other options were default [52]. To inspect the quality and to exclude possible contamination Blobtools was applied [53]. The final assembly resulted in an overall assembly size of 450 Mb (scaffold > 2 kb), with an N50 of 32.6 kb and a guanine-cytosine content of 35.81%. Assembly size, N50 value, and other statistics were assessed with Quast v.4.6 [54]. The final genome size is in line with the prior estimated size via *k*-mer distribution using jellyfish [55], which resulted in 427 Mb (Supp. Fig. 2). The assessment with BUSCO (genome mode, holometabolous core gene set) resulted in 92.4% completeness and a duplication rate of 2.7%, which indicates a high quality of the draft genome of *D. diadema* and that the heterozygous areas were adequately assembled [20].

### Genome annotation

Our genome sequence of *D. diadema* was co-annotated with the recently published genome of *P. coquillettii* using the Maker2 pipeline [16, 56]. All *de novo* assembled trancriptome data sets were then utilized to identify splice sites using Exonerate [57] (Supp. Table 3). Additionally, the protein sequences of *Aedes aegypti, Anopheles gambiae, Mayetiola destructor, Lucilia cuprina*, and *Drosophila melanogaster* from the ENSEMBL genome database and all insect proteins from the Swissprot database were aligned using BLAST+ v.2.6.0. Successful aligned positions were extracted to train the gene prediction software Augustus and SNAP [21, 58–60]. The resulting Maker2 gene set after 4 iterative training cycles was finally used for further downstream analyses. The annotation resulted in 10,942 protein-coding genes in the genome of *P. coquillettii* and 15,480 protein-coding genes in the genome of *D. diadema*. The completeness of both gene sets was inferred with BUSCO [20] (transcriptome mode, holometabolous core gene set) and resulted in a completeness of 91.1% for *D. diadema* and 96.7% for *P. coquillettii*(Table 1).

#### Identification of transposable elements

Repetitive elements in the genome of *D. diadema* and *P. coquillettii* were identified using RepeatModeler (v. open-1.0.11); the resulting repeat library was provided to RepeatMasker (v. open-4.07) [61, 62] to mask repetitive elements prior to the annotation of genes. For *D. diadema* the repeatmasker output was parsed with the “One code to find them all” perl tool [63] using the “strict” option. The resulting overview tables were used to analyse the appearance of transposable elements in the top 30 dominant toxins (Supp. Table 7).

### Identification of venom proteins

Putative toxins and venom protein families were identified by applying the approach described in Drukewitz et al. [12]. The strategies for transcriptomics were to perform BlastP searches against ToxProt, to run hidden Markov model searches using HMMER v.3.1b2 [64] against our own venom protein databases, and to characterize highly expressed coding regions. The major difference in the present analysis is that coding domain regions used to identify putative toxins are not derived from *de novo* transcripts but instead based on genome loci that were annotated by transcriptome and proteome sequences. The annotated protein-coding genes of *D. diadema* were matched with the venom gland proteins identified via proteomics applying a strict threshold (e-value of 1e−40, query coverage of 90%). This cut-off was used to reduce false-positive results while at the same time minimizing the number of protein-coding genes that might be missed. The transcript abundance in all *D. diadema* tissue samples was estimated on the basis of the trimmed RNA reads by applying the quantification tool Salmon (default settings) and the read mapper Segemehl (alignment accuracy, 98%) [24, 49]. To assess evolutionary processes of putative toxins a rigorous TPM value of 500 and a 4-fold higher expression level in the venom gland compared to the respective body tissue was picked to prevent over-interpretation of our data.

Additionally, a second threshold with a lower TPM value (>1) was applied to allow a comparison of the identified venom proteins to previously published robber fly data [12]. Proteins with a housekeeping function, a low expression level in the venom glands, and a high expression level in non-venom gland tissue were not considered as putative toxins and excluded from the analysis.

### Venom evolution reconciled by genomics

The ENSEMBL database provides 21 annotated dipteran genomes [21]; 12 of these are from *Drosophila* species. For *Drosophila*, only 3 representative genomes were selected for our analyses (Table 1). Otherwise all available taxa were included, with 2 exceptions. The wingless antarctic midge *Belgica antarctica* was excluded because of its extremely derived lifestyle. *Megaselia scalaris* was excluded because of the rather experimental approach that was used to sequence its genome [65, 66]. The lepidopterans *B. mori* and *D. plexippus* were chosen as outgroup taxa [67, 68]. Apart from ENSEMBL we also mined NCBI for relevant dipteran genomes, and consequently re-annotated and included the genome of *P. coquillettii* (Supp. Table 3) [16].

The protein sets of all analysed genome species were compared and protein-coding genes assigned to orthogroups with Orthofinder [18]. Depending on the taxon samplings, orthogroups can comprise gene families, gene classes, or only parts of such classification. The aim of the approach is not to identify such hierarchical classes but to infer the homology of the analysed protein sets [18, 19]. Under the assumption that orthogroups only arise 1 time but might be lost several times, the origin of novelties and the expansion of protein groups can be analysed. *D. diadema* was used as the focal species, which means that only the orthogroups present in this species were analysed further. An orthogroup is considered as present in the LCA of *D. diadema* and a clade when members of the orthogroup were present in the genome of *D. diadema* and in ≥1 representative of the analysed clade. Shared orthogroups were counted using the Orthofinder output and a customized Python script.

### Use of additional assemblers to assess the top 30 predominant toxins

Venom gland transcriptome datasets of both sexes were additionally assembled using the assembler rnaSPAdes v.3.13.0 [31] and Trans-ABySS v.2.0.1 [32]. Both assemblers were used with the default settings; on those settings rnaSPAdes uses a *k*-mer length of 21 and Trans-ABySS a *k*-mer length of 32. The open reading frames from the initial Trinity assembly and the additionally provided rnaSPAdes and Trans-ABySS assemblies were extracted using Transdecoder v.5.5.0 [69]. Protein sequences of the initial trinity assembly, which are verified via our proteomic analysis and associated with 1 of the top 30 predominant proteins, were used as a query for a BlastP search in the protein sequences of the rnaSPAdes and Trans-ABySS assembly. The protein sequence of the best hit was extracted and aligned with the query sequence using mafft-ginsi. The resulting alignment was visualized using Jalview [70].

## Availability of supporting data and materials

All transcriptome and genome data are available in NCBI via the Bioproject on robber fly venom evolution, PRJNA361480. Transcriptome raw data of male and female venom gland, body, and proboscis tissue are published with the SRA entries SRR7754486, SRR7754485, SRR5192548, SRR5192547, SRR7754488, SRR7754487. The genome assembly is accessible in GenBank under QYTT00000000; the sequencing raw data are stored in the SRA with the 2 accession numbers SRR7878513 and SRR7878512. The mass spectrometry proteomics data have been deposited to the ProteomeXchange Consortium via the PRIDE partner repository with the dataset identifier PXD013358. Other data further supporting this work are available in the *GigaScience* repository, GigaDB [71].

## Additional files

Supplementary Table 1: Overview of available genome species with analysed venom

Supplementary Table 2: Overview of protein families identified in the venom of *D.diadema*

Supplementary Table 3: NCBI Accession to the included genomes

Supplementary Table 4: SRA-archive accession numbers of used RNA and DNA data

Supplementary Table 5: Overview of RNA sequencing and processing of *D.diadema*

Supplementary Table 6: Overview of the predominant top 30 venom proteins and their associated orthogroups

Supplementary Table 7: Overview of transposable elements in the predominant top 30 venom proteins

Supplementary Table 8: Overview alternative assembled transcripts - RNASpades

Supplementary Table 9: Overview alternative assembled transcripts - Transabyss

Supplementary Figure 1: Phylogenetic relationship of the analysed species

Supplementary Figure 2: Histogram of k-mer distribution

Supplementary Figure 3: Summarized expression level of putative toxins

Supplementary Figure 4: Comparison of heatmaps based on the RNA-Seq quantification

Supplementary Figure 5: Overview of the analysis workflow

Supplementary File 2: Overview table of sorted orthogroups for all included species

Supplementary File 3: Overview table of the 30 predominant toxins

Supplementary File 4: Alignments of identified protein families

giz081_GIGA-D-19-00072_Original_Submission

giz081_GIGA-D-19-00072_Revision_1

giz081_GIGA-D-19-00072_Revision_2

giz081_Response_to_Reviewer_Comments_Original_Submission

giz081_Response_to_Reviewer_Comments_Revision_1

giz081_Reviewer_1_Report_Original_Submission Juan Calvete -- 3/22/2019 Reviewed

giz081_Reviewer_2_Report_Original_Submission Mark Margres -- 4/10/2019 Reviewed

giz081_Reviewer_2_Report_Revision_1 Mark Margres -- 5/16/2019 Reviewed

giz081_Supplemental_Files

## Abbreviations

bp: base pairs; BLAST: Basic Local Alignment Search Tool; BUSCO: Benchmarking Universal Single-Copy Orthologs; CAP: catabolite gene activator protein; FDR: false-discovery rate; ICK: inhibitor knot peptide; kb: kilobase pairs; LCA: last common ancestor; Mb: megabase pairs; MS/MS: tandem mass spectrometry; NCBI: National Center for Biotechnology Information; nt: nucleotides; SRA: Sequence Read Archive; TPM: transcripts per million.

## Competing interests

The authors declare that they have no competing interests.

## Funding

SHD is funded by a scholarship (Doktorandenförderplatz) from the University of Leipzig. B.M.v.R. was supported for this work by the German Science Foundation (DFG RE3454/4–1). This work was supported by the Australian Research Council (DECRA Fellowship grant No. DE160101142 and Discovery Project grant No. DP160104025 to E.A.B.U.).

## Authors’ contributions

S.H.D. and B.M.v.R. conceived the project and designed the analyses. S.H.D. and B.M.v.R. performed specimen collection, dissection, and transcriptomic and genomic analyses. E.A.B.U. conducted the proteomic analyses. L.B. performed all laboratory work for the genome sequencing. S.H.D. and B.M.v.R. wrote the manuscript with input from all authors.
