# Supplementary material for: Toxins from scratch? Diverse, multimodal gene origins in the predatory robber fly Dasypogon diadema indicate a dynamic venom evolution in dipteran insects
Source: Gigascience. 2019 Jul 9;8(7):giz081. doi: 10.1093/gigascience/giz081 (PMC6615979; doi:10.1093/gigascience/giz081)
Supplement: giz081_GIGA-D-19-00072_Original_Submission [file giz081_giga-d-19-00072_original_submission.pdf]

GigaScience

Toxins from scratch? - Diverse, multimodal gene origins in predatory robber flies indicate dynamic venom evolution in dipteran insects

--Manuscript Draft--

**Manuscript Number:**	GIGA-D-19-00072
**Full Title:**	Toxins from scratch? - Diverse, multimodal gene origins in predatory robber flies indicate dynamic venom evolution in dipteran insects
**Article Type:**	Research
**Funding Information:**	Deutsche Forschungsgemeinschaft (RE3454/4-1) — Dr Bjoern Marcus von Reumont<br>Paul Scherer Institut, Villigen (20160644) — Dr Bjoern Marcus von Reumont<br>Australian Research Council (AU) (DE160101142) — Dr Eivind Andreas Baste Undheim<br>Australian Research Council (DP160104025) — Dr Eivind Andreas Baste Undheim
**Abstract:**	Venoms and the toxins they contain represent molecular adaptations that have evolved on numerous occasions throughout the animal kingdom. However, the processes that shape venom protein evolution are poorly understood because of the scarcity of whole genome data available for comparative analyses of venomous species.<br>Here, we perform a broad comparative toxicogenomic analysis to gain insight into the genomic mechanisms of venom evolution in robber flies (Asilidae). We first sequenced a high-quality draft genome of the hymenopteran hunting robber fly Dasypogon diadema, analyzed its venom by a combined proteotranscriptomic approach, and compared our results to recently described robber fly venoms to assess the general composition and major components of asilid venom. We then applied a comparative genomics approach, based on one additional asilid genome, ten high-quality dipteran genomes, and two lepidopteran outgroup-genomes, to reveal the evolutionary mechanisms and origins of identified venom proteins in robber flies.<br>While for 15 out of 30 predominant venom protein homologs were identified in the non-asilid genomes, the remaining 15 highly expressed venom proteins appear to be unique to robber flies. Our results reveal that the venom of D. diadema likely evolves in a multimodal fashion comprising 1) neofunctionalization after gene duplication, 2) expression-dependent co-option of proteins and 3) asilid lineage-specific orphan genes with enigmatic origin. The role of such orphan genes is currently being disputed in evolutionary genomics, but has not yet discussed in the context of toxin evolution. Our results display an unexpected dynamic venom evolution in asilid insects, which contrasts the findings of the only other insect toxicogenomic evolutionary analysis, parasitoid wasps (Hymenoptera), where toxin evolution is dominated by single gene co-option. These findings underpin the significance of further genomic studies to cover more neglected lineages of venomous taxa and to understand the importance of orphan genes as possible drivers for venom evolution.
**Corresponding Author:**	Bjoern Marcus von Reumont<br>University of Gießen<br>GERMANY
**Corresponding Author Secondary Information:**	
**Corresponding Author's Institution:**	University of Gießen
**Corresponding Author's Secondary Institution:**	
**First Author:**	Stephan Holger Drukewitz
**First Author Secondary Information:**	
**Order of Authors:**	Stephan Holger Drukewitz<br>Eivind Andreas Baste Undheim

Powered by Editorial Manager® and ProduXion Manager® from Aries Systems Corporation

	Lukas Bokelmann
	Bjoern Marcus von Reumont
**Order of Authors Secondary Information:**	
**Additional Information:**	
**Question**	**Response**
Are you submitting this manuscript to a special series or article collection?	No
**Experimental design and statistics**<br><br>Full details of the experimental design and statistical methods used should be given in the Methods section, as detailed in our Minimum Standards Reporting Checklist. Information essential to interpreting the data presented should be made available in the figure legends.<br><br>Have you included all the information requested in your manuscript?	Yes
**Resources**<br><br>A description of all resources used, including antibodies, cell lines, animals and software tools, with enough information to allow them to be uniquely identified, should be included in the Methods section. Authors are strongly encouraged to cite Research Resource Identifiers (RRIDs) for antibodies, model organisms and tools, where possible.<br><br>Have you included the information requested as detailed in our Minimum Standards Reporting Checklist?	Yes
**Availability of data and materials**<br><br>All datasets and code on which the conclusions of the paper rely must be either included in your submission or deposited in publicly available repositories (where available and ethically appropriate), referencing such data using	Yes

a unique identifier in the references and in the “Availability of Data and Materials” section of your manuscript.<br><br>Have you have met the above requirement as detailed in our Minimum Standards Reporting Checklist?	

Powered by Editorial Manager® and ProduXion Manager® from Aries Systems Corporation

Manuscript
Click here to view linked References
Click here to access/download;Manuscript;Drukewitz_etal_GigaScience_Re

Submission type: Article

Toxins from scratch? - Diverse, multimodal gene origins in the predatory robber fly *Dasypogon diadema* indicate a dynamic venom evolution in dipteran insects

Stephan Holger Drukewitz[1,7#], Lukas Bokelmann[2], Eivind A B Undheim[3,4], Björn M von Reumont[5,6,7#]

[1] University of Leipzig, Institute for Biology, Talstr. 33, 04103 Leipzig, Germany, e-mail address

[2] Max Planck Institute for Evolutionary Anthropology, Evolutionary Genetics Department, Deutscher Platz 6, D-04103 Leipzig

[3] Centre for Advanced Imaging, The University of Queensland, St. Lucia, QLD 4072, Australia

[4] Centre for Ecology and Evolutionary Synthesis, Department of Biosciences, University of Oslo, PO Box 1066 Blindern, 0316 Oslo, Norway

[5] LOEWE Centre for Translational Biodiversity Genomics (LOEWE-TBG), Senckenberganlage 25, 60325 Frankfurt, Germany

[6] Justus Liebig University, Institute for Insect Biotechnology, Heinrich Buff Ring 58, 35394, Gießen, Germany, bjoern.von-reumont@agrar.uni-giessen.de

[7] Fraunhofer Institute for Molecular Biology and Applied Ecology, Project group Bioressources, Animal Venomics, Winchesterstrasse 2, 35392, Gießen, Germany

[#] Corresponding authors

SHD steph-druk@web.de; stephan.drukewitz@uni-leipzig.de

LB lukas_bokelmann@eva.mpg.de

EU e.undheim@uq.edu.au

BMvR bmvr@arcor.de

Abstract

Venoms and the toxins they contain represent molecular adaptations that have evolved on numerous occasions throughout the animal kingdom. However, the processes that shape venom protein evolution are poorly understood because of the scarcity of whole genome data available for comparative analyses of venomous species.

Here, we perform a broad comparative toxicogenomic analysis to gain insight into the genomic mechanisms of venom evolution in robber flies (Asilidae). We first sequenced a high-quality draft genome of the hymenopteran hunting robber fly *Dasypogon diadema*, analyzed its venom by a combined proteotranscriptomic approach, and compared our results to recently described robber fly venoms to assess the general composition and major components of asilid venom. We then applied a comparative genomics approach, based on one additional asilid genome, ten high-quality dipteran genomes, and two lepidopteran outgroup-genomes, to reveal the evolutionary mechanisms and origins of identified venom proteins in robber flies.

While for 15 out of 30 predominant venom protein homologs were identified in the non-asilid genomes, the remaining 15 highly expressed venom proteins appear to be unique to robber flies. Our results reveal that the venom of *D. diadema* likely evolves in a multimodal fashion comprising 1) neofunctionalization after gene duplication, 2) expression-dependent co-option of proteins and 3) asilid lineage-specific orphan genes with enigmatic origin. The role of such orphan genes is currently being disputed in evolutionary genomics, but has not yet discussed in the context of toxin evolution. Our results display an unexpected dynamic venom evolution in asilid

insects, which contrasts the findings of the only other insect toxicogenomic evolutionary analysis, parasitoid wasps (Hymenoptera), where toxin evolution is dominated by single gene co-option. These findings underpin the significance of further genomic studies to cover more neglected lineages of venomous taxa and to understand the importance of orphan genes as possible drivers for venom evolution.

Introduction

The predominant scenario for the evolution of a new gene function presumes that a gene duplication is followed by a neo- or sub-functionalization of one of the copies, which results in a novel gene function [1,2]. To differentiate mechanisms of gene origin, a larger taxon sampling and good quality of utilized whole genome data are mandatory. This objective is now more achievable because of the fast development in next generation sequencing technology. However, whole genome data for comparative analyses are still sparse in evolutionary venomics (Supp. Tab. 1), and as a consequence, the relative importance of the underlying mechanisms in the evolution of venom proteins and peptides remain to be addressed in more detail.

Venoms have evolved across a wide range of animal lineages as important evolutionary traits that are used for predation, defense or competition [3–6]. They are cocktails of bioactive molecules that are usually composed mainly of peptides and proteins, collectively referred to as “toxins” that often exhibit a variety of pharmacological properties linked to their toxicity. These venom proteins and peptides have evolved new toxic functions from non-toxic ancestral versions, and they are thus ideal candidates to test classical hypotheses on the evolution of new gene functions.

However, only a few comparative studies based on whole genome data have explored the different mechanisms that instigate the origin of toxin genes. In general, toxin evolution by gene duplication represents a widely accepted hypothesis and receives support as a major mechanism of toxin origin from genomic analyses of the king cobra (*Ophiophagus hannah*), the Chinese scorpion (*Mesobuthus martensii)* and the Brazilian white-knee tarantula (*Acanthoscurria geniculata)* [7–9]. In contrast, analyses of the genomes of the platypus (*Ornithorhynchus anatinus)* and parasitic wasps (*Nasonia vitripennis, Trichomalopsis sarcophagae)* found that in these lineages, co-option of single copy genes reflects the dominating process that shapes toxin evolution [10,11]. Nevertheless, the available genomes of venomous taxa often reflect improper sampling densities of the respective lineages (Supp. Tab. 1). As a consequence, there is a need for comparative approaches, which add more genome data to clades of interest and suitable outgroups, to provide a better understanding of general processes in toxin evolution.

In this study, we examine the processes that drive toxin evolution in robber flies (Asilidae, Diptera), which is one of the largest extant fly groups and includes over 7000 species [6,12]. Asilids are also the only known clade within dipteran insects in which both genders use venom for an adult predatory lifestyle [6,12]. We first characterized the venom system of male and female specimens of *Dasypogon diadema* using a combination of functional morphology, venom gland transcriptomics, and venom proteomics. *D. diadema* is of particular interest because it specializes in hunting hymenopterans, which possess venom that can be used in defense and thus represent potentially dangerous prey [13,14]. We also utilized transcriptome and proteome data from the venom of two additional European asilids (*Eutolmus rufibarbis* and *Machimus arthriticus*) to determine major venom components in robber

flies [12], and compared our results with a third, recently published study of the Australian giant robber fly (*Dolopus genitalis*) [15].

The mechanisms by which the identified venom proteins evolved in *D. diadema* were subsequently inferred by performing an extensive comparative genomics analysis. To reveal the evolutionary origin of asilid venom proteins, we sequenced, assembled and annotated a high-quality draft genome of *D. diadema,* and co-annotated a recently published genome of the asilid *Proctacanthus coquiletti* [16]. We then compared these to publicly available high-quality genomes of 12 species of fly (dipteran) and butterfly (lepidopteran) model organisms, 10 of which were dipterans. Our results reveal a complex, multimodal pattern for the origin of venom proteins, and that the venom of *D. diadema* evolved dynamically through mechanisms that include both gene duplication and single gene co-option. The venom proteins partly originate from genes with ancestral variants already present in the protein-coding genome of the last common ancestor (LCA) of Diptera and Lepidoptera. Other putative toxins are lineage-specific to robber flies and show no detectable homologs outside the asilid genomes. Our results are based on the currently largest data set of comparative genomics in evolutionary venomics, and demonstrate the promises of, but also the need for, comparative genomics to understand venom evolution in a broader context.

Results

The venom system of Dasypogon diadema

To compare the venom delivery system of *D. diadema* with previously described asilid species, we examined the morphology of its venom apparatus by performing synchrotron-based micro-computer tomography reconstructions of both a male and a

female specimen. We found no differences between the compared male and female specimen of *D. diadema;* however in order to discount sexual dimorphism in asilid venom systems, this result should be combined with a larger sampling size per sex for definite conclusions. The venom apparatus of *D. diadema* appears generally similar to the previously described structures of *E. rufibarbis* [12], with the exception that the venom apparatus of *D. diadema* features more complex and elongated, sub-structured thoracic venom glands (Fig. 1).

Complementing our morphological analysis, the venom composition of *D. diadema* was investigated by applying a combination of venom gland, proboscis and body tissue transcriptomics and a proteomic analysis of venom gland extracts from both sexes. Apart from a more complex morphology, the venom cocktail of *D. diadema* showed a number of differences compared to the described venom of *E. rufibarbis* and *M. arthriticus* [12]. The most striking disparity is that the venom of *D. diadema* contained chitinase-like proteins and proteins that belong to the CAP-superfamily, which were absent in the venoms of *E. rufibarbis* and *M. arthriticus* (Fig. 2). The expression level of transcripts coding for chitinase-like proteins were ranked third (female) and fourth (male) among all identified venom proteins (male: TPM 4.16 %; female: TPM 3.85 %, percentage of the summed up TPM value of all identified venom proteins), while CAP-like proteins were expressed on a comparably low level in both sexes (male: TPM 1.34 %; female: TPM 1.23 %) (Fig. 2). We also identified five families of novel venom proteins among the 30 predominant putative toxins, which we named asilidin$_{11–15}$, according to existing robber fly toxin nomenclature [12,17](Fig. 2, Fig. 4, Supp. Tab.2 and Supplementary File 4). Lastly, we identified peptidase S1 in the venom of *D. diadema*, which is also abundant in the venoms of *E. rufibarbis* and *M. arthriticus*.

While we observed differences between species, there were also a number of families with similar expression levels across the examined species, which we define as major venom components of asilids. One such component is the previously described family asilidin$_1$ (*E. rufibarbis*: 2.4 %, *M. arthritcus*: 2.13 %, female – *D. diadema*: 1.91 %, male – *D. diadema*: 2.18 %) [12]: its putative cysteine inhibitor knot peptides (ICKs) were shown to have neurotoxic effects on the European honey bee (*Apis mellifera)* [12]. As for *E. rufibarbis* and *M. arthriticus*, we also identified members of the asilidin$_5$ family and MBF2-domain-like proteins in the venom of *D. diadema*. However, the two most dominantly expressed venom gland protein families for all species are asilidin$_2$ and asilidin$_3$, which account for 75 % (*M. arthriticus), 75 %* (male *D. diadema),* 83 % (female *D. diadema)* and 86 % (*E. rufibarbis)* of the toxin-assigned TPM values (Fig. 2).

Genome data quality and completeness

To assess the evolutionary origin of the venom proteins of *D. diadema*, we combined the protein-coding genome of high-quality genomes from Diptera and Lepidoptera with our venomic data from female and male specimen of *D. diadema* (Tab.1; Supp. Tab.2) [18,19]. We also used our venomic data to re-annotate the first high-quality robber fly genome, of *P. coquilletti* [16], and to annotate the *D. diadema* genome sequenced and assembled in the present study (Tab.1; Supp. Tab.3, accession numbers of SRA and BioSample entries for transcriptome and genome data are linked to the BioProject PRJNA361480, see also section data availability). Both robber fly genome annotations were refined by including all transcriptomic and proteomic data of asilid venom glands during the annotation.

Gene sets of dipterans and lepidopterans obtained from ENSEMBL scored a 68.9 % to 99.7 % completeness when analyzed with BUSCO (Tab.1) [20,21]. The presented sets of protein-coding genes of the robber flies *P. coquilletti* and *D. diadema* match this range, scoring 96.7 % and 91.1 % completeness, revealing high quality annotations and assembly completeness (Tab1).

Table 1: Overview of all analyzed genomes and their gene-completeness. To infer the quality of the annotation, a BUSCO analysis was performed using the transcriptome mode and the holometabolous dataset. * genome was sequenced and annotated for this study; ** genome from Dikow et.al. 2017 was reannotated; *** protein dataset from ENSEMBL. The order of the species iin this table matches the species order in the cladogram in Figure 3a.

**Order**	**Species**	**Number of analyzed CDS's**	**BUSCO completeness**	
Lepidoptera	*Bombyx mori*	14,623	C:84.5 %	***
	Danaus plexippus	15,128	C:94.8 %	***
Diptera	*Culex quinquefasciatus*	19,032	C:89.9 %	***
	Aedes aegypti	17,158	C:95.5 %	***
	Anopheles gambiae	14,916	C:98.6 %	***
	Anopheles darlingi	10,519	C:90.1 %	***
	Maytiola destructor	22,410	C:86.7 %	***
	Dasypogon diadema	**15,480**	**C:91.1 %**	*****
	Proctacanthus coquiletti	10,942	C:96.7 %	**
	Drosophila grimshawi	19,429	C:99.4 %	***
	Drosophila melanogaster	30,429	C:99.7 %	***
	Drosophila simulans	24,119	C:99.2 %	***
	Teleopsis dalmani	16,570	C:68.9 %	***
	Lucilia cuprina	14,452	C:91.7 %	***

Assessing ancestral gene variants

The protein-coding genomes of *D. diadema* and *P. coquilletti*, ten non-robber fly dipterans, and two lepidopterans were compared and sorted using the Orthofinder pipeline (Tab.1) [18]. Orthofinder performs a BlastP similarity search followed by normalization for sequence length, creation of an orthogroups graph, and MCL-clustering to sort the genes according to their likeliest homology relationships. The created orthogroups comprise protein-coding genes that originated from a single gene in the LCA of all analyzed species or lineage-specific genes in a certain clade. An orthogroup can comprise several or only parts of a single gene family, which might change with the analyzed taxa and the depth of the considered evolutionary splits. Genes without homologs in any of the included genomes cannot be assigned to orthogroups.

The final annotation of the *D. diadema* genome consists of 15,480 protein-coding genes, of which 13,981 genes were sorted into 8,878 orthogroups. The remaining 1499 protein coding genes did not match any of the assigned orthogroups (Fig. 3a, Supp. File. 2). In our analysis *D. diadema* served as the focal organism, the origin of the protein coding genes was inferred from their first-time emergence. For instance, genes of *D. diadema* with homologs in the lepidopterans *Bombyx mori* or *Danaus plexippus* or both were assigned to originate in the LCA of Diptera and Lepidoptera, or earlier. Following this concept, orthogroups were sorted to the considered phylogenetic splits (Fig. 3a).

The split between the Diptera and Lepidoptera lineages is the oldest one considered in our analyses. These two clades share 84 % (7,471) of the orthogroups assigned to

D. diadema (Fig. 3) [22], meaning the ancestral versions of these protein-coding genes already existed in the LCA of the dipteran and lepidopteran clade. Of the remaining orthogroups, 877 are unique for the clade of Diptera, 158 are unique for the split between the gall midge *Mayetiola destructor* and the brachyceran clade, 246 are unique for Brachycera, and 110 orthogroups are shared only between the two robber flies (Fig. 3a). Sixteen orthogroups are constituted of protein-coding genes found exclusively in *D. diadema* (Fig. 3a).

The venom gland proteins identified via proteomics were sorted to their associated orthogroups. We then predicted if the non-toxic ancestral version of a putative toxin was already present in the protein-coding genome of the LCA of the compared species, or if the protein is a unique novelty for a certain clade. 109 orthogroups, which were already present in the LCA of Lepidoptera and Diptera, are associated with at least one venom protein of the female and male *D. diadema*. Three orthogroups with venom proteins were unique to each of Diptera and Brachycera, while eight orthogroups with putative toxins were shared only between the two robber fly genomes (Fig. 3a). The majority of proteins identified in the venom gland can be assigned to protein-coding genes present in the orthogroups shared between the Lepidoptera and the Diptera clade. The transcripts of venom proteins assigned to orthogroups, which arise on node 2, node 3 or node 4 are expressed on a low level in the venom glands of both sexes. Putative toxin transcripts of node 1, node 5 and the ones assigned to no orthogroup are expressed on a high level in the venom glands of both sexes (Fig. 3b, 3c, Supp. Fig. 3, Supp. Fig. 4).

Evolutionary pattern of the predominant venom proteins

To prevent an over-interpretation of the data, the process of venom evolution in *D. diadema* based on whole genome data was analyzed by using a stricter threshold and focusing exclusively on the dominant putative toxin transcripts. For this purpose, we included only putative toxin transcripts that were detected via proteomics, display an expression level in the venom gland of at least 500 TPM, and show a 4-fold higher expression level in the venom gland compared to the respective body tissue. Two independent tools (Segemehl and Salmon) were applied to perform the RNA quantification and to test the robustness of the results [23,24]. Both quantification approaches using identical thresholds reveal similar results. All 28 putative toxin transcripts identified via Segemehl were also identified with Salmon. Salmon, however, reported two further transcripts that still met the threshold. Further downstream analyses were based on the results from the quantification with Salmon (Fig. 3b, 3c, Supp. Fig. 3, Supp. Fig. 4).

For three of the putative toxin transcripts (two variants of asilidin$_3$ and one variant of asilidin$_1$) no orthogroup was assigned, suggesting these genes are unique for *D. diadema* (Fig. 3, Fig. 4, Supp. File 3, Supp. Tab. 6). The 27 putative toxin transcripts that are assigned to orthogroups distribute into 20 different orthogroups (Supp. Tab. 6, Supp. File 3). While 11 of these orthogroups are shared between the lepidopteran and dipteran clade, two orthogroups are unique for the dipteran clade, one for the brachycerans and six are shared only between the asilids. In general, 22 putative toxins can be categorized as multi-copy genes (Fig. 4). They are distributed between 15 different orthogroups, each comprised of at least two protein-coding genes of *D. diadema.* Five of these groups comprise two or more of the 30 predominant putative toxins. In two orthogroups (OG009368, OG0011154), all members are putative toxins and are present in the venom gland (Supp. Tab.6). For 10 orthogroups, only one

member is a putative toxin present in the venom gland while the others are not. The newly identified putative toxins asilidin$_{12}$, asilidin$_{13}$ and asilidin$_{14}$ are all single copy genes, while the asilidin$_{11}$ and asilidin$_{15}$ are categorized as multi-copy genes (Supp. Tab.6).

Asilidin$_2$ variants are distributed across four different orthogroups — three of these are shared only between *D. diadema* and *P. coquilletti* while the remaining one is shared between the Lepidoptera and Diptera (Fig. 4). A similar picture is revealed in larger protein families like PS1 and chitinase-like, for which distinct versions of putative toxin from different orthogroups were identified (Fig. 4, Supp. Tab.6).

Transposable elements

Transposable elements were identified in 11 of the 30 predominant toxins of *D. diadema*, including asilidin$_2$, peptidase S1, chitinase, MBF2-domain, asilidin$_6$, asilidin$_9$, asilidin$_{11}$, asilidin$_{12}$, asilidin$_{13}$ and asilidin$_{15}$ (Supp. Tab. 7). In the dominant component asilidin$_2$, two gene variants harbor transposable elements in the intron sequence. In contrast, all gene variants classified as asilidin$_3$, the second most highly expressed venom component, do not feature transposable elements. The majority of the transposable elements resemble retrotransposons classified as long terminal repeat retrotransposons (LTRs) of currently unknown groups. Other identified elements are retrotransposons classified as long interspersed nuclear elements (LINEs) and DNA-transposons classified as Mariner-like elements (Supp. Tab. 7).

Discussion

General aspects on the venom biology and composition

Dasypogon diadema is a widely distributed robber fly that is known to hunt honey bees (*Apis mellifera)* and other hymenopterans (Poulton 1907; Geller-Grimm 1995). To overpower such dangerous prey, venom with neurotoxic components for rapid paralysis is advantageous. Trophic specialization has also been shown to affect venom composition and even venom apparatus morphology in other predatory venomous lineages, such as snakes [25,26] and spiders [27]. We therefore expected the venom composition of *D. diadema* to contain substantial differences compared to the previously studied, more generalist species *E. rufibarbis* and *M. arthriticus*. Indeed, their venoms differ in some aspects, such as the presence of chitinase and CAP proteins in *D. diadema*, which were not detected in the venoms of *E. rufibarbis* and *M. arthriticus*. Similar to *D. diadema,* the venom composition of the Australian robber fly *Dolopus genitalis* (published during the review process of this manuscript) also appears to contain a larger fraction of enzymatic proteins than *E. rufibarbis* and *M. arthriticus* [15]. *D. genitalis* venom also contained all asilidin families and major venom components that we discuss here [15]. Lastly, Asilidin$_2$ is an especially highly expressed component in all asilids, including *D. genitalis*. The observed slight gender-specific variation of the venom composition in our pooled samples of male and female individuals might be explained by the known differing ecology of males and females. However, this hypothesis is speculative and requires further testing with additional replicates.

In general the venom of *D. diadema* shares the major components with *E. rufibarbis,* and *M. arthriticus.* Additionally, the most dominant protein families in the venoms of all three species are asilidin$_2$ and asilidin$_3$, and all species also express asilidin$_1$ transcripts (Fig. 2). The phylogenetic distance between *E. rufibarbis, M. arthriticus* (members of the larger subfamily Asilinae) compared to *D. diadema* (representative

of the subfamily Dasypogoninae) [16,28] suggests that these three protein classes resemble lineage-specific toxin arsenal of robber flies, a conclusion that is corroborated by the study of Walker and colleagues [15]. However, for deeper, more detailed insights into the variability of venom composition in asilids, a denser taxon sampling including more individual replicates is required.

The evolution of the neurotoxic component asilidin$_1$

Asilidin$_1$ peptides resemble a cystine inhibitor knot-like fold (ICK), and one representative, U-asilidin$_1$-Mar1a, was shown to induce neurotoxic effects on the European honey bee (*Apis mellifera)* [12]. Facilitating a fast and efficient paralysis of prey asilidin1 probably embody a biologically important venom component in robber fly venom. ICK peptides have been convergently recruited as neurotoxic venom components in a range of venomous lineages, including scorpions, spiders, assassin bugs, cone snails, and possibly also remipede crustaceans [29–39]. The identification of ancestral versions of short neurotoxins, such as ICK peptides, that feature a conserved cysteine scaffold with variable positions between the cysteines remains a challenge [35]. Indeed, while our complementary proteomic and transcriptomic analyses of the venom gland proteins of *D. diadema* revealed three different asilidin$_1$ variants, only one protein-coding gene was detected at the genome level. This suggests that asilidin$_1$ is not a member of a gene family with several duplicates but represents a single-copy gene. Differences in the coding sequences thus likely reflect allelic variation in specimens that had to be pooled for proteome and transcriptome analyses to achieve sufficient tissue quantities. This finding highlights the possible bias of predicting toxin diversity in data from pooled samples.

General *patterns of venom protein evolution*

The evolutionary origin of the major venom proteins in *D. diadema* can be classified into two major categories. The first category comprises variants of both single and multi-copy genes with ancient origin. These robber fly toxins have homologous genes in the lepidopterans or non-asilid dipterans, and originate from ancestral protein versions, which occur in the LCA of asilids and the respective clade.

Four single copy genes of the protein families asilidin$_{12}$, asilidin$_{13}$, asilidin$_{14}$ and chitinase with homologs outside the asilid clade provide examples of venom protein evolution without gene duplication These genes (13,3% of the predominant venom proteins) most likely feature an expression-dependent single gene co-option-type functional recruitment. Under this scenario, an up-regulation of expression in the venom gland tissue and the injection of the otherwise physiological protein as a venom component might lead to a toxic effect in the prey species. In contrast, putative toxins of the protein families asilidin$_{2}$, asilidin$_{9}$, CAP, chitinase, Peptidase S1 and MBF2-domain-like proteins, are present as multi-copy genes. The revealed pattern of one or more duplication events in the history of these genes, supports the widely proposed hypothesis of toxin evolution by gene duplication [3,4,40].

The second category of venom proteins includes putative toxins without homologs outside the asilid lineage. Multi-copy genes dominate this category (asilidin$_{2}$, Peptidase S1), although single copy genes are also present (asilidin$_{6}$). Particularly asilidin$_{2}$ shows a pattern of intense gene duplication, and several transcripts in this family from different orthogroups are secreted in the venom glands. These single and multi-copy genes are lineage-specific and their ancestry is enigmatic. Intriguingly, we identified transposable elements in 11 venom proteins, including variants of the highly expressed asilidin$_{2}$. Two thirds of the venom proteins do not show any

presence of transposable elements. We can only speculate here that the evolution of single toxins might be influenced by transposable elements, and that this might be an explanation for the diversity of asilin$_2$ variants. However, to provide a profound analysis on the influence of transposable elements on the evolution of venom proteins, the analysis design needs to be adapted and whole genome data and venom protein data of more species needs to be included.

Conclusion

The insects include several venomous lineages and comprise the greatest number of venomous species within the animal kingdom [4]. For many of these, the venom compositions and putative toxins remain unknown [6]. Besides hymenopteran and heteropteran taxa, insects also harbor predatory and venomous asilid dipterans. Despite some differences between studied species, our results suggest that the major components of asilid venom constitute new putative toxins that are likely to be restricted to asilids. These include the asilidin$_1$ family, which contains the recently described neurotoxic component U-asilidin$_1$-Mar1a, and has been identified in all four studied asilid venoms, including *D. diadema* [12,15].

The present study includes the currently most comprehensive set of genome species to assess the evolution of venom proteins in *D. diadema* as a representative in the previously uncovered dipteran lineage of robber flies. Our analysis is further strengthened by the implementation of gene-sets from model organisms and closely related species, maximizing our ability to detect toxin homologues and identify the processes that underlie their evolution (Tab.1). This approach revealed that the processes, which contribute to the evolution of toxins in *D. diadema* venom, are

multimodal, and include 1) expression-depending co-option of housekeeping genes, 2) neofunctionalization after gene duplication events, and 3) highly expressed lineage-specific orphan genes. Intriguingly, several of these lineage-specific genes of venom proteins remain of enigmatic origin. The role of these orphan genes as possible drivers in venom evolution represents an intriguing topic for future studies. Our findings highlight the value of studying neglected venomous lineages to improve our understanding of the evolution of venoms and their toxins, and hence the evolutionary mechanisms involved in the evolution of protein function.

Methods

Robber fly collection and sample preservation. Specimens were collected in June 2014 in France at the riverbanks of the river Têt north of Millas in the Département Pyrénées-Orientales (Occitanie) and the vineyards around Brûlat in the Département Var (Provence-Alpes-Cote d'Azur). For transcriptome sequencing samples from body tissue, thoracic gland tissue and proboscis tissue of six males and six females were separately dissected and preserved in RNAlater (Ambion). All dissected individuals were preserved in 94 % Ethanol as voucher specimens. In addition, thoracic glands from seven males and five females were crushed after dissection in 1x PBS buffer with proteinase inhibitor tablets (Roche) for proteomic work. See also Supplementary Fig. 5 for the general workflow. Two individuals for both genders were deposited in Bouin liquid to perform synchrotron based micro-computer tomography.

Venom apparatus. The functional morphology of the venom delivery system in both sexes of *D. diadema* was investigated using synchrotron based micro-computer

tomography. Bouin preserved samples were critical point dried, mounted on a specimen holder and scanned at the Swiss Light Source electron synchrotron accelerator. Morphological structures were segmented in aligned image stacks using ITK-snap v.3.60 [41]. The visualization of the reconstructed three-dimensional model was carried out using Blender v.2.79 [42].

Transcriptomics. Total RNA of thoracic glands, proboscis tissue and body tissue was extracted following the standard protocol for Trizol Reagent by Thermo Fisher. For both sexes the gland and proboscis tissues of six specimens were pooled to guarantee sufficient RNA quantity, while body tissue was extracted from one individual per sex. All six samples for male and female *D. diadema* specimens were prepared for sequencing at the Core Unit DNA Technologies of the University of Leipzig using the Illumina poly-A selection protocol. Sequencing was performed on the Illumina HiScanSQ platform with 100 bp paired end reads (Supp. Tab. 5). All generated data is accessible via the BioProject PRJNA361480, including all BioSample and SRA-entries (See also Supp. Tab. 4). In addition to our own data, all available asiliid transcriptomes were mined in the SRA archive for later genome annotation (Supp. Tab. 4). Own and published raw reads were processed together and first visually inspected in FastQC [43]. Quality filtering and trimming was then applied in trimmomatic v.033 with a minimum length of 60bp and a min phred score of 30 [44]. All pre-processed datasets were finally assembled using Trinity v.2.4 with default settings except a minimum contig length of 138 [45]. The transcript abundance in all *D. diadema* tissue samples was estimated by mapping the trimmed RNA-reads with Segemehl (alignment accuracy 98 %)[24,46] and by comparatively quantifying reads with Salmon (default settings). The TPM (transcripts per million)

values for each coding domain sequence were visualized with a customized Python script and the Seaborn package, see also identification of venom proteins.

Proteomics. The lyophilized venom from the thoracic glands preserved in proteinase inhibitor was dissolved in water prepared for proteomic analysis as described in Drukewitz et al. (2018). Briefly, the samples were desalted by acetone precipitation, proteins reduced with dithiotheitol, alkylated with iodoacetamide, and digested by overnight incubation with trypsin. The digested venom was desalted using a C18 ZipTip (Thermo Fisher, Waltham, MA, USA), dried in a vacuum centrifuge, and dissolved in 0.5 % formic acid before 2 µg of each sample was analyzed by LC-MS/MS on an AB Sciex 5600TripleTOF equipped with a Turbo-V source heated to 550 °C and coupled to a Shimadzu Nexera UHPLC (Kyoto, Japan). The digested venom was fractionated with an Agilent Zorbax stable-bond C18 column (2.1 × 100 mm, 1.8 µm particle size, 300 Å pore size), across a gradient of 1–40 % solvent B (90 % ACN 0.1 % FA) in 0.1 % FA over 60 min, using a flow rate of 180 µL/min. All solvent concentrations are in volume to volume. MS1 survey scans were acquired at 300–1800 m/z over 250 ms, and the 20 most intense ions with a charge of +2 to +5 and an intensity of at least 120 counts/s were selected for MS2. The unit mass precursor ion inclusion window mas ± 0.7 Da, and isotopes within ±2 Da were excluded from MS2, which scans were acquired at 80–1400 m/z over 100 ms and optimized for high resolution.

For protein identification, MS/MS spectra were searched against sequence lists consisting of both the translated venom gland and body transcriptomes of *D. diadema* using ProteinPilot v5.0 (AB Sciex, Framingham, MA, USA). Searches were run as thorough identification searches, specifying urea denaturation, tryptic

digestion and cysteine alkylation by iodoacetamide. Amino acid substitutions and biological modifications were allowed in order to identify potential post-translational modifications and to account for chemical modifications due to experimental artefacts. Decoy-based false discovery rates (FDR) were estimated by ProteinPilot, and for our protein identification we used a protein confidence cut-off corresponding to a local FDR of <0.5 %. Spectra were also manually examined to further eliminate any false positives.

Genome sequencing and assembly. DNA was extracted from 30 mg of muscle tissue of a female specimen of *D. diadema*. The tissue was dissolved in 500 µl lysis buffer (10mM Tris-HCl pH 8, 0.5 % (w/v) SDS, 2.4 mg/ml proteinase K, 1mM EDTA pH 8) for 50 min at 50°C while shaking. Chitinous debris was spun down in a table centrifuge and the DNA was extracted from the supernatant using MinElute silica spin columns (MinElute PCR Purification Kit, Qiagen) according to the manufacturers' specifications. Two aliquots of 3 µg isolated DNA were sheared to 200 bp and 400 bp average length in a Covaris S220 Focused Ultrasonicator (200 bp settings: 10 dc, 5 i, 200 cpb, fs 180 s; 400 bp settings: 10 dc, 4 i, fs 55 s). 100 ng sonicated DNA served as input for library preparation as described in Meyer et al. [47]. Both libraries were double-indexed with two 7 bp unique barcodes and amplified as described in Kircher et al.[48]. Paired end reads were subsequently sequenced with 150 bp on an Illumina MiSeq platform. All raw reads were visually inspected in FastQC [43] and then quality filtered and trimmed applying Trimmomatic v.033 with a minimum length of 70 bp and a min phred score of 30 [44]. An overview of sequenced raw reads and processed transcripts are given in Table 2.

Table 2: Overview of DNA libraries generated for the *Dasypogon diadema* genome assembly. Number of read pairs and fragment size of the libraries used for the genome assembly are shown. The theoretical genome coverage was calculated with a genome size estimate of 450 mb and a read length of 120 nt after processing.

Library Name	Fragment length	Number of sequenced read pairs	theoretical genome coverage
D1130	200 nt	9,119,970	5-fold
D1131	400 nt	167,137,385	89-fold

The genome assembly was performed with MaSuRCA v.3.1.3 [49]. To inspect the quality and to exclude possible contamination Blobtools was applied [50]. The final assembly resulted in an overall assembly size of 450 mb (scaffold > 2kb), with a N50 of 32.6 kb and a GC content of 35.81 %. Assembly size, N50 value and other statistics were assessed with Quast v.4.6 [51]. The final genome size is in line with the prior estimated size via k-mer distribution using jellyfish [52], which resulted in 427 mb (Supp. Fig. 2). The assessment with BUSCO (genome mode, holometabolous core gene set) resulted in 92.4 % completeness and a duplication rate of 2.7 %, which indicates a high quality of the draft genome of *D. diadema* and that the heterozygous areas were adequately assembled [20].

Genome Annotation. Our genome sequence of *D. diadema* was co-annotated *with* the recently published genome of *Proctacanthus coquilletti* using the Maker2 pipeline [16,53]. All *de novo* assembled trancriptome data sets were then utilized to identify splice sites using Exonerate [54] (Supp. Table 3). Additionally, the protein sequences of *Aedes aegypti, Anopheles gambiae, Mayetiola destructor, Lucilia cuprina* and *Drosophila melanogaster* from the ENSEMBL genome database and all insect proteins from the Swissprot database were aligned using BLAST+ v.2.6.0. Successful aligned positions were extracted to train the gene prediction software

Augustus and SNAP [21,55–57]. The resulting Maker2 gene set after four iterative training cycles was finally used for further downstream analyses. The annotation resulted in 10,942 protein-coding genes in the genome of *P. coquilletti* and 15,480 protein-coding genes in the genome of *D. diadema.* The completeness of both gene sets was inferred with BUSCO [20] (transcriptome mode, holometabolous core gene set) and resulted in a completeness of 91,1 % for *D. diadema* and 96,7 % for *P. coquilletti* (Tab. 1).

Identification of transposable elements

Repetitive elements in the genome of *D. diadema* and *P. coquilletti* were identified using RepeatModeler (v. open-1.0.11), the resulting repeat library was provided to RepeatMasker (v. open-4.07) [58,59] to mask repetitive elements prior to the annotation of genes. For *D. diadema* the repeatmasker output was parsed with the “One code to find them all” perl tool [60] using the “strict” option. The resulting overview tables were used to analyze the appearance of transposable elements in the Top 30 dominant toxins (Supp. Tab. 7).

Identification of venom proteins. Putative toxins and venom protein families were identified applying the approach described in Drukewitz et al. (2018). The strategies for transcriptomics were to perform BlastP searches against ToxProt, to run hmm searches using HMMER v.3.1b2 [61] against own venom protein databases, and to characterize highly expressed coding regions. The major difference in the present analysis is that coding domain regions used to identify putative toxins are not derived from *de novo* transcripts but instead based on genome loci that were annotated by

transcriptome and proteome sequences. The annotated protein-coding genes of *D. diadema* were matched with the venom gland proteins identified via proteomics applying a strict threshold (e-value of 1e-40, query coverage of 90 %). This cut-off was employed to reduce false positives while at the same time minimize the number of protein-coding genes that might be missed. The transcript abundance in all *D. diadema* tissue samples was estimated based on the trimmed RNA-reads applying the quantification tool Salmon (default settings) and the read mapper Segemehl (alignment accuracy 98 %) [24,46]. To assess evolutionary processes of putative toxins a rigorous TPM value of 500 and a 4fold higher expression level in the venom gland compared to the respective body tissue was picked to prevent over-interpretation of our data.

Additionally, a second threshold with a lower TPM value (> 1) was applied to allow a comparison of the identified venom proteins to previously published robber fly data [12]. Proteins with a housekeeping function, a low expression level in the venom glands and a high expression level in non-venom gland tissue were not considered as putative toxins and excluded from the analysis.

Venom evolution reconciled by genomics. The ENSEMBL database provides 21 annotated dipteran genomes [21], twelve of these are from *Drosophila* species. For *Drosophila*, only three representative genomes were selected for our analyses (Tab.1). Otherwise all available taxa were included, with two exceptions. The wingless antarctic midge *Belgica antarctica* was excluded because of its extremely derived lifestyle. *Megaselia scalaris* was deselected because of the rather experimental approach that was used to sequence its genome [62,63]. As outgroup taxa the lepidopterans *Bombyx mori* and *Danaus plexippus* were chosen [64,65].

Apart from ENSEMBL we also mined NCBI for relevant dipteran genomes, and consequently re-annotated and included the genome of *Proctacanthus coquilletti* (Supp. Tab. 3) [16].

The protein sets of all analyzed genome species were compared and protein-coding genes assigned to orthogroups with Orthofinder [18]. Depending on the taxon samplings orthogroups can comprise gene families, gene classes or only parts of such classification. The aim of the approach is not to identify such hierarchical classes but to infer the homology of the analyzed protein sets [18,19]. Under the assumption that orthogroups only arise one time but might be lost several times, the origin of novelties and the expansion of protein groups can be analyzed. *D. diadema* was used as the focal species, which means that only the orthogroups present in this species were analyzed further. An orthogroup is considered as present in the LCA of *D. diadema* and a clade when members of the orthogroup were present in the genome of *D. diadema* and in at least one representative of the analyzed clade. Shared orthogroups were counted using the Orthofinder output and a customized python script.

Acknowledgement. BMvR thanks Fritz Geller-Grimm for helpful discussions and information on species biology and localities. Sabrina Simon and students from the University of Wageningen, and Alessandra Dupont further assisted to find and to collect specimens. SHD and BMvR thank in particular Martin Schlegel for his support at the Institute of Biology at the University of Leipzig. BMvR likes to thank especially Matthias Meyer at the Max Planck Institute for Evolutionary Anthropology in Leipzig for the fruitful collaboration, also with his team. Computational analyses were partly performed on the High Performance Computing Cluster EVE at the UFZ Leipzig, and

SHD and BMvR like to thank Christian Krause for his help regarding some analyses setups. BMvR was supported for this work by the German Science Foundation (DFG RE3454/4-1). Beamtime at the Paul Scherer Institute, Villigen, Switzerland was provided to BMvR based on the proposal “Evolution of venoms and venom delivery systems of neglected venomous euarthropod and annelid taxa” (ID 20160644). We acknowledge the Paul Scherer Institute, Villigen, Switzerland for provision of synchrotron radiation beamtime at the TOMCAT beamline X02DA of the SLS and would like to thank Goran Lovric for assistance. SHD is funded by a scholarship (Doktorandenförderplatz) from the University of Leipzig. BMvR and SHD conducted this work within the Animal Venomics working group at the Fraunhofer Institute for Molecular Biology and Applied Ecology, Giessen. This work was supported by the Australian Research Council (DECRA Fellowship grant number DE160101142 and Discovery Project grant number DP160104025 to E.A.B.U.). We acknowledge Alessandra Dupont for commenting and editing of the manuscript.

Author contributions

BMvR and SHD conceived the project and designed the analyses. SHD and BMvR performed specimen collection, dissection, transcriptomic and genomic analyses. EABU conducted the proteomic analyses. LB performed all laboratory work for the genome sequencing. BMvR and SHD wrote the manuscript with input from all authors.

Additional Information

Competing interests: The authors declare no competing interests

Data availability

All transcriptome and genome data is available in NCBI via the Bioproject on robber fly venom evolution, PRJNA361480. Transcriptome raw data of male and female venom gland, body and proboscis tissue are published with the SRA entries: SRR7754486, SRR7754485, SRR5192548, SRR5192547, SRR7754488, SRR7754487. The genome assembly is accessible in GenBank under QYTT00000000, the sequencing raw data is stored in the SRA with the two accession numbers: SRR7878513 and SRR7878512.

1. Nei M, Gu X, Sitnikova T. Evolution by the birth-and-death process in multigene families of the vertebrate immune system. Proc Natl Acad Sci. 1997;94:7799–806.

2. Lynch M. The evolutionary fate and consequences of duplicate Genes. Science. 2002;290:1151–5.

3. Casewell NR, Wüster W, Vonk FJ, Harrison RA, Fry BG. Complex cocktails: The evolutionary novelty of venoms. Trends Ecol. Evol. 2013. p. 219–29.

4. Fry BG, Roelants K, Champagne DE, Scheib H, Tyndall JDA, King GF, et al. The toxicogenomic multiverse: Convergent recruitment of proteins into animal venoms. Annu Rev Genomics Hum Genet. 2009;10:483–511.

5. von Reumont BM. Studying smaller and neglected organisms in modern evolutionary venomics implementing RNASeq (Transcriptomics)—A critical guide. Toxins (Basel). 2018.

6. von Reumont BM, Campbell L, Jenner R. Quo vadis venomics? A roadmap to

neglected enomous Invertebrates. Toxins (Basel). 2014;6:3488–3551.

7. Vonk FJ, Casewell NR, Henkel C V, Heimberg AM, Jansen HJ, McCleary RJR, et al. The king cobra genome reveals dynamic gene evolution and adaptation in the snake venom system. Proc Natl Acad Sci. Proceedings of the National Academy of Sciences; 2013;110:20651–20656.

8. Cao Z, Yu Y, Wu Y, Hao P, Di Z, He Y, et al. The genome of *Mesobuthus martensii* reveals a unique adaptation model of arthropods. Nat Commun. Nature Publishing Group; 2013;4:1–10.

9. Sanggaard KW, Bechsgaard JS, Fang X, Duan J, Dyrlund TF, Gupta V, et al. Spider genomes provide insight into composition and evolution of venom and silk. Nat Commun. 2014;5.

10. Wong ESW, Papenfuss AT, Whittington CM, Warren WC, Belov K. A limited role for gene duplications in the evolution of platypus venom. Mol Biol Evol. Oxford University Press; 2012;29:167–77.

11. Martinson EO, Mrinalini, Kelkar YD, Chang CH, Werren JH. The evolution of venom by co-option of single-copy genes. Curr Biol. 2017;27:2007–2013.e8.

12. Drukewitz SH, Fuhrmann N, Undheim EAB, Blanke A, Giribaldi J, Mary R, et al. A dipteran's novel sucker punch: Evolution of arthropod atypical venom with a neurotoxic component in robber flies (asilidae, diptera). Toxins (Basel). 2018;10.

13. Geller-Grimm F. Autökologische Studien an Raubfliegen (Diptera : Asilidae) auf Binnendünen des Oberrheintalgrabens. 1995;

14. Poulton EB. XVI. Predaceous insects and their prey. Trans R Entomol Soc London. 1907;54:323–410.

15. Walker AA, Dobson J, Jin J, Robinson SD, Herzig V, Vetter I, et al. Buzz kill: Function and proteomic composition of venom from the giant assassin fly *Dolopus genitalis* (Diptera: Asilidae). Toxins (Basel). 2018;10.

16. Dikow RB, Frandsen PB, Turcatel M, Dikow T. Genomic and transcriptomic resources for assassin flies including the complete genome sequence of *Proctacanthus coquilletti* (Insecta: Diptera: Asilidae) and 16 representative transcriptomes. PeerJ. 2017;5:e2951.

17. Undheim EAB, Jones A, Clauser KR, Holland JW, Pineda SS, King GF, et al. Clawing through evolution: Toxin diversification and convergence in the ancient lineage Chilopoda (Centipedes). Mol Biol Evol. 2014;31:2124–48.

18. Emms DM, Kelly S. OrthoFinder: solving fundamental biases in whole genome comparisons dramatically improves orthogroup inference accuracy. Genome Biol. 2015;16.

19. Paps J, Holland PWH. Reconstruction of the ancestral metazoan genome reveals an increase in genomic novelty. Nat Commun. 2018;9.

20. Simão FA, Waterhouse RM, Ioannidis P, Kriventseva E V., Zdobnov EM. BUSCO: Assessing genome assembly and annotation completeness with single-copy orthologs. Bioinformatics. 2015;31:3210–2.

21. Hubbard T, Barker D, Birney E, Cameron G, Chen Y, Clark L, et al. The Ensembl genome database project. Nucleic Acids Res. 2002;30:38–41.

22. Misof B, Liu S, Meusemann K, Peters RS, Donath A, Mayer C, et al. Phylogenomics resolves the timing and pattern of insect evolution. Science. 2014;346:763–7.

23. Otto C, Stadler PF, Hoffmann S. Lacking alignments? The next-generation sequencing mapper segemehl revisited. Bioinformatics. 2014;30:1837–43.

24. Patro R, Duggal G, Love MI, Irizarry RA, Kingsford C. Salmon provides fast and bias-aware quantification of transcript expression. Nat Methods. 2017;14:417–9.

25. Daltry JC, Wüster W, Thorpe RS. Diet and snake venom evolution. Nature. 1996;379:537–40.

26. Li M, Fry BG, Kini RM. Eggs-only diet: Its implications for the toxin profile changes and ecology of the marbled sea snake (*Aipysurus eydouxii*). J Mol Evol. 2005;60:81–9.

27. Pekár S, Bočánek O, Michálek O, Petráková L, Haddad CR, Šedo O, et al. Venom gland size and venom complexity - essential trophic adaptations of venomous predators: a case study using spiders. Mol Ecol. Wiley/Blackwell (10.1111); 2018;

28. Dikow T. A phylogenetic hypothesis for Asilidae based on a total evidence analysis of morphological and DNA sequence data (Insecta: Diptera: Brachycera: Asiloidea). Org Divers Evol. 2009;9:165–88.

29. Corzo G, Adachi-Akahane S, Nagao T, Kusui Y, Nakajima T. Novel peptides from assassin bugs (Hemiptera: Reduviidae): Isolation, chemical and biological characterization. FEBS Lett. 2001;499:256–61.

30. Fletcher JI, Smith R, O'Donoghue SI, Nilges M, Connor M, Howden MEH, et al. The structure of a novel insecticidal neurotoxin, ω-atracotoxin-HV1, from the venom of an Australian funnel web spider. Nat Struct Biol. 1997;4:559–66.

31. von Reumont BM, Undheim E, Jauss R-T, Jenner R. Venomics of remipede crustaceans reveals novel peptide diversity and illuminates the venom's biological

role. Toxins (Basel). 2017;9:234.

32. Tripathy A, Meissner G, Resch W, Le Xu, Valdivia HH. Imperatoxin a induces subconductance states in Ca 2+ release channels (ryanodine receptors) of cardiac and skeletal muscle. J Gen Physiol. 2002;111:679–90.

33. Wang X hong, Smith R, Fletcher JI, Wilson H, Wood CJ, Howden MEH, et al. Structure-function studies of ω-atracotoxin, a potent antagonist of insect voltage-gated calcium channels. Eur J Biochem. 1999;264:488–94.

34. von Reumont BM, Blanke A, Richter S, Alvarez F, Bleidorn C, Jenner RA. The first venomous crustacean revealed by transcriptomics and functional morphology: remipede venom glands express a unique toxin cocktail dominated by enzymes and a neurotoxin. Mol Biol Evol. 2014;31:48–58.

35. Undheim EAB, Mobli M, King GF. Toxin structures as evolutionary tools: Using conserved 3D folds to study the evolution of rapidly evolving peptides. BioEssays. Wiley-Blackwell; 2016;38:539–48.

36. Walker AA, Madio B, Jin J, Undheim EAB, Fry BG, King GF. Melt with this kiss: Paralyzing and liquefying venom of the assassin bug *Pristhesancus plagipennis*. Mol Cell Proteomics. 2017;16:552–66.

37. Mayhew ML, King GF, Jin J, Undheim EAB, Fry BG, Meritt DJ, et al. The assassin bug *Pristhesancus plagipennis* produces two distinct venoms in separate gland lumens. Nat Commun. 2018;9.

38. Pineda SS, Undheim EAB, Rupasinghe DB, Ikonomopoulou MP, King GF. Spider venomics: Implications for drug discovery. Future Med. Chem. 2014. p. 1699–714.

39. Herzig V, King GF. The cystine knot is responsible for the exceptional stability of

the insecticidal spider toxin ω-Hexatoxin-Hv1a. Toxins (Basel). 2015;7:4366–80.

40. Hargreaves AD, Swain MT, Hegarty MJ, Logan DW, Mulley JF. Restriction and recruitment-gene duplication and the origin and evolution of snake venom toxins. Genome Biol Evol. Oxford University Press; 2014;6:2088–95.

41. Yushkevich PA, Piven J, Hazlett HC, Smith RG, Ho S, Gee JC, et al. User-guided 3D active contour segmentation of anatomical structures: Significantly improved efficiency and reliability. Neuroimage. 2006;31:1116–28.

42. Blender Foundation. Blender. [Internet]. Free Open 3D Creat. Softw. 1995. p. blender.org. Available from: blender.org

43. Andrews S. FastQC. A quality control tool for high throughput sequence data. Babraham Bioinformatics Web site. [Internet]. 2015. Available from: https://www.bioinformatics.babraham.ac.uk/projects/fastqc/

44. Bolger AM, Lohse M, Usadel B. Trimmomatic: a flexible trimmer for Illumina sequence data. Bioinformatics. Oxford University Press; 2014;30:2114–20.

45. Raychowdhury R, Gnirke A, Fan L, Yassour M, Regev A, di Palma F, et al. Full-length transcriptome assembly from RNA-Seq data without a reference genome. Nat Biotechnol. 2011;29:644–52.

46. Hoffmann S, Otto C, Kurtz S, Sharma CM, Khaitovich P, Vogel J, et al. Fast mapping of short sequences with mismatches, insertions and deletions using index structures. Searls DBE, editor. PLoS Comput Biol. Public Library of Science (PLoS); 2009;5:e1000502.

47. Meyer M, Kircher M. Illumina sequencing library preparation for highly multiplexed target capture and sequencing. Cold Spring Harb Protoc. 2010;5.

48. Kircher M, Sawyer S, Meyer M. Double indexing overcomes inaccuracies in multiplex sequencing on the Illumina platform. Nucleic Acids Res. 2012;40.

49. Zimin A V., Marçais G, Puiu D, Roberts M, Salzberg SL, Yorke JA. The MaSuRCA genome assembler. Bioinformatics. 2013;29:2669–77.

50. Laetsch DR, Blaxter ML. BlobTools: Interrogation of genome assemblies. F1000Research. 2017;6:1287.

51. Gurevich A, Saveliev V, Vyahhi N, Tesler G. QUAST: Quality assessment tool for genome assemblies. Bioinformatics. 2013;29:1072–5.

52. Marçais G, Kingsford C. A fast, lock-free approach for efficient parallel counting of occurrences of k-mers. Bioinformatics. 2011;27:764–70.

53. Holt C, Yandell M. MAKER2: An annotation pipeline and genome-database management tool for second-generation genome projects. BMC Bioinformatics. 2011;12.

54. Slater GSC, Birney E. Automated generation of heuristics for biological sequence comparison. BMC Bioinformatics. 2005;6:31.

55. Korf I. Gene finding in novel genomes. BMC Bioinformatics. 2004;5.

56. Stanke M, Steinkamp R, Waack S, Morgenstern B. AUGUSTUS: A web server for gene finding in eukaryotes. Nucleic Acids Res. 2004;32.

57. Bairoch A. The SWISS-PROT protein sequence database and its supplement TrEMBL in 2000. Nucleic Acids Res. 2000;28:45–8.

58. Smit A, Hubley R. RepeatModeler [Internet]. Available from: http://www.repeatmasker.org/RepeatModeler/

59. Tarailo-Graovac M, Chen N. Using RepeatMasker to identify repetitive elements in genomic sequences. Curr Protoc Bioinforma. 2009;4:Unit 4.10.

60. Bailly-Bechet M, Haudry A, Lerat E. “One code to find them all”: A perl tool to conveniently parse RepeatMasker output files. Mob DNA. BioMed Central; 2014;5:13.

61. HMMER. HMMER: biosequence analysis using profile hidden Markov models [Internet]. Available from: http://hmmer.org/

62. Kelley JL, Peyton JT, Fiston-Lavier AS, Teets NM, Yee MC, Johnston JS, et al. Compact genome of the Antarctic midge is likely an adaptation to an extreme environment. Nat Commun. 2014;5.

63. Rasmussen DA, Noor MAF. What can you do with 0.1x genome coverage? A case study based on a genome survey of the scuttle fly *Megaselia scalaris* (Phoridae). BMC Genomics. 2009;10.

64. Zhan S, Merlin C, Boore JL, Reppert SM. The monarch butterfly genome yields insights into long-distance migration. Cell. 2011;147:1171–85.

65. Xia Q, Zhou Z, Lu C, Cheng D, Dai F, Li B, et al. A draft sequence for the genome of the domesticated silkworm (*Bombyx mori*). Science. 2004;306:1937–40.

Figure. legends

Fig. 1: The three-dimensionally reconstructed venom delivery system of female and male *Dasypogon diadema*. The general anatomy of *Dasypogon diadema* is similar between both genders and to the structures described for *Eutolmus rufibarbis. A* pair of elongated sac-like glands located in the first and second thoracic segments (right and left glands coloured red and orange, respectively) open separately into ducts (coloured

green), which fuse just before entering the head capsule and continues to the tip of the proboscis. Compared to the glands of *Eutolmus rufibarbis*, the glands of *Dasypogon diadema* are more elongated, featuring a larger volume and sub-compartimentalization. The labial glands (coloured blue) are located in the middle part of the proboscis and open into the lumen between theca and the labium at the tip of the proboscis.

Fig. 2: Relative expression of putative toxin families in *Dasypogon diadema* (male and female), compared to *Eutolmus rufibarbis* and *Machimus arthriticus*. The expression levels of protein families secreted in the venom glands are given in percent. Only sequences with matches from proteomics and a threshold above 1 transcripts per million (TPM) are included. Protein classes with an expression value smaller than 1 % of the depicted TPM are summarized in the category “others”. Color code and percentage for every sample are depicted at the end of every graph.

Fig. 3: **(a) Phylogenetic relationships of the included taxa**. *Dasypogon diadema* was used as the focal species for the analyses of the orthogroups. Boxes on the split show the number of orthogroups shared by *Dasypogon diadema* and the respective clade of the split (*upper number*: Number of shared orthogroups; *middle number*: Number of orthogroups with putative toxins; *lower number:* Number of orthogroups associated with the 30 predominant putative toxins. **(b) Heatmap showing the expression level (TPM) in the three tissues of the putative toxins of both sexes.** The white numbers in the black circle refer to the affiliated orthogroups and splits in 3a (Vg-♂: venom gland male; Vg-♀: venom gland female; Pb-♂: proboscis male; Pb-♀: proboscis female; Bt-♂: body tissue male; Bt-♀: body tissue female). **(c) Summarized expression level (TPM) of the putative toxin transcripts in the venom gland of both genders.** The white numbers in the black circle refer to the affiliated orthogroups and splits in 3a. (number of putative toxins for all nodes: Node 1: 130; Node 2: 3; Node 3: 0; Node 4: 5; Node 5: 18; Node 6: 1; *no orthogroup: 4)

Fig. 4: The evolutionary pattern and the origin of the top 30 putative toxins. The node numbering refers to the nodes in Fig. 3a. Putative toxins present in *Dasypogon diadema* but missing in *Eutolmus rufibarbis* or *Machimus arthriticus* are coloured red. **Single copy genes:** putative toxins with only one copy on the protein-coding genome of *Dasypogon diadema*; **Multi copy genes***: protein-coding genes that belong to orthogroups assembled of at least two protein-coding genes in *Dasypogon diadema.* Only one member of the orthogroup is present in the venom; **Multi copy genes**:** protein-coding genes that belong to orthogroups assembled of at least two protein-coding genes in *Dasypogon diadema.* Two or more members of the same orthogroup are present in the venom.

Figure1

Click here to access/download;Figure;Fi

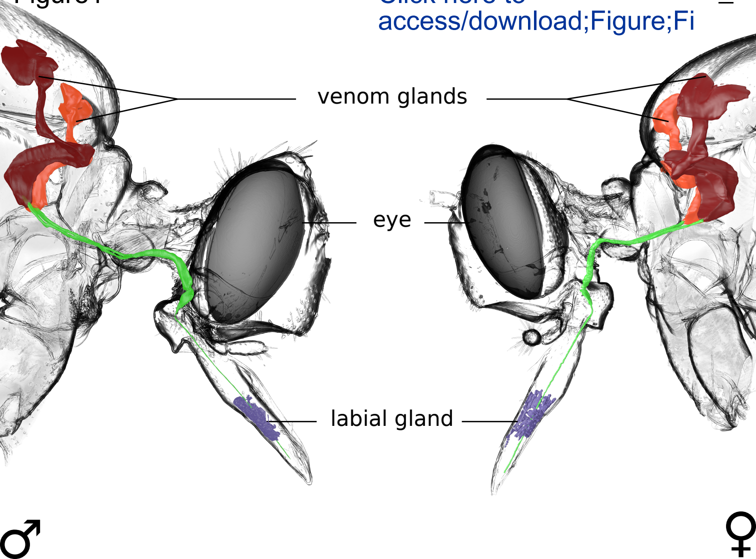

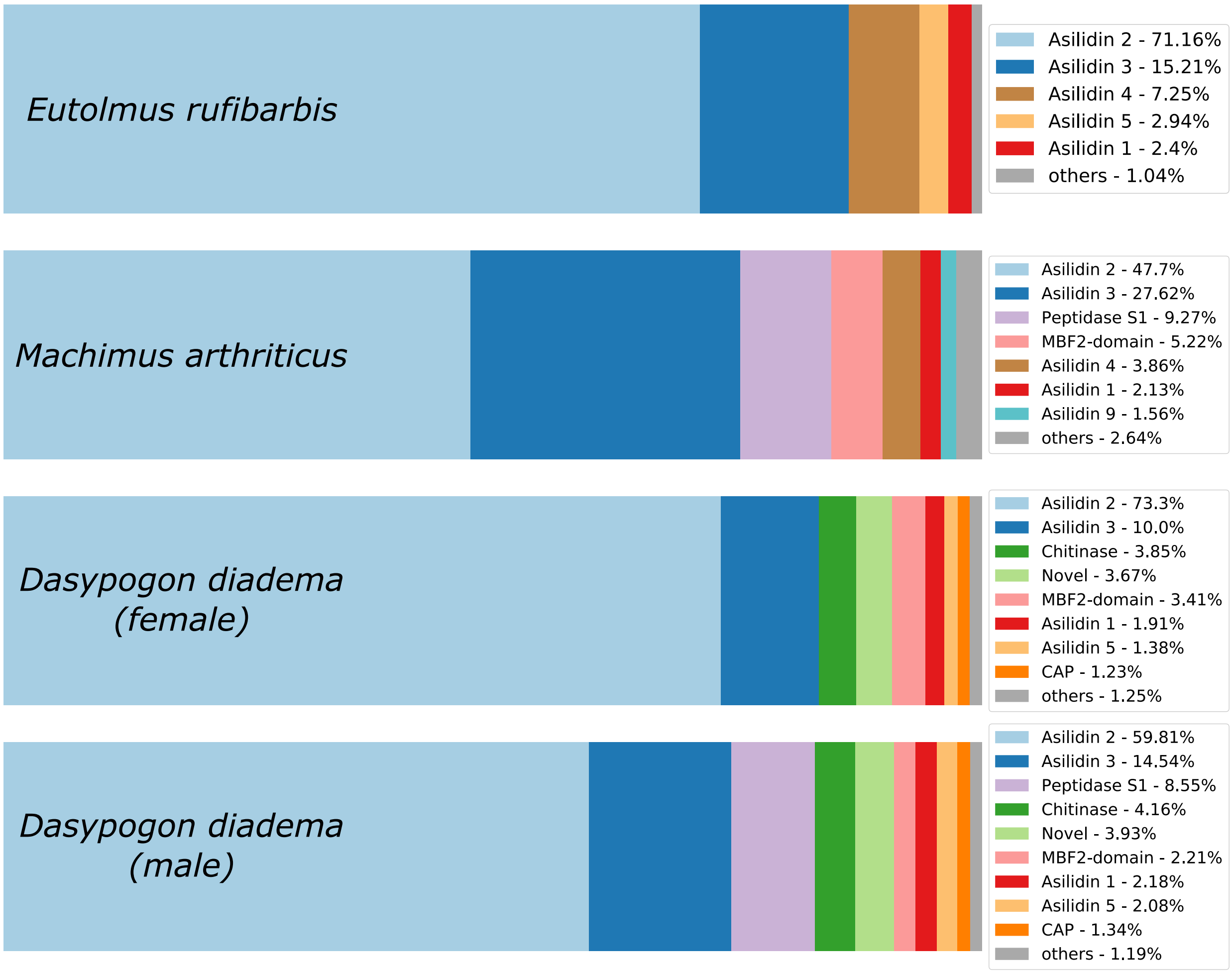
Eutolmus rufibarbis
Asilidin 2 - 71.16%
Asilidin 3 - 15.21%
Asilidin 4 - 7.25%
Asilidin 5 - 2.94%
Asilidin 1 - 2.4%
others - 1.04%
Machimus arthriticus
Asilidin 2 - 47.7%
Asilidin 3 - 27.62%
Peptidase S1 - 9.27%
MBF2-domain - 5.22%
Asilidin 4 - 3.86%
Asilidin 1 - 2.13%
Asilidin 9 - 1.56%
others - 2.64%
Dasypogon diadema (female)
Asilidin 2 - 73.3%
Asilidin 3 - 10.0%
Chitinase - 3.85%
Novel - 3.67%
MBF2-domain - 3.41%
Asilidin 1 - 1.91%
Asilidin 5 - 1.38%
CAP - 1.23%
others - 1.25%
Dasypogon diadema (male)
Asilidin 2 - 59.81%
Asilidin 3 - 14.54%
Peptidase S1 - 8.55%
Chitinase - 4.16%
Novel - 3.93%
MBF2-domain - 2.21%
Asilidin 1 - 2.18%
Asilidin 5 - 2.08%
CAP - 1.34%
others - 1.19%

Figure3

Click here to access/download;Figure;Figure3.pdf

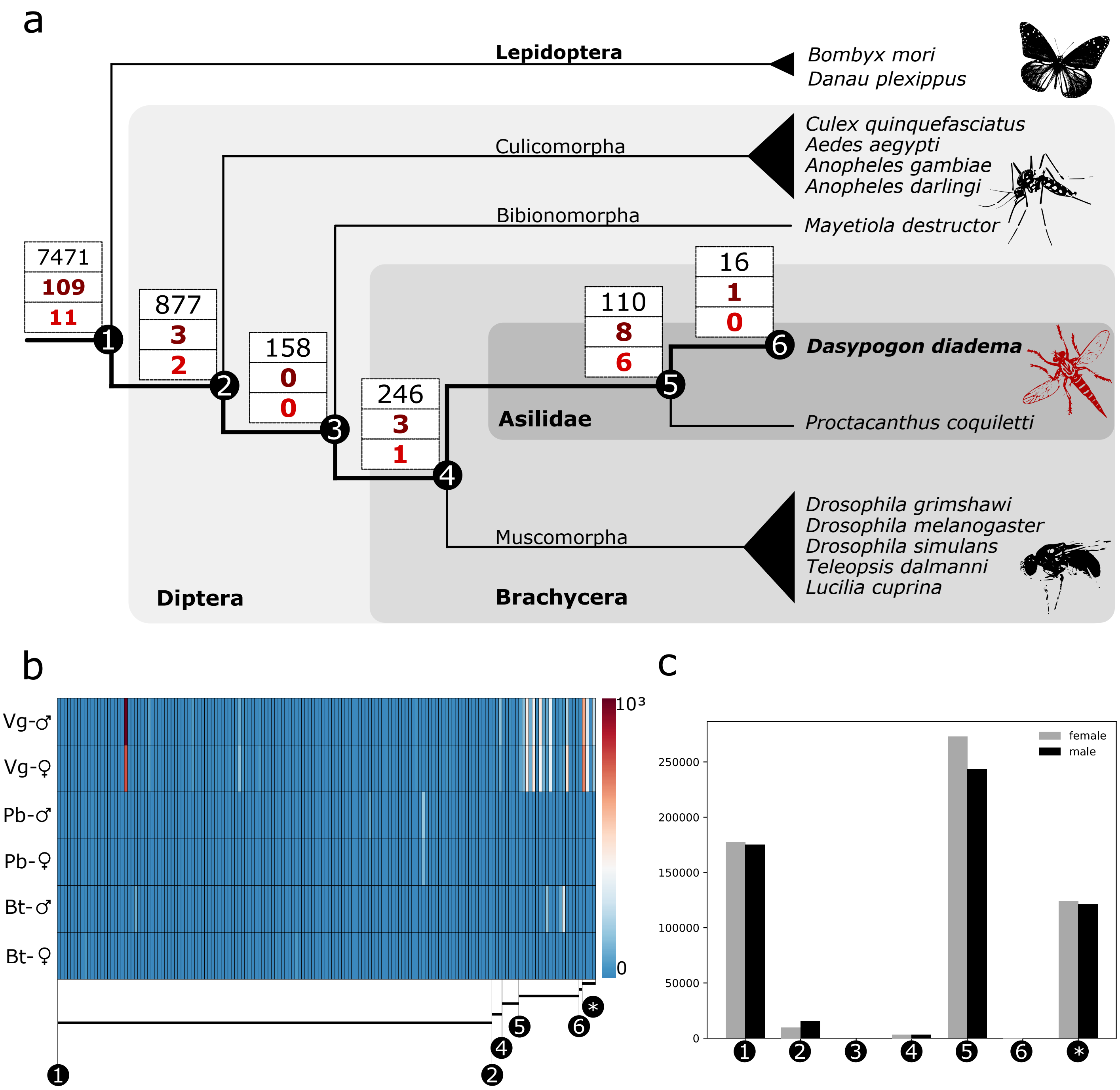

Figure4 Click here to access/download;Figure;Figure4.pdf

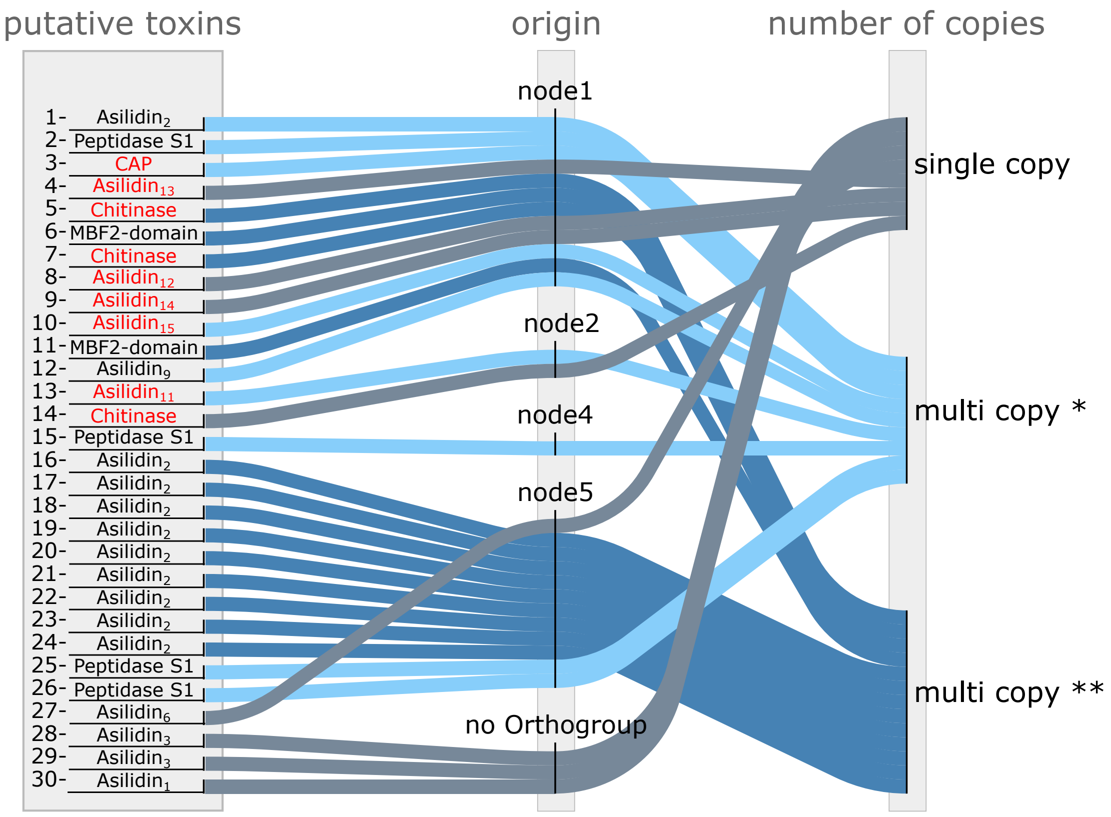

SupplementaryFile_1

Click here to access/download

Supplementary Material

Supplementary_File1_re.pdf

SupplementaryFile2

Click here to access/download
Supplementary Material
Supplementary_File2.xls

Click here to access/download
Supplementary Material
Supplementary_File3.xls

Click here to access/download
Supplementary Material
SupplementaryFile4.txt

Response to reviewers

Click here to access/download

Supplementary Material

Drukewitz_etal_CoverLetter_RevisionReply.pdf
