# Supplementary material for: Toxins from scratch? Diverse, multimodal gene origins in the predatory robber fly Dasypogon diadema indicate a dynamic venom evolution in dipteran insects
Source: Gigascience. 2019 Jul 9;8(7):giz081. doi: 10.1093/gigascience/giz081 (PMC6615979; doi:10.1093/gigascience/giz081)
Supplement: giz081_Supplemental_Files [file giz081_supplemental_files.zip › Supplementary_File1_rev2.pdf]

Toxins from scratch? – Diverse, multimodal gene origins in the predatory robber fly *Dasypogon diadema* indicate a dynamic venom evolution in dipteran insects

Stephan Holger Drukewitz, Lukas Bokelmann, Eivind A B Undheim, Björn M von Reumont

---

Supplementary material.

Supplementary Table 1: Overview of available genome species with analysed venom.

				size in mb	NCBI accession number	N50 in kpb	N's	venome reference
**Arthropoda**	Insecta	Hymenoptera	*Nasonia vitripennis*	292	GCA_000002325.2	921.5	19.6	[1]
			Nasonia giraulti	282	GCA_000004775.1	761.8	37.0	[1]
			Trichomalopsis sarcophagae	219	GCA_002249905.1	25.6	0.5	[1]
			Apis mellifera	225	GCA_003254395.1	13615.1	0.6	[2–4]
			Solenopsis invicta	353	GCA_000188075.1	720.6	11.7	[5.6]
		Diptera	*Dasypogon diadema*	454	QYTT00000000	32.6	0.2	This study
	Arachnida	Araneae	*Acanthoscurria geniculata*	4849	GCA_000661875.1	44.2	40.0	[7]
			Stegodyphus mimosarum	2717	GCA_000611955.2	483.6	1.6	[7]
			Parasteatoda tepidariorum	1439	GCA_000365465.2	4090.7	18.5	[8]
		Scorpiones	*Mesobuthus martensii*	896	GCA_000484575.1	46.8	0.0	[9]
**Cnidaria**			*Nematostella vectensis*	354	GCA_000209225.1	477.8	16.7	[10–12]
			Hydra vulgaris	852	GCA_000004095.1	96.3	7.8	[13.14]
**Vertebrata**			*Ophiophagus hannah*	1477	GCA_000516915.1	269.0	14.4	[15]
			Crotalus viridis viridis	1340	GCA_003400415.2	17989.7	6171.8	[16]
			Ornithorhynchus anatinus	1990	GCA_002966995.1	18718.4	0.0	[17]

Supplementary Table 2. Functional classification and expression level of male and female *D. diadema* specimen, compared to Drukewitz et.al. 2018. Protein classes, which were not detected are marked with a X. Novel proteins that are unique for *D. diadema* are highlighted with the grey background colour.

Protein Family	TPM - *E.rufibarbis*	TPM - M.arthriticus	TPM - *D.diadema* female	TPM - *D.diadema* male
CAP	X	X	3909.3	4439.4
Chitinase	X	X	12205.1	13743.9
Hyaluronidase	X	X	X	575.3
Kunitz	X	X	558.3	X
MBF2-domain	X	31596.5	10816.2	7294.8
Metallopeptidase M13	663.2	X	520.1	420.9
Peptidase S1	X	56054.6	2588.1	28236.7
Phospholipase A2	X	2642.7	X	X
Venom acid phosphatase-like	X	3042.6	X	X
Asilidin$_1$	7930.8	12884.2	6052.2	7192.7
Asilidin$_2$	234658.8	288479.1	232454.1	197450.9
Asilidin$_3$	50168.4	167023.7	31727.7	48009.0
Asilidin$_4$	23893.2	23364.1	X	X
Asilidin$_5$	9686.6	X	4372.9	6874.7
Asilidin$_6$	X	3009.5	X	1488.9
Asilidin$_7$	X	484.4	X	X
Asilidin$_8$	1451.5	5900.1	X	X
Asilidin$_9$	1315.0	9420.5	X	1318.8
Asilidin$_{10}$	X	907.4	309.2	122.3

Novel	X	X	11632.29294	12958.1529
$Asilidin_{11}$	X	X	793.6	1141.7
$Asilidin_{12}$	X	X	720.8	X
$Asilidin_{13}$	X	X	X	817.4
$Asilidin_{14}$	X	X	546.0	X
$Asilidin_{15}$	X	X	1993.3	X
Similar expressed in BT	X	X	8904.5	9673.2

Supplementary Table 3. Accession to included genomes.

Species	Database
	NCBI
Proctacanthus coquiletti	https://www.ncbi.nlm.nih.gov/assembly/GCA_001932985.1/
	ENSEMBL
Aedes aegypti	ftp://ftp.ensemblgenomes.org/pub/metazoa/release-39/fasta/aedes_aegypti
Anopheles darlingi	ftp://ftp.ensemblgenomes.org/pub/metazoa/release-39/fasta/anopheles_darlingi
Anopheles gambiae	ftp://ftp.ensemblgenomes.org/pub/metazoa/release-39/fasta/anopheles_gambiae
Culex quinquefasciatus	ftp://ftp.ensemblgenomes.org/pub/metazoa/release-39/fasta/culex_quinquefasciatus
Mayetiola destructor	ftp://ftp.ensemblgenomes.org/pub/metazoa/release-39/fasta/mayetiola_destructor
Lucilia cuprina	ftp://ftp.ensemblgenomes.org/pub/metazoa/release-39/fasta/lucilia_cuprina
Teleopsis dalmanni	ftp://ftp.ensemblgenomes.org/pub/metazoa/release-39/fasta/teleopsis_dalmanni
Drosophila melanogaster	ftp://ftp.ensemblgenomes.org/pub/metazoa/release-39/fasta/drosophila_melanogaster
Drosophila simulans	ftp://ftp.ensemblgenomes.org/pub/metazoa/release-39/fasta/drosophila_simulans
Drosophila grimshawi	ftp://ftp.ensemblgenomes.org/pub/metazoa/release-39/fasta/drosophila_grimshawi
Bombyx mori	ftp://ftp.ensemblgenomes.org/pub/metazoa/release-39/fasta/bombyx_mori
Danaus plexippus	ftp://ftp.ensemblgenomes.org/pub/metazoa/release-39/fasta/danaus_plexippus

Supplementary Table 4: SRA-archive accession numbers of used RNA and DNA data.

	Species	Run - Accession	Tissue
RNA			
	Dasypogon diadema (female)	SRR7754485	venom glands
	Dasypogon diadema (male)	SRR7754486	venom glands
	Dasypogon diadema (female)	SRR7754487	proboscis tissue
	Dasypogon diadema (male)	SRR7754488	proboscis tissue
	Dasypogon diadema (female)	SRR5192547	thoracic musculature
	Dasypogon diadema (male)	SRR5192548	thoracic musculature
	Diogmites neoternatus	SRR4345333	thoracic musculature
	Tolmerus atricapillus	SRR4346294	thoracic musculature
	Laphystia limatula	SRR4346311	thoracic musculature
	Proctacanthus coquilletti	SRR4346725	thoracic musculature
	Scleropogon duncani	SRR4346727	thoracic musculature
	Philonicus albiceps	SRR4365562	thoracic musculature
	Eutolmus rufibarbis	SRR5185496	thoracic glands
	Eutolmus rufibarbis	SRR5185497	thoracic musculature
	Machimus arthriticus	SRR5185498	thoracic glands
	Machimus arthriticus	SRR5185499	thoracic musculature
DNA			
	Dasypogon diadema	SRR7878513.<br>SRR7878512.	thoracic musculature

Supplementary Table 5: Overview of RNA sequencing and processing of *D. diadema.*

		Raw – read pairs	Processed – read pairs
male	venom gland	26303932	18615442
	proboscis	23436521	16456042
	body tissue	25978091	18227143
female	venom gland	26316124	18636522
	proboscis	35727429	24581834
	body tissue	31018992	22370413

Supplementary Table 6: Overview of the predominant top 30 venom proteins and their associated orthogroups.

Protein Family	Orthogroup	Gene Annotation	Toxin name	number of genes in OG	node with LCA
	OG0001188	Dasypogon_round1.nuc|augustus_masked-jcf7180002951459-processed-gene-0.8-mRNA-1	U-Asilidin$_2$-Dd1a	multi Copy 3	node1
		Dasypogon_round1.nuc|augustus_masked-jcf7180002950151-processed-gene-0.20-mRNA-1	U-Asilidin$_2$-Dd7b	multi Copy 3	node5
	OG0009077	Dasypogon_round1.nuc|augustus_masked-jcf7180002950151-processed-gene-0.21-mRNA-1	U-Asilidin$_2$-Dd3a	multi Copy 3	node5
		Dasypogon_round1.nuc|augustus_masked-jcf7180002950151-processed-gene-0.18-mRNA-1	U-Asilidin$_2$-Dd8a	multi Copy 3	node5
Asilidin$_2$		Dasypogon_round1.nuc|augustus_masked-jcf7180002950151-processed-gene-0.19-mRNA-1	U-Asilidin$_2$-Dd7a	multi Copy 3	node5
		Dasypogon_round1.nuc|augustus_masked-jcf7180002947325-processed-gene-0.0-mRNA-1	U-Asilidin$_2$-Dd6a	multi Copy 3	node5
	OG0009368	Dasypogon_round1.nuc|maker-jcf7180002901206-snap-gene-0.1-mRNA-1	U-Asilidin$_2$-Dd2b	multi Copy 3	node5
		Dasypogon_round1.nuc|maker-jcf7180002952116-snap-gene-0.8-mRNA-1	U-Asilidin$_2$-Dd2a	multi Copy 3	node5
	OG0011154	Dasypogon_round1.nuc|augustus_masked-jcf7180002949739-processed-gene-0.9-mRNA-1	U-Asilidin$_2$-Dd4a	multi Copy 2	node5
		Dasypogon_round1.nuc|augustus_masked-jcf7180002943599-processed-gene-0.12-mRNA-1	U-Asilidin$_2$-Dd5a	multi Copy 2	node5
	OG0008094	Dasypogon_round1.nuc|augustus_masked-jcf7180002951216-processed-gene-0.0-mRNA-1		multi Copy 3	node4
Peptidase S1	OG0011159	Dasypogon_round1.nuc|augustus_masked-jcf7180002937304-processed-gene-0.0-mRNA-1		multi Copy 2	node5
	OG0009784	Dasypogon_round1.nuc|augustus_masked-jcf7180002935958-processed-gene-0.2-mRNA-1		multi Copy 3	node5
	OG0000005	Dasypogon_round1.nuc|maker-jcf7180002939427-snap-gene-0.8-mRNA-1		multi Copy 3	node1
	OG0000093	Dasypogon_round1.nuc|maker-jcf7180002952995-augustus-gene-0.16-mRNA-1		multi Copy 3	node1
Chitinase		Dasypogon_round1.nuc|maker-jcf7180002952995-augustus-gene-0.15-mRNA-1		multi Copy 3	node1
	OG0004761	Dasypogon_round1.nuc|maker-jcf7180002952995-augustus-gene-0.5-mRNA-1		single Copy	node2
MBF2_domain	OG0000122	Dasypogon_round1.nuc|maker-jcf7180002940395-snap-gene-0.10-mRNA-1		multi Copy 3	node1
		Dasypogon_round1.nuc|maker-jcf7180002940395-augustus-gene-0.18-mRNA-1		multi Copy 3	node1
CAP	OG0000016	Dasypogon_round1.nuc|maker-jcf7180002948866-snap-gene-0.2-mRNA-1		multi Copy 3	node1
Asilidin$_6$	OG0015182	Dasypogon_round1.nuc|maker-jcf7180002939636-augustus-gene-0.1-mRNA-1	U-Asilidin$_6$-Dd6a	single Copy	node5
Asilidin$_9$	OG0000267	Dasypogon_round1.nuc|maker-jcf7180002938705-augustus-gene-0.6-mRNA-1	U-Asilidin$_9$-Dd1a	multi Copy 3	node1
Asilidin$_{11}$	OG0001102	Dasypogon_round1.nuc|maker-jcf7180002952356-snap-gene-0.8-mRNA-1	U-Asilidin$_{11}$-Dd1a	multi Copy 3	node2
Asilidin$_{12}$	OG0001332	Dasypogon_round1.nuc|maker-jcf7180002949379-augustus-gene-0.4-mRNA-1	U-Asilidin$_{12}$-Dd1a	single Copy	node1
Asilidin$_{13}$	OG0003052	Dasypogon_round1.nuc|maker-jcf7180002950379-augustus-gene-0.7-mRNA-1	U-Asilidin$_{13}$-Dd1a	single Copy	node1
Asilidin$_{14}$	OG0007612	Dasypogon_round1.nuc|maker-jcf7180002948577-augustus-gene-0.2-mRNA-1	U-Asilidin$_{14}$-Dd1a	single Copy	node1
Asilidin$_{15}$	OG0000083	Dasypogon_round1.nuc|maker-jcf7180002932717-snap-gene-0.5-mRNA-1	U-Asilidin$_{15}$-Dd1a	multi Copy 3	node1
Asilidin$_3$		Dasypogon_round1.nuc|maker-jcf7180002939401-augustus-gene-0.1-mRNA-1	U-Asilidin$_3$-Dd1a	single Copy	no Orth
Asilidin$_3$	no Orthogroup	Dasypogon_round1.nuc|snap_masked-jcf7180002938241-processed-gene-0.1-mRNA-1	U-Asilidin$_3$-Dd1b	single Copy	no Orth
Asilidin$_1$		Dasypogon_round1.nuc|maker-jcf7180002939442-augustus-gene-0.0-mRNA-1	U-Asilidin$_1$-Dd1a	single Copy	no Orth

Supplementary Table 7: Overview of transposable elements in the predominant top 30 venom proteins.

Protein family	Ortho group	Gene Annotation	Scaffold	Start	End	Element	Family	Type	Strand
**Asilidin$_2$**	OG0001188	Dasypogon_round1.nuc|augustus_masked-jcf7180002951459-processed-gene-0.8-mRNA-1	jcf7180002951459	8109	8200	rnd-4_family-1524	DNA/TcMar-Tc1	Transposon	+
				8259	8407	rnd-5_family-578	DNA/TcMar-Tc1	Transposon	C
				11492	11639	rnd-1_family-178	LTR/Unknown	LTR	C
				11451	11532	rnd-1_family-231	LTR/Unknown	LTR	+
				7562	7802	rnd-4_family-509	LTR/Unknown	LTR	+
				4535	4649	rnd-5_family-538	LTR/Unknown	LTR	C
				5353	5692	rnd-5_family-538	LTR/Unknown	LTR	C
	OG0009077	Dasypogon_round1.nuc|augustus_masked-jcf7180002950151-processed-gene-0.20-mRNA-1	jcf7180002950151	-	-	-	-	no Transposon or LTR	
		Dasypogon_round1.nuc|augustus_masked-jcf7180002950151-processed-gene-0.21-mRNA-1		-	-	-	-	no Transposon or LTR	
		Dasypogon_round1.nuc|augustus_masked-jcf7180002950151-processed-gene-0.18-mRNA-1		-	-	-	-	no Transposon or LTR	
		Dasypogon_round1.nuc|augustus_masked-jcf7180002950151-processed-gene-0.19-mRNA-1		-	-	-	-	no Transposon or LTR	
	OG0009368	Dasypogon_round1.nuc|augustus_masked-jcf7180002947325-processed-gene-0.0-mRNA-1	jcf7180002947325	-	-	-	-	no Transposon or LTR	
		Dasypogon_round1.nuc|maker-jcf7180002901206-snap-gene-0.1-mRNA-1	jcf7180002901206	-	-	-	-	no Transposon or LTR	
		Dasypogon_round1.nuc|maker-jcf7180002952116-snap-gene-0.8-mRNA-1	jcf7180002952116	5035	5182	rnd-5_family-4538	LTR/Unknown	LTR	+
	OG0011154	Dasypogon_round1.nuc|augustus_masked-jcf7180002949739-processed-gene-0.9-mRNA-1	jcf7180002949739	-	-	-	-	no Transposon or LTR	
		Dasypogon_round1.nuc|augustus_masked-jcf7180002943599-processed-gene-0.12-mRNA-1	jcf7180002943599	-	-	-	-	no Transposon or LTR	
**Peptidase S1**	OG0008094	Dasypogon_round1.nuc|augustus_masked-jcf7180002951216-processed-gene-0.0-mRNA-1	jcf7180002951216	-	-	-	-	no Transposon or LTR	-
	OG0011159	Dasypogon_round1.nuc|augustus_masked-jcf7180002937304-processed-gene-0.0-mRNA-1	jcf7180002937304	-	-	-	-	no Transposon or LTR	-
	OG0009784	Dasypogon_round1.nuc|augustus_masked-jcf7180002935958-processed-gene-0.2-mRNA-1	jcf7180002935958	-	-	-	-	no Transposon or LTR	-
	OG0000005	Dasypogon_round1.nuc|maker-jcf7180002939427-snap-gene-0.8-mRNA-1	jcf7180002939427	6497	6605	rnd-1_family-756	DNA/TcMar-Mariner	Transposon	C
				3668	4045	rnd-1_family-25	LINE/CR1	Transposon	+
				656	739	rnd-1_family-152	LTR/Unknown	LTR	C

**Chitinase**	OG00 00093	Dasypogon_round1.nuc|maker-jcf7180002952995-augustus-gene-0.16-mRNA-1	jcf71800029529 95	-	-	-	-	no Transposon or LTR	-
		Dasypogon_round1.nuc|maker-jcf7180002952995-augustus-gene-0.15-mRNA-1		92418	92555	rnd-1_family-183	LTR/Unknown	LTR	C
	OG00 04761	Dasypogon_round1.nuc|maker-jcf7180002952995-augustus-gene-0.5-mRNA-1		-	-	-	-	no Transposon or LTR	-
**MBF2_do main**	OG00 00122	Dasypogon_round1.nuc|maker-jcf7180002940395-snap-gene-0.10-mRNA-1	jcf71800029403 95	33617	34192	rnd-1_family-151	LTR/Unknown	LTR	+
				30241	30331	rnd-1_family-161	LTR/Unknown	LTR	+
				29807	29970	rnd-1_family-178	LTR/Unknown	LTR	C
				30003	30162	rnd-1_family-178	LTR/Unknown	LTR	+
				29750	29845	rnd-1_family-231	LTR/Unknown	LTR	+
				30123	30219	rnd-1_family-231	LTR/Unknown	LTR	C
				33798	33916	rnd-1_family-39	LTR/Unknown	LTR	C
				31135	31245	rnd-3_family-14	LTR/Unknown	LTR	+
		Dasypogon_round1.nuc|maker-jcf7180002940395-augustus-gene-0.18-mRNA-1	jcf71800029403 95	-	-	-	-	no Transposon or LTR	-
**CAP**	OG00 00016	Dasypogon_round1.nuc|maker-jcf7180002948866-snap-gene-0.2-mRNA-1	jcf71800029488 66	-	-	-	-	no Transposon or LTR	-
**Asilidin$_6$**	OG00 15182	Dasypogon_round1.nuc|maker-jcf7180002939636-augustus-gene-0.1-mRNA-1	jcf71800029396 36	9688	9839	rnd-1_family-0	LTR/Unknown	LTR	C
			jcf71800029396 36	9838	10082	rnd-1_family-1	LTR/Unknown	LTR	C
**Asilidin$_9$**	OG00 00267	Dasypogon_round1.nuc|maker-jcf7180002938705-augustus-gene-0.6-mRNA-1	jcf71800029387 05	14659	14781	rnd-1_family-323	LINE/LOA	Transposon	+
				14588	14674	rnd-1_family-783	LINE/LOA	Transposon	+
				7721	7958	rnd-5_family-699	LINE/LOA	Transposon	+
				11981	12108	rnd-1_family-184	LTR/Unknown	LTR	+
**Asilidin$_{11}$**	OG00 01102	Dasypogon_round1.nuc|maker-jcf7180002952356-snap-gene-0.8-mRNA-1	jcf71800029523 56	10252	10478	rnd-5_family-863	LINE/L1	Transposon	C
				9647	9759	rnd-5_family-9393	LINE/L1-Tx1	Transposon	C
				8850	8963	rnd-5_family-7535	LTR/Unknown	LTR	+
**Asilidin$_{12}$**	OG00 01332	Dasypogon_round1.nuc|maker-jcf7180002949379-augustus-gene-0.4-mRNA-1	jcf71800029493 79	16052	16603	rnd-1_family-280	DNA/TcMar-Mariner	Transposon	C
				15138	15331	rnd-1_family-443	DNA/TcMar-Mariner	Transposon	C
				20314	20425	rnd-5_family-5882	DNA/TcMar-Mariner	Transposon	C
				9135	9440	rnd-1_family-82	LINE/L2	Transposon	+

				6607	6774	rnd-5_family-934	LINE/L2	Transposon	C
				6851	6985	rnd-6_family-6957	LINE/L2	Transposon	C
				17241	17326	rnd-5_family-2497	LINE/Penelope	Transposon	+
				19873	19993	rnd-1_family-842	LTR/Unknown	LTR	+
				13676	13776	rnd-4_family-1505	LTR/Unknown	LTR	C
				19737	19816	rnd-6_family-8251	LTR/Unknown	LTR	C
**Asilidin$_{13}$**	OG0003052	Dasypogon_round1.nuc|maker-jcf7180002950379-augustus-gene-0.7-mRNA-1	jcf7180002950379	13297	13404	rnd-6_family-9337	DNA/TcMar-Tc1	Transposon	+
				14074	15386	rnd-3_family-444	LINE/CR1	Transposon	C
				15678	16606	rnd-3_family-444	LINE/CR1	Transposon	C
				12090	12397	rnd-1_family-0	LTR/Unknown	LTR	C
				12956	13279	rnd-1_family-0	LTR/Unknown	LTR	C
				17140	17286	rnd-1_family-432	LTR/Unknown	LTR	C
				16936	17175	rnd-1_family-885	LTR/Unknown	LTR	+
				15432	15656	rnd-5_family-142	LTR/Unknown	LTR	C
				16652	16931	rnd-5_family-142	LTR/Unknown	LTR	C
				11859	12096	rnd-5_family-2714	LTR/Unknown	LTR	C
				12702	12962	rnd-5_family-2714	LTR/Unknown	LTR	C
**Asilidin$_{14}$**	OG0007612	Dasypogon_round1.nuc|maker-jcf7180002948577-augustus-gene-0.2-mRNA-1	jcf7180002948577	-	-	-	-	no Transposon or LTR	-
**Asilidin$_{15}$**	OG0000083	Dasypogon_round1.nuc|maker-jcf7180002932717-snap-gene-0.5-mRNA-1	jcf7180002932717	5495	5618	rnd-1_family-77	LTR/Unknown	LTR	+
				5752	5877	rnd-1_family-77	LTR/Unknown	LTR	C
**Asilidin$_{3}$**	no Orthogroup	Dasypogon_round1.nuc|maker-jcf7180002939401-augustus-gene-0.1-mRNA-1	jcf7180002939401	-	-	-	-	no Transposon or LTR	-
		Dasypogon_round1.nuc|snap_masked-jcf7180002938241-processed-gene-0.1-mRNA-1	jcf7180002938241	-	-	-	-	no Transposon or LTR	-
**Asilidin$_{1}$**		Dasypogon_round1.nuc|maker-jcf7180002939442-augustus-gene-0.0-mRNA-1	jcf7180002939442	-	-	-	-	no Transposon or LTR	-

Supplementary Table 8: Overview of contigs for the top 30 toxin candidates that were identified in the RNASpades assembly. All relevant comparative alignments are given in the GigaScience data cloud.

gene	protein	tox-name	male-trans	male-id	female-trans	female-id
Dasypogon_round1.pep_augustus_masked-jcf7180002935958-processed-gene-0.2-mRNA-1	Peptidase S1	X	Male_VG_TRINITY_DN13880_c1_g1_i12|m.14446 vs NODE_5441_length_1098_cov_7568.324118_g4747_i0.p1	0.888	X	X
Dasypogon_round1.pep_augustus_masked-jcf7180002937304-processed-gene-0.0-mRNA-1	Peptidase S1	X	Male_VG_TRINITY_DN13419_c0_g2_i2|m.24524 vs NODE_4542_length_1283_cov_2910.491896_g2880_i2.p1	0.996	Female_VG_TRINITY_DN12070_c0_g1_i1|m.15154 vs NODE_4576_length_1314_cov_2630.113834_g3996_i0.p1	1
Dasypogon_round1.pep_augustus_masked-jcf7180002943599-processed-gene-0.12-mRNA-1	Asilidin2	U-Asilidin2-Dd5a	Male_VG_TRINITY_DN13400_c0_g1_i2|m.25371 vs NODE_5000_length_1182_cov_6873.450132_g4349_i0.p1	0.982	Female_VG_TRINITY_DN12595_c1_g6_i2|m.72996 vs NODE_1557_length_2380_cov_19719.782068_g1353_i0.p1	0.996
Dasypogon_round1.pep_augustus_masked-jcf7180002947325-processed-gene-0.0-mRNA-1	Asilidin2	U-Asilidin2-Dd6a	Male_VG_TRINITY_DN13853_c1_g5_i5|m.9684 vs NODE_6147_length_966_cov_39395.704471_g2057_i4.p1	0.586	Female_VG_TRINITY_DN12824_c1_g2_i1|m.97629 vs NODE_5737_length_1080_cov_51394.530553_g5036_i0.p1	0.588
Dasypogon_round1.pep_augustus_masked-jcf7180002949739-processed-gene-0.9-mRNA-1	Asilidin2	U-Asilidin2-Dd4a	Male_VG_TRINITY_DN13746_c1_g1_i7|m.55977 vs NODE_1678_length_2269_cov_21580.102252_g1031_i1.p1	1	Female_VG_TRINITY_DN12595_c1_g6_i2|m.72996 vs NODE_1557_length_2380_cov_19719.782068_g1353_i0.p1	0.996
Dasypogon_round1.pep_augustus_masked-jcf7180002950151-processed-gene-0.18-mRNA-1	Asilidin2	U-Asilidin2-Dd8a	Male_VG_TRINITY_DN13737_c1_g1_i5|m.59855 vs NODE_1782_length_2218_cov_25758.051637_g1544_i0.p1	0.964	Female_VG_TRINITY_DN12734_c1_g2_i5|m.28769 vs NODE_1690_length_2309_cov_26804.106637_g1468_i0.p1	1
Dasypogon_round1.pep_augustus_masked-jcf7180002950151-processed-gene-0.19-mRNA-1	Asilidin2	U-Asilidin2-Dd7a	Male_VG_TRINITY_DN13180_c0_g4_i1|m.46072 vs NODE_3891_length_1445_cov_9511.679799_g3369_i0.p1	0.972	Female_VG_TRINITY_DN11814_c0_g1_i1|m.32439 vs NODE_3397_length_1621_cov_9254.910305_g2954_i0.p1	1
Dasypogon_round1.pep_augustus_masked-jcf7180002950151-processed-gene-0.20-mRNA-1	Asilidin2	U-Asilidin2-Dd7b	Male_VG_TRINITY_DN13360_c0_g3_i1|m.70997 vs NODE_3200_length_1644_cov_3730.558621_g2777_i0.p1	0.689	Female_VG_TRINITY_DN12240_c1_g1_i4|m.45172 vs NODE_3229_length_1674_cov_4962.355077_g2455_i3.p1	0.87
Dasypogon_round1.pep_augustus_masked-jcf7180002950151-processed-gene-0.21-mRNA-1	Asilidin2	U-Asilidin2-Dd3a	Male_VG_TRINITY_DN13507_c0_g1_i2|m.27600 vs NODE_5142_length_1160_cov_16221.472547_g4482_i0.p1	0.855	Female_VG_TRINITY_DN11685_c0_g1_i1|m.5314 vs NODE_2186_length_2078_cov_10043.935929_g1046_i2.p1	0.972
Dasypogon_round1.pep_augustus_masked-jcf7180002951216-processed-gene-0.0-mRNA-1	Peptidase S1	X	Male_VG_TRINITY_DN13447_c0_g2_i1|m.26636 vs NODE_4737_length_1235_cov_2596.062395_g4059_i1.p1	0.997	X	X
Dasypogon_round1.pep_augustus_masked-jcf7180002951459-processed-gene-0.8-mRNA-1	Asilidin2	U-Asilidin2-Dd1a	Male_VG_TRINITY_DN13732_c1_g2_i4|m.57621 vs NODE_2309_length_1971_cov_86155.223725_g1993_i0.p1	1	Female_VG_TRINITY_DN12750_c0_g9_i2|m.29883 vs NODE_2439_length_1958_cov_78239.987952_g835_i6.p1	1
Dasypogon_round1.pep_maker-jcf7180002901206-snap-gene-0.1-mRNA-1	Asilidin2	U-Asilidin2-Dd2b	X	X	Female_VG_TRINITY_DN12824_c1_g2_i1|m.97629 vs NODE_5737_length_1080_cov_51394.530553_g5036_i0.p1	0.588
Dasypogon_round1.pep_maker-jcf7180002932717-snap-gene-0.5-mRNA-1	Asilidin15	U-Asilidin15-Dd1a	Male_VG_TRINITY_DN11032_c0_g1_i1|m.65113 vs NODE_7946_length_717_cov_3337.660180_g7059_i0.p1	0.952	X	X
Dasypogon_round1.pep_maker-jcf7180002938705-augustus-gene-0.6-mRNA-1	Asilidin9	U-Asilidin9-Dd1a	Male_VG_TRINITY_DN13550_c0_g1_i11|m.28118 vs NODE_9049_length_603_cov_4068.007220_g7492_i1.p1	0.746	X	X
Dasypogon_round1.pep_maker-jcf7180002939401-augustus-gene-0.1-mRNA-1	Asilidin3	U-Asilidin3-Dd1a	Male_VG_TRINITY_DN13614_c0_g1_i4|m.91232 vs NODE_2316_length_1969_cov_9881.662500_g844_i2.p1	0.717	X	X
Dasypogon_round1.pep_maker-jcf7180002939427-snap-gene-0.8-mRNA-1	Peptidase S1	X	Male_VG_TRINITY_DN13700_c0_g3_i2|m.58025 vs NODE_4553_length_1280_cov_3386.460601_g3746_i1.p1	1	Female_VG_TRINITY_DN11949_c0_g1_i1|m.35950 vs NODE_5265_length_1166_cov_4405.076992_g4357_i1.p1	0.982

Dasypogon_round1.pep_maker-jcf7180002939442-augustus-gene-0.0-mRNA-1	Asilidin1	U-Asilidin1-Dd1a	Male_VG_TRINITY_DN13874_c0_g1_i10|m.11872 vs NODE_15823_length_301_cov_13608.650794_g14856_i0.p1	0.965	Female_VG_TRINITY_DN11100_c0_g1_i7|m.62052 vs NODE_4920_length_1240_cov_2771.075567_g4294_i0.p2	0.988
Dasypogon_round1.pep_maker-jcf7180002939636-augustus-gene-0.1-mRNA-1	Asilidin6	U-Asilidin6-Dd6a	Male_VG_TRINITY_DN13654_c1_g2_i5|m.90975 vs NODE_8997_length_607_cov_9977.777778_g549_i2.p1	1	X	X
Dasypogon_round1.pep_maker-jcf7180002940395-augustus-gene-0.18-mRNA-1	MBF2 domain	X	Male_VG_TRINITY_DN11280_c0_g1_i4|m.18861 vs NODE_9009_length_606_cov_8383.224417_g7609_i1.p1	0.958	Female_VG_TRINITY_DN12750_c0_g4_i2|m.29860 vs NODE_9538_length_584_cov_15679.360748_g8544_i0.p1	0.983
Dasypogon_round1.pep_maker-jcf7180002940395-snap-gene-0.10-mRNA-1	MBF2 domain	X	Male_VG_TRINITY_DN13740_c0_g3_i11|m.58949 vs NODE_5329_length_1120_cov_569.453782_g906_i2.p1	0.992	X	X
Dasypogon_round1.pep_maker-jcf7180002948577-augustus-gene-0.2-mRNA-1	Asilidin14	U-Asilidin14-Dd1a	Male_VG_TRINITY_DN11302_c0_g2_i1|m.84646 vs NODE_4456_length_1302_cov_950.958500_g3639_i1.p1	1	X	X
Dasypogon_round1.pep_maker-jcf7180002948866-snap-gene-0.2-mRNA-1	CAP	X	Male_VG_TRINITY_DN13232_c1_g2_i1|m.101484 vs NODE_2577_length_1864_cov_9045.001102_g1764_i1.p1	1	Female_VG_TRINITY_DN12505_c0_g1_i1|m.73039 vs NODE_7202_length_845_cov_18.527638_g6356_i0.p1	0.542
Dasypogon_round1.pep_maker-jcf7180002949379-augustus-gene-0.4-mRNA-1	Asilidin12	U-Asilidin12-Dd1a	Male_VG_TRINITY_DN13836_c0_g2_i4|m.11439 vs NODE_198_length_4257_cov_1083.985266_g43_i1.p1	0.976	X	X
Dasypogon_round1.pep_maker-jcf7180002950379-augustus-gene-0.7-mRNA-1	Asilidin13	U-Asilidin13-Dd1a	Male_VG_TRINITY_DN12035_c0_g1_i2|m.18146 vs NODE_7070_length_827_cov_1817.066838_g6245_i0.p1	1	Female_VG_TRINITY_DN11289_c0_g1_i4|m.16850 vs NODE_6742_length_910_cov_1569.407666_g5580_i1.p1	1
Dasypogon_round1.pep_maker-jcf7180002952116-snap-gene-0.8-mRNA-1	Asilidin2	U-Asilidin2-Dd2a	X	X	Female_VG_TRINITY_DN12795_c1_g1_i8|m.30678 vs NODE_5061_length_1208_cov_49555.140638_g3774_i1.p1	0.578
Dasypogon_round1.pep_maker-jcf7180002952356-snap-gene-0.8-mRNA-1	Asilidin11	U-Asilidin11-Dd1a	Male_VG_TRINITY_DN13864_c0_g2_i2|m.12461 vs NODE_3401_length_1583_cov_1571.978488_g2947_i0.p1	1	Female_VG_TRINITY_DN12851_c2_g1_i2|m.95927 vs NODE_4888_length_1247_cov_2923.207846_g1486_i2.p1	0.997
Dasypogon_round1.pep_maker-jcf7180002952995-augustus-gene-0.15-mRNA-1	Chitinase	X	Male_VG_TRINITY_DN12940_c0_g1_i1|m.62397 vs NODE_5809_length_1028_cov_4204.799796_g4792_i1.p1	1	Female_VG_TRINITY_DN11120_c0_g2_i4|m.63927 vs NODE_6801_length_900_cov_8896.306698_g5411_i2.p2	1
Dasypogon_round1.pep_maker-jcf7180002952995-augustus-gene-0.16-mRNA-1	Chitinase	X	Male_VG_TRINITY_DN13215_c0_g1_i2|m.101856 vs NODE_4027_length_1414_cov_4604.705495_g3271_i1.p1	1	Female_VG_TRINITY_DN10900_c0_g1_i3|m.7664 vs NODE_6508_length_946_cov_6895.909699_g5402_i1.p1	1
Dasypogon_round1.pep_maker-jcf7180002952995-augustus-gene-0.5-mRNA-1	Chitinase	X	Male_VG_TRINITY_DN10264_c0_g1_i1|m.66826 vs NODE_15120_length_316_cov_15856.764045_g14153_i0.p1	0.988	Female_VG_TRINITY_DN8860_c0_g1_i1|m.77404 vs NODE_12543_length_407_cov_9040.955307_g10133_i4.p1	1
Dasypogon_round1.pep_snap_masked-jcf7180002938241-processed-gene-0.1-mRNA-1	Asilidin3	U-Asilidin3-Dd1b	Male_VG_TRINITY_DN13600_c0_g1_i3|m.91739 vs NODE_605_length_3269_cov_4187.995963_g522_i0.p1	1	Female_VG_TRINITY_DN12746_c0_g1_i3|m.31318 vs NODE_1614_length_2351_cov_7541.256299_g1386_i1.p1	0.98

Supplementary Table 9: Overview of the contigs for the top 30 toxin candidates that were identified in the TransAbyss assembly. All relevant comparative alignments are given in the GigaScience data cloud.

gene	protein	tox-name	male-trans	male-id	female-trans	female-id
Dasypogon_round1.pep_augustus_masked-jcf7180002935958-processed-gene-0.2-mRNA-1	Peptidase S1	X	Male_VG_TRINITY_DN13880_c1_g1_i12|m.14446 vs R451279.p1	0.996	X	X
Dasypogon_round1.pep_augustus_masked-jcf7180002937304-processed-gene-0.0-mRNA-1	Peptidase S1	X	Male_VG_TRINITY_DN13419_c0_g2_i2|m.24524 vs R451558.p1	0.996	Female_VG_TRINITY_DN12070_c0_g1_i1|m.15154 vs R472831.p1	1
Dasypogon_round1.pep_augustus_masked-jcf7180002943599-processed-gene-0.12-mRNA-1	Asilidin2	U-Asilidin2-Dd5a	Male_VG_TRINITY_DN13400_c0_g1_i2|m.25371 vs R451412.p1	0.982	Female_VG_TRINITY_DN12595_c1_g6_i2|m.72996 vs R472523.p1	1
Dasypogon_round1.pep_augustus_masked-jcf7180002947325-processed-gene-0.0-mRNA-1	Asilidin2	U-Asilidin2-Dd6a	Male_VG_TRINITY_DN13853_c1_g5_i5|m.9684 vs R451273.p1	0.98	Female_VG_TRINITY_DN12824_c1_g2_i1|m.97629 vs R472519.p1	0.969
Dasypogon_round1.pep_augustus_masked-jcf7180002949739-processed-gene-0.9-mRNA-1	Asilidin2	U-Asilidin2-Dd4a	Male_VG_TRINITY_DN13746_c1_g1_i7|m.55977 vs R451277.p1	1	Female_VG_TRINITY_DN12595_c1_g6_i2|m.72996 vs R472523.p1	1
Dasypogon_round1.pep_augustus_masked-jcf7180002950151-processed-gene-0.18-mRNA-1	Asilidin2	U-Asilidin2-Dd8a	Male_VG_TRINITY_DN13737_c1_g1_i5|m.59855 vs R451272.p1	1	Female_VG_TRINITY_DN12734_c1_g2_i5|m.28769 vs R472518.p1	0.964
Dasypogon_round1.pep_augustus_masked-jcf7180002950151-processed-gene-0.19-mRNA-1	Asilidin2	U-Asilidin2-Dd7a	Male_VG_TRINITY_DN13180_c0_g4_i1|m.46072 vs R451375.p1	0.972	Female_VG_TRINITY_DN11814_c0_g1_i1|m.32439 vs R472614.p1	0.997
Dasypogon_round1.pep_augustus_masked-jcf7180002950151-processed-gene-0.20-mRNA-1	Asilidin2	U-Asilidin2-Dd7b	Male_VG_TRINITY_DN13360_c0_g3_i1|m.70997 vs R451490.p1	0.825	Female_VG_TRINITY_DN12240_c1_g1_i4|m.45172 vs R472623.p1	0.896
Dasypogon_round1.pep_augustus_masked-jcf7180002950151-processed-gene-0.21-mRNA-1	Asilidin2	U-Asilidin2-Dd3a	Male_VG_TRINITY_DN13507_c0_g1_i2|m.27600 vs R451368.p1	0.512	Female_VG_TRINITY_DN11685_c0_g1_i1|m.5314 vs R472584.p1	0.863
Dasypogon_round1.pep_augustus_masked-jcf7180002951216-processed-gene-0.0-mRNA-1	Peptidase S1	X	Male_VG_TRINITY_DN13447_c0_g2_i1|m.26636 vs R451617.p1	0.994	X	X
Dasypogon_round1.pep_augustus_masked-jcf7180002951459-processed-gene-0.8-mRNA-1	Asilidin2	U-Asilidin2-Dd1a	Male_VG_TRINITY_DN13732_c1_g2_i4|m.57621 vs R451270.p1	1	Female_VG_TRINITY_DN12750_c0_g9_i2|m.29883 vs R472529.p1	0.542
Dasypogon_round1.pep_maker-jcf7180002901206-snap-gene-0.1-mRNA-1	Asilidin2	U-Asilidin2-Dd2b	X	X	Female_VG_TRINITY_DN12824_c1_g2_i1|m.97629 vs Female_VG_TRINITY_DN12824_c1_g2_i1|m.97629	1
Dasypogon_round1.pep_maker-jcf7180002932717-snap-gene-0.5-mRNA-1	Asilidin15	U-Asilidin15-Dd1a	Male_VG_TRINITY_DN11032_c0_g1_i1|m.65113 vs R451540.p1	0.986	X	X
Dasypogon_round1.pep_maker-jcf7180002938705-augustus-gene-0.6-mRNA-1	Asilidin9	U-Asilidin9-Dd1a	Male_VG_TRINITY_DN13550_c0_g1_i11|m.28118 vs R451430.p1	1	X	X
Dasypogon_round1.pep_maker-jcf7180002939401-augustus-gene-0.1-mRNA-1	Asilidin3	U-Asilidin3-Dd1a	Male_VG_TRINITY_DN13614_c0_g1_i4|m.91232 vs R451276.p1	0.86	X	X
Dasypogon_round1.pep_maker-jcf7180002939427-snap-gene-0.8-mRNA-1	Peptidase S1	X	Male_VG_TRINITY_DN13700_c0_g3_i2|m.58025 vs R451492.p1	1	Female_VG_TRINITY_DN11949_c0_g1_i1|m.35950 vs R472735.p1	0.996
Dasypogon_round1.pep_maker-jcf7180002939442-augustus-gene-0.0-mRNA-1	Asilidin1	U-Asilidin1-Dd1a	Male_VG_TRINITY_DN13874_c0_g1_i10|m.11872 vs R451287.p2	1	Female_VG_TRINITY_DN11100_c0_g1_i7|m.62052 vs R472596.p1	0.988
Dasypogon_round1.pep_maker-jcf7180002939636-augustus-gene-0.1-mRNA-1	Asilidin6	U-Asilidin6-Dd6a	Male_VG_TRINITY_DN13654_c1_g2_i5|m.90975 vs R451406.p1	0.948	X	X
Dasypogon_round1.pep_maker-jcf7180002940395-augustus-gene-0.18-mRNA-1	MBF2 domain	X	Male_VG_TRINITY_DN11280_c0_g1_i4|m.18861 vs R451394.p1	0.975	Female_VG_TRINITY_DN12750_c0_g4_i2|m.29860 vs R472582.p1	0.992
Dasypogon_round1.pep_maker-jcf7180002940395-snap-gene-0.10-mRNA-1	MBF2 domain	X	Male_VG_TRINITY_DN13740_c0_g3_i11|m.58949 vs R451783.p1	0.992	X	X
Dasypogon_round1.pep_maker-jcf7180002948577-augustus-gene-0.2-mRNA-1	Asilidin14	U-Asilidin14-Dd1a	Male_VG_TRINITY_DN11302_c0_g2_i1|m.84646 vs R451890.p1	1	X	X

Dasypogon_round1.pep_maker-jcf7180002948866-snap-gene-0.2-mRNA-1	CAP	X	Male_VG_TRINITY_DN13232_c1_g2_i1|m.101484 vs R451334.p1	1	Female_VG_TRINITY_DN12505_c0_g1_i1|m.73039 vs R472600.p1	0.802
Dasypogon_round1.pep_maker-jcf7180002949379-augustus-gene-0.4-mRNA-1	Asilidin12	U-Asilidin12-Dd1a	Male_VG_TRINITY_DN13836_c0_g2_i4|m.11439 vs R451608.p1	0.88	X	X
Dasypogon_round1.pep_maker-jcf7180002950379-augustus-gene-0.7-mRNA-1	Asilidin13	U-Asilidin13-Dd1a	Male_VG_TRINITY_DN12035_c0_g1_i2|m.18146 vs R451729.p1	1	Female_VG_TRINITY_DN11289_c0_g1_i4|m.16850 vs R473014.p1	1
Dasypogon_round1.pep_maker-jcf7180002952116-snap-gene-0.8-mRNA-1	Asilidin2	U-Asilidin2-Dd2a		X	Female_VG_TRINITY_DN12795_c1_g1_i8|m.30678 vs R472520.p1	0.569
Dasypogon_round1.pep_maker-jcf7180002952356-snap-gene-0.8-mRNA-1	Asilidin11	U-Asilidin11-Dd1a	Male_VG_TRINITY_DN13864_c0_g2_i2|m.12461 vs R451736.p1	1	Female_VG_TRINITY_DN12851_c2_g1_i2|m.95927 vs R472915.p1	0.997
Dasypogon_round1.pep_maker-jcf7180002952995-augustus-gene-0.15-mRNA-1	Chitinase	X	Male_VG_TRINITY_DN12940_c0_g1_i1|m.62397 vs R451396.p1	1	Female_VG_TRINITY_DN11120_c0_g2_i4|m.63927 vs R472579.p2	1
Dasypogon_round1.pep_maker-jcf7180002952995-augustus-gene-0.16-mRNA-1	Chitinase	X	Male_VG_TRINITY_DN13215_c0_g1_i2|m.101856 vs R451388.p1	1	Female_VG_TRINITY_DN10900_c0_g1_i3|m.7664 vs R472651.p1	1
Dasypogon_round1.pep_maker-jcf7180002952995-augustus-gene-0.5-mRNA-1	Chitinase	X	Male_VG_TRINITY_DN10264_c0_g1_i1|m.66826 vs R451340.p1	1	Female_VG_TRINITY_DN8860_c0_g1_i1|m.77404 vs R472649.p1	1
Dasypogon_round1.pep_snap_masked-jcf7180002938241-processed-gene-0.1-mRNA-1	Asilidin3	U-Asilidin3-Dd1b	Male_VG_TRINITY_DN13600_c0_g1_i3|m.91739 vs R451280.p1	1	Female_VG_TRINITY_DN12746_c0_g1_i3|m.31318 vs R472526.p1	1

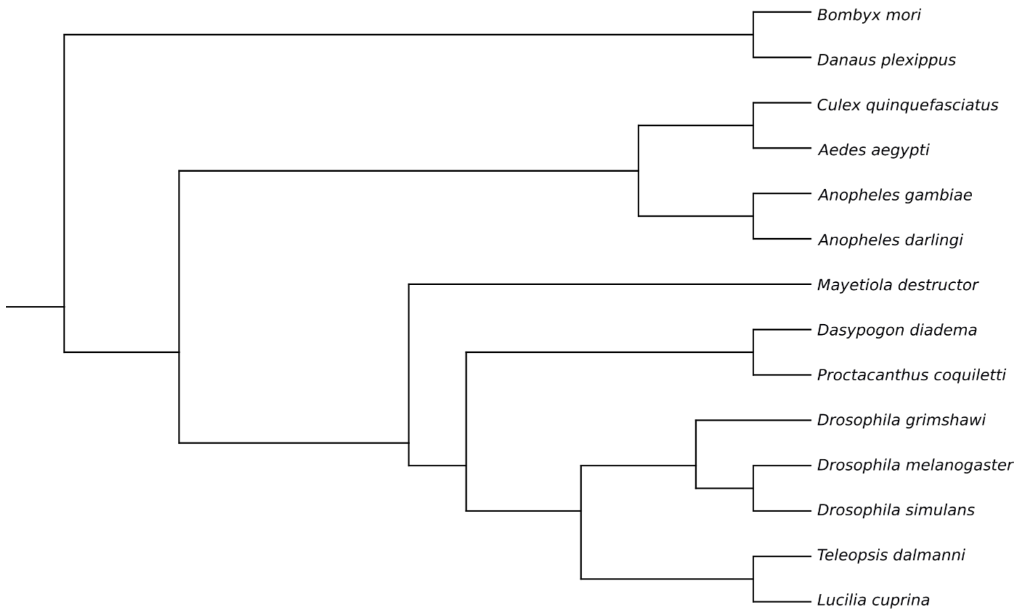

Supplementary Figure 1: Phylogenetic relationship of the analysed species.

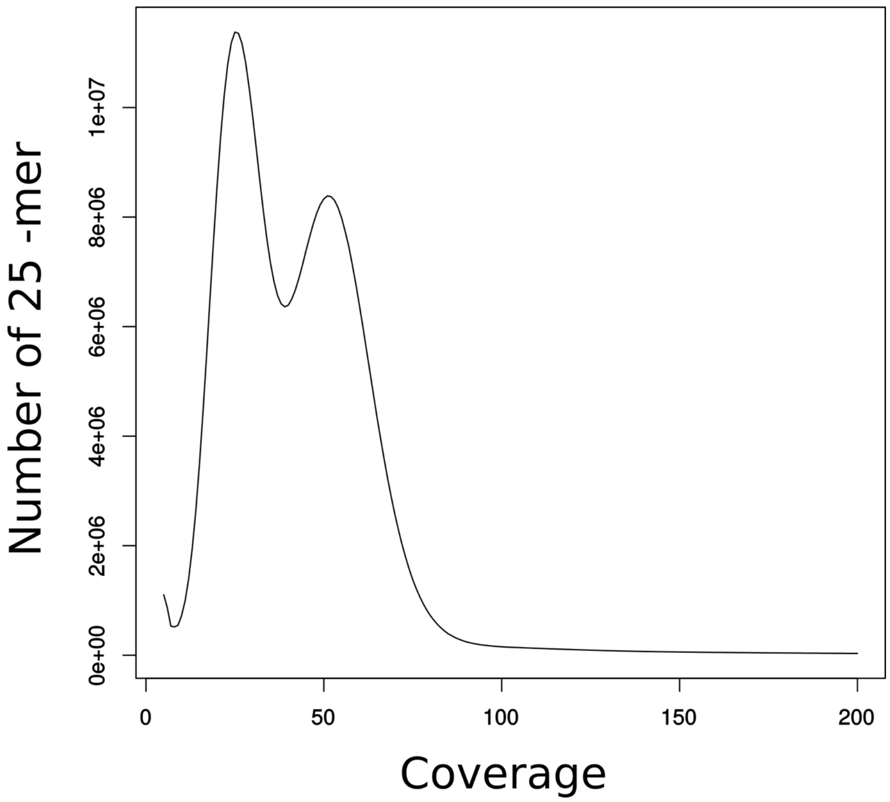

Supplementary Figure 2: Histogram of k-mer distribution using 25-mer in Jellyfish on the trimmed DNA-Seq data used for the assembly of the *D. diadema* genome.

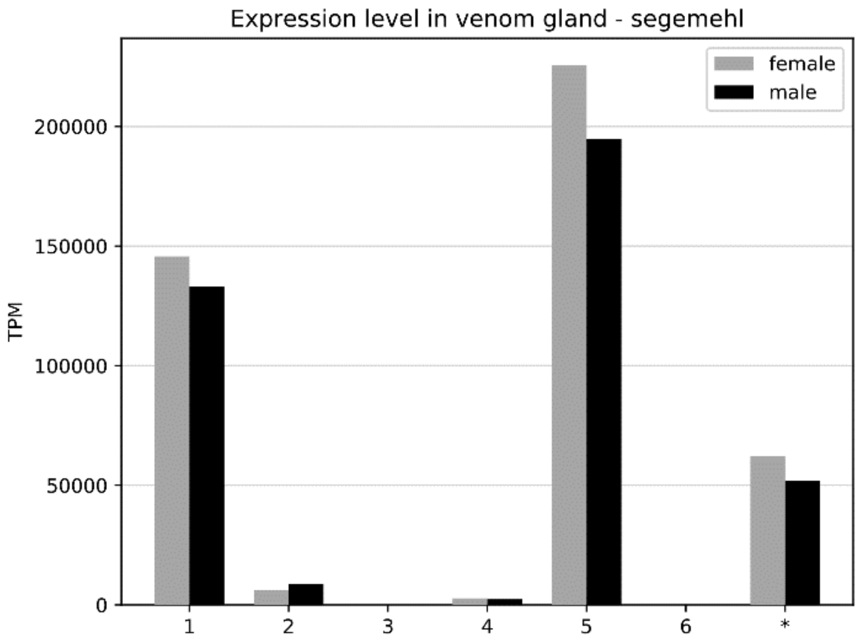

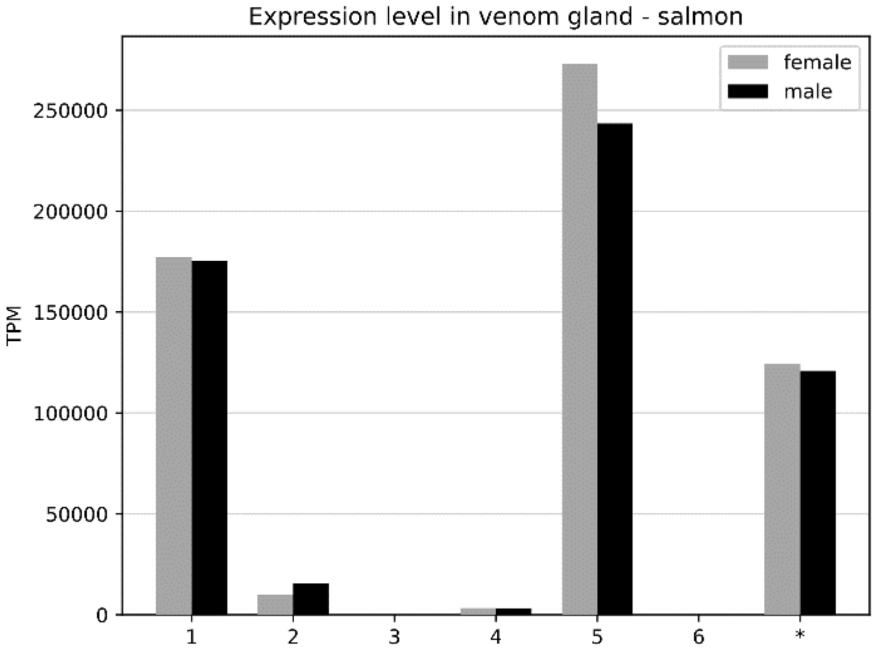

Supplementary Figure 3: Summarized expression level of putative toxins sorted to the associated phylogenetic split. RNA-Seq quantification was conducted with Segemehl (upper) and Salmon (lower)

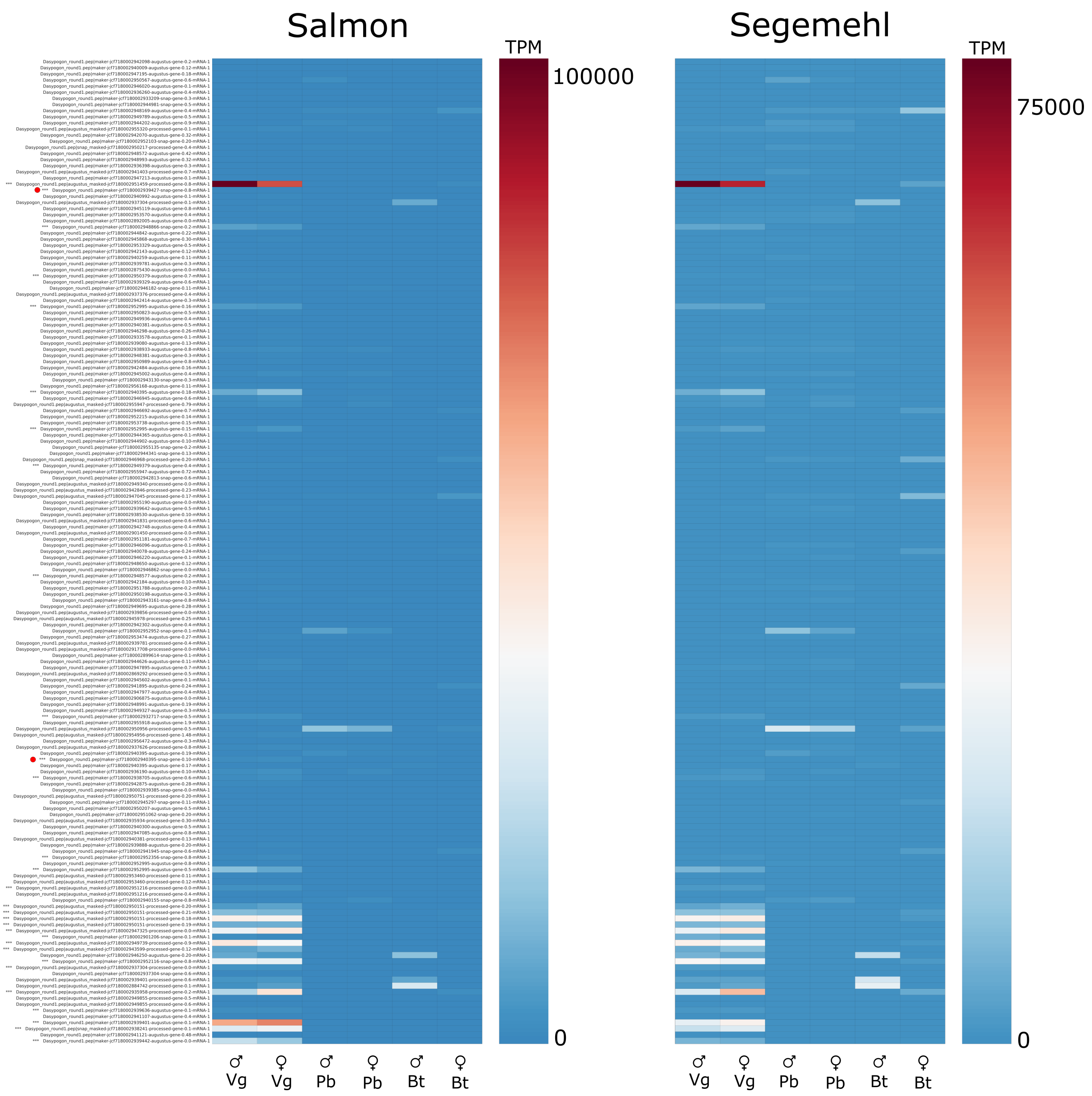

Supplementary Figure 4*:* Comparison of heatmaps based on the RNA-Seq quantification using Salmon (left) and Segemehl (right). Protein-coding genes that match the threshold in Salmon are marked with ***; the same genes also fulfil the threshold for Segemehl, except the one marked with the red dot. *(Vg-♂: venom gland male; Vg-♀: venom gland female; Pb-♂: proboscis male; Pb-♀: proboscis female; Bt-♂: body tissue male; Bt-♀: body tissue female)*

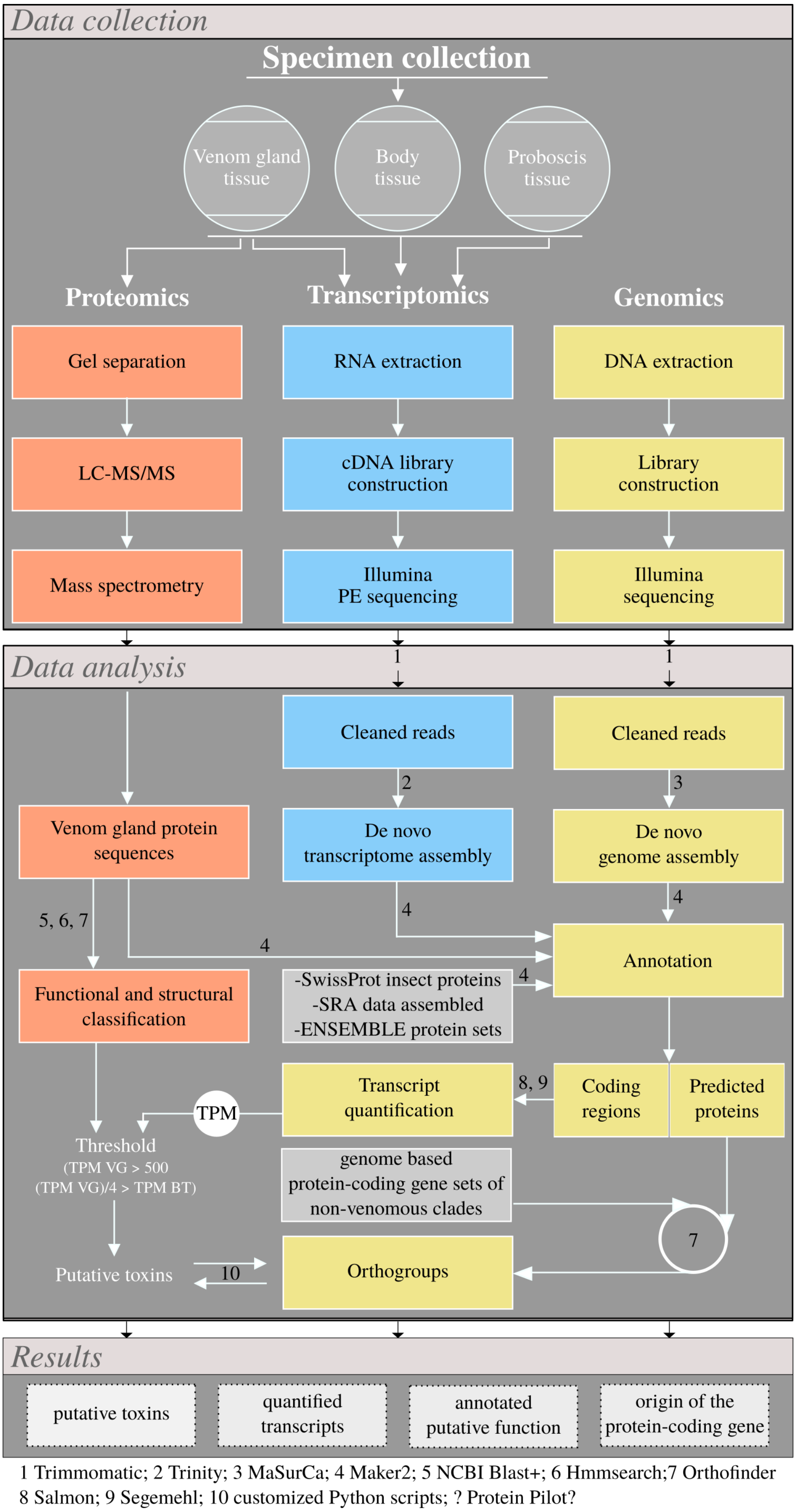

Supplementary Figure 5: Overview of the analysis workflow. 1-Trimmomatic; 2-Trinity; 3-MaSurCa; 4-Maker2; 5-NCBI Blast+; 6-Hmmsearch; 7-Orthofinder; 8-Salmon; 9; Segemehl; 10-Customized Python Scripts;

References

1. Martinson EO. Mrinalini. Kelkar YD. Chang CH. Werren JH. The evolution of venom by co-option of single-copy genes. Curr. Biol. 2017;27:2007–2013.e8.

2. Hoffman DR. Allergens in bee venom. III. Identification of allergen B of bee venom as an acid phosphatase. J. Allergy Clin. Immunol. 1977;59:364–6.

3. Barboni E. Kemeny DM. Campos S. Vernon CA. The purification of acid phosphatase from honey bee venom (*Apis mellifica*). Toxicon. 1987;25:1097–103.

4. GAULDIE J. HANSON JM. RUMJANEK FD. SHIPOLINI RA. VERNON CA. The Peptide Components of Bee Venom. Eur. J. Biochem. 1976;61:369–76.

5. Baer H. Liu TY. Anderson MC. Blum M. Schmid WH. James FJ. Protein components of fire ant venom (*Solenopsis invicta*). Toxicon. 1979;17:397–405.

6. Hoffman DR. Ant venoms. Curr. Opin. Allergy Clin. Immunol. 2010. p. 342–6.

7. Sanggaard KW. Bechsgaard JS. Fang X. Duan J. Dyrlund TF. Gupta V. et al. Spider genomes provide insight into composition and evolution of venom and silk. Nat. Commun. 2014;5.

8. Schwager EE. Gendreau KL. Stanke M. Richards S. Garb JE. Haney RA. et al. House spider genome uncovers evolutionary shifts in the diversity and expression of black widow venom proteins associated with extreme toxicity. BMC Genomics. 2017;18.

9. Di Z. Xie C. Duan Z. Li Z. Liang S. Xu X. et al. Proteomic analysis of the venom from the scorpion *Mesobuthus martensii*. J. Proteomics. 2014;106:162–80.

10. Moran Y. Weinberger H. Sullivan JC. Reitzel AM. Finnerty JR. Gurevitz M. Concerted evolution of sea anemone neurotoxin genes is revealed through analysis of the *Nematostella vectensis* genome. Mol. Biol. Evol. 2008;25:737–47.

11. Moran Y. Weinberger H. Reitzel AM. Sullivan JC. Kahn R. Gordon D. et al. Intron Retention as a Posttranscriptional Regulatory Mechanism of Neurotoxin Expression at Early Life Stages of the Starlet Anemone *Nematostella vectensis*. J. Mol. Biol. 2008;380:437–43.

12. Moran Y. Columbus-Shenkar YY. Macrander J. Modepalli V. Reitzel AM. Fridrich A. et al. Dynamics of venom composition across a complex life cycle. Elife [Internet]. 2018;7. Available from: http://dx.doi.org/10.1101/159889

13. Brekhman V. Lotan T. Rachamim T. Morgenstern D. Aharonovich D. Sher D. The Dynamically Evolving Nematocyst Content of an Anthozoan, a Scyphozoan, and a Hydrozoan. Mol. Biol. Evol. 2014;32:740–53.

14. Sher D. Fishman Y. Zhang M. Lebendiker M. Gaathon A. Mancheño JM. et al. Hydralysins, a new category of β-pore-forming toxins in cnidaria. J. Biol. Chem. 2005;280:22847–55.

15. Vonk FJ. Casewell NR. Henkel C V. Heimberg AM. Jansen HJ. McCleary RJR. et al. The king cobra genome reveals dynamic gene evolution and adaptation in the snake venom system. Proc. Natl. Acad. Sci. [Internet]. Proceedings of the National Academy of Sciences; 2013;110:20651–20656. Available from: http://dx.doi.org/10.1073/pnas.1314702110

16. Schield DR. Card DC. Hales NR. Perry BW. Pasquesi GM. Blackmon H. et al. The origins and evolution of chromosomes, dosage compensation, and mechanisms underlying venom regulation in snakes. Genome Res. [Internet]. 2019; Available from: http://genome.cshlp.org/lookup/doi/10.1101/gr.240952.118

17. Mofiz E. Papenfuss AT. Temple-Smith P. Wong ESW. King GF. Whittington CM. et al. Proteomics and Deep Sequencing Comparison of Seasonally Active Venom Glands in the Platypus Reveals Novel Venom Peptides and Distinct Expression Profiles. Mol. Cell. Proteomics. 2012;11:1354–64.
