# Supplementary material for: Toxins from scratch? Diverse, multimodal gene origins in the predatory robber fly Dasypogon diadema indicate a dynamic venom evolution in dipteran insects
Source: Gigascience. 2019 Jul 9;8(7):giz081. doi: 10.1093/gigascience/giz081 (PMC6615979; doi:10.1093/gigascience/giz081)
Supplement: giz081_Response_to_Reviewer_Comments_Original_Submission [file giz081_response_to_reviewer_comments_original_submission.pdf]

Author's Response To Reviewer Comments

Close

GIGA-D-19-00072
Toxins from scratch? - Diverse, multimodal gene origins in predatory robber flies indicate dynamic venom evolution in dipteran insects
Stephan Holger Drukewitz; Eivind Andreas Baste Undheim; Lukas Bokelmann; Bjoern Marcus von Reumont
GigaScience

Dear Dr. von Reumont,
Your manuscript "Toxins from scratch? - Diverse, multimodal gene origins in predatory robber flies indicate dynamic venom evolution in dipteran insects" (GIGA-D-19-00072) has been assessed by our reviewers. Although it is of interest, we are unable to consider it for publication in its current form. The reviewers have raised a number of points which we believe would improve the manuscript and may allow a revised version to be published in GigaScience.

Reviewer #2 has provided potential solutions to their major concern regarding the transcriptome assembly which, I believe, if followed correctly, can further improve the transcriptome assembly and if not, at least provide better clarification with regards to the false positives.

Their reports, together with any other comments, are below. Please also take a moment to check our website at https://www.editorialmanager.com/giga/ for any additional comments that were saved as attachments.

In addition, please register any new software application in the SciCrunch.org database to receive a RRID (Research Resource Identification Initiative ID) number, and include this in your manuscript. This will facilitate tracking, reproducibility and re-use of your tool.

If you are able to fully address these points, we would encourage you to submit a revised manuscript to GigaScience. Once you have made the necessary corrections, please submit online at:
https://www.editorialmanager.com/giga/

If you have forgotten your username or password please use the "Send Login Details" link to get your login information. For security reasons, your password will be reset.

Please include a point-by-point within the 'Response to Reviewers' box in the submission system. Please ensure you describe additional experiments that were carried out and include a detailed rebuttal of any criticisms or requested revisions that you disagreed with. Please also ensure that your revised manuscript conforms to the journal style, which can be found in the Instructions for Authors on the journal homepage.

The due date for submitting the revised version of your article is 11 Jul 2019. I look forward to receiving your revised manuscript soon.

Best wishes,
Nicole Nogoy, Ph.D

Reviewer reports:
• Reviewer #1:
Authors have adequately addressed my minor comments to the original version of their manuscript (and as far as I can understand also to the other reviewers comments and criticism). I'm thus happy to recommend publication of this revised paper.

Reply
Thank you

• Reviewer #2:
This work was collaboratively reviewed by myself (Mark Margres), Mark DiMeo, Erich Hofmann, Andrew Mason, Rhett Rautsaw, Erin Stiers, and Jason Strickland.
This manuscript is a resubmission of a manuscript we previously reviewed. Here, the authors report the findings of a comparative genomics study examining the molecular mechanisms of toxin evolution in robber flies. The new version of the manuscript is much improved in readability, which makes it much easier to follow the methodological approaches taken and parse their main findings (with a few exceptions). For example, the new detail regarding the genome assembly in the methods and Table 2 has alleviated our major concern regarding the quality of the genome. We still, however, have a few major and minor comments that need to be dealt with before we can recommend acceptance. We have also outlined potential solutions to our major concerns below.

Major comments:
1. We still find the authors sole use of Trinity for transcriptome assembly (with default settings) to be a major flaw in the manuscript. We discuss this in several parts below:

a. The authors state in their response to our previous comment "This is due to the large number of false positives arising from the ancient homology of toxins to physiological proteins and the rampant convergent evolution of toxin in independent venomous lineages …We rely for that reason on the proteomic analyses of venom as the reference-base-line to annotate our venom gland transcriptomes and the genome. As a consequence, the issue the reviewers find in our assembly exclusively based on Trinity is not an issue."
Because the authors rely on proteomic analyses to confirm the secretion of toxins (an approach we appreciate and are glad the authors took), the false positive issue is not relevant. In other words, de novo assembled false positives do not matter when you are using the proteome to confirm secretion and the reference genome to estimate transcription. Therefore, false positives are not a valid reason to not include other assemblers because the authors' approach adequately eliminates these transcripts prior to genome annotation and toxin-specific analyses.
We are most concerned with false negatives; the authors cannot annotate in the genome what they did not assemble in the transcriptome, nor can they proteomically-confirm toxins that were not assembled. For example, the authors state in the manuscript on lines 448-450: "For protein identification, MS/MS spectra were searched against sequence lists consisting of both the translated venom gland and body transcriptomes of D. diadema using ProteinPilot v5.0." Again on lines 523-525: "The annotated protein-coding genes of D. diadema were matched with the venom gland proteins identified via proteomics applying a strict threshold." As a result, toxins that were not assembled by Trinity cannot be identified in the proteome nor the genome, potentially biasing the authors' results in a substantial way.
Recent work has shown that different transcriptome assemblers have different biases when it comes to de novo assembling particular types of toxins [Holding et al. (2018). Evaluating the performance of de novo assembly methods for venom-gland transcriptomics. Toxins, 10(6), 249], and using a single assembler almost certainly guarantees an incomplete venom-gland transcriptome. As we stated in the previous review, missing toxins will directly affect the authors' main conclusions regarding the number of single-copy versus multi-copy toxins and, therefore, the modality of venom evolution, and this weakness cannot be ignored.

a.) Reply
In general, false negatives are difficult to discuss because many aspects influence that venom proteins are not recovered. In our study we discuss and present a trend of the top 30 venom protein candidates for which we find indications of orphan genes and indications for single and multi-copy genes in our data. We are aware that there is the possibility that we miss false negatives, which might have to be added to our recovered venom proteins. From our perspective the reviewers do not acknowledge that it remains unknown to what extent their experience with snakes and scorpions can be transferred to rather unknown venomous species and novel venom proteins. The study in Toxins (Holding etal. 2018) they refer to when addressing the benefit of multiple assemblers (some of the reviewers are co-authors on this study) compares the results of different assemblers for well-known venom proteins of snakes and scorpions. The results are further assessed exclusively with the number of toxin transcripts that are recovered, which could be questioned as well because the assumption “the higher the better” (more toxin transcripts = better assembly) is difficult and possibly misleading without genome data.

Nevertheless, we take the critics very serious and tested the impact of different assemblers on our results for the top 30 candidates by comparing Trinity vs RNASpades and TransAbyss. The results, which

we include now in the manuscript's discussion and supplementary information support our conclusions and show that our story on the top 30 candidates holds up, most of them are recovered by all assemblers.

We hope therefore, that the reviewer group and the editorial board support our choice to follow the second possibility (suggested also by the reviewers) to not re-analyze all data from scratch, but instead to address their critics and concerns regarding our analyses now more extended in our discussion. We state also - as demanded – more detailed the possible impact of using only Trinity as transcriptome assembler. A critical paragraph regarding the impact of multiple assemblers is included in this section and refers the reader to the supplement where more detailed results of the assembly comparison are shown. This includes a new supplementary table (Supp Tab 8 and Supp Tab. 9) in which we compare the assembly results for the top 30 toxins between Trinity, RNASpades and Transabyss based on the Transdecoder-translated protein CDS.

---

b. The authors state in their response "our assumption is (due to gene set completeness score from Busco)." BUSCO is not a good representation of completeness for toxin annotation. BUSCO loci are extremely conserved and valuable for assessing overall genome assembly quality, but certainly not for toxins. Toxins are typically part of large multi-gene complexes which evolve and duplicate rapidly (as this and other studies have shown) and, as a result, are much more difficult to assemble. A high completeness based on BUSCO does not reflect the completeness of toxin annotation (which in our experience needs to be done manually), so it is very possible that the authors have not accurately assembled the toxin regions and still receive a high completeness based on BUSCO. If manual annotation/verification of toxin genes has been performed, this detail must be added to the manuscript. If not, we strongly recommend the authors doing so.

b.) Reply
We argue based on the high BUSCO value that our Genome is of better quality than stated by the reviewer in their first review. Busco is a general indication of the gene completeness using a reference gene set, which is specific for a lineage. The reviewers are right, that the used gene sets need to be somehow conserved, the level of conservation depends on the used gene set. In our case we used the core set of holometabolous insects, which seemed reasonable because of the analyses of Lepidopteran and Dipteran genomes.
We agree with the reviewers that a high Busco score is no guarantee for the accurate annotation or assembly of all genes. However, in our opinion it seems counterintuitive to conclude that even with a high Busco score the majority of toxin genes is misassembled or misannotated.
We prefer to not manually annotate venom protein genes on the robber fly genome in this manuscript, because most of the proteins we describe are novel. We agree, that manual annotation is sometimes more precise and can improve gene annotation compared to automatized algorithms, however, a prerequisite to ensure this is the knowledge about the gene structure of certain gene families. A manual annotation of novel and unknown venom proteins from a novel organism like in our case might be rather arbitrary and hamper the reproducibility of the analysis.

c. The authors state in their response "If the sequence similarity between such toxin variants is so high that only one is recovered in the Trinity assembly, this assembled transcript can be utilized by Maker to annotate both variants on the genome (the one covered in the initial Trinity assembly and the one that was missed in this assembly)." Trinity most likely is missing more than just paralogs of already identified genes, and as we previously stated in our earlier review missing toxins would affect their downstream analyses and potentially the main conclusion regarding the multi-modal fashion of robber fly venom evolution.

We see two ways to address this flaw. First (and the most thorough and best course of action), the authors would incorporate other de novo assemblers into their workflow (e.g., VTBuilder, BinPacker, SOAPdenovo, Oases) and ensure that (1) their toxin identification from the venom-gland transcriptome is complete and (2) their inferences regarding the frequencies of different modalities (single vs multi-copy genes) are not erroneous. Yes, this would then require the authors to re-conduct all analyses, including genome annotation, which is why we made this a major point to address in our previous review. We still find this to be, by far, the best course of action. If, however, the editor wishes to pursue a timelier route, at minimum all of the points above would need to be thoroughly addressed in the Discussion. The authors must state how using a single assembler could lead to unassembled, missing toxins and, therefore, directly affect their main conclusions regarding the different modes of toxin

evolution.

c.) Reply
See our response in a). We include now a critical discussion and included an assembly comparison for the top 30 toxin candidates, which shows that most candidates are covered in all assemblies. In this section we cite now as well the study the reveiwers refer to, Holding et al. 2018 (Toxins).
---

2. The naming convention is still unclear in the manuscript. The authors cite the work of Undheim et al. (2014) but appear to use a reduced form of this convention throughout the manuscript (excluding line 312 which appears to be the only correct usage of the naming convention). The result is that the manuscript reports several toxin variants that are all listed as the same toxin (e.g. Asilidin2 of which there are ten copies). Using this shortened taxonomy (that names toxins identically) is uninformative. For instance, if a reader wished to investigate a particularly highly expressed Asilidin2 variant, how would they distinguish which was the most highly expressed without having a corresponding table of TPM values? It would seem that a small amount of effort to differentiate the toxin variations would provide a great deal of information. Figure 4 also illustrates how the lack of naming specificity is a limitation. Here, there are nine variants all labelled Asilidin2, and all variants pass through the same node, suggesting that all variants are identical (though the authors imply they are not).

The solution here is simple: uniquely name each toxin based on the conventions cited in the response and the manuscript. From Undheim et al. (2014) "Using the proposed nomenclature, the recently described voltage-gated potassium channel (KV) inhibitor k-SLPTX-Ssm1a would thus become k-SLPTX3-Ssm1a, indicating that it is a KV inhibiting member of Scolopendromorpha toxin family number three that was first discovered in S. subspinipes mutilans (Yang et al. 2012). As an example, using this system, one is able to convey that m-SLPTX15-Ssd1a (GenBank: KC144793) and k-SLPTX15-Ssd1a (GenBank: KC144556) are both members of the same toxin family, whereas k-SLPTX3-Ssm1a is not but at the same time provide information on pharmacological activity and organismal origin of these toxins." This nomenclature is quite different from Asilidin2 representing ten different variants. Given that convention, each toxin name should have three parts, and the full name should be included throughout the manuscript, including Figure 4 and the supplemental.

2.) Reply
That is a good point, we agree with the reviewers. Our intention was to keep it easier and better to read, but renaming the sequences in the suggested way indeed gives all details that allow the reader to differentiate clearly. We changed this and adapted everything accordingly in the whole manuscript, including the supplementary material (Supp Tab 6). In the additional material stored in the GigaSciecne cloud we show now for each novel protein the corresponding networks that were used to identify the clades of each transcript and their phylogenetic position.

Minor comments:

Line 42: should be rewritten as "While homologs were identified for 15 out of 30 predominant venom proteins, the remaining 15 highly expressed venom proteins…"
Reply: Has been re-written

Line 48: "yet" should be replaced with "been"
Reply: replaced

Line 57: Remove "a" before "gene duplication"
Reply: removed

Line 58: Remove "a" before "neo"
Reply: removed

Line 59: Change "which results" to "resulting"
Reply: Has been changed

Line 63: Move comma after "(Supp. Tab. 1)" to after "and". Also, the newly published Crotalus viridis genome (Schield et al. (2019). The origins and evolution of chromosomes, dosage compensation, and mechanisms underlying venom regulation in snakes. Genome research) can be added to Supp. Table 1.

Reply: Comma has been moved. The recent publication of Schield et al. (2019) has been added to the Supp. Table 1.

Line 69 - Add a comma after "toxins"
Reply: Comma has been added

Line 78: Capitalize "King Cobra"
Reply: Despite king cobra and other species names are often falsely capitalized in manuscript titles, this is not correct to our knowledge. King cobra is a common name and should not be capitalized (referring here to sources such as oxford dictionary and a professional editor). Only names of species that contain a proper noun, like Przewalski's horse are capitalized.

Lines 91, 296, 397, 778, 799: Replace "genders" with "sexes"
Reply: Replaced.

Line 106-107: "12 species of fly (dipteran) and butterfly (lepidopteran) model organisms, 10 of which were dipterans" should be "10 dipteran and two butterfly (lepidopteran) species."
Reply: This sentence is rephrased.

Line 114: "currently largest data set of comparative genomics" should be "largest comparative genomics data set to date"
Reply: Re-written

Lines 115-116: "the promises of, but also the need for," should be "the potential and necessity of"
Reply: rephrased

Line 141: Remove "up"
Reply: removed

Lines 180, 432, 517: These should be numeric citations to be consistent with the main body of the manuscript.
Reply: to do

Line 181: "iin" should be "in"
Reply: Corrected

Line 190: "created" should be "recovered"
Reply: Corrected

Line 215: "predicted if" should be "tested whether"
Reply: Changed

Line 246-246: "The 27 putative toxin transcripts that are assigned to orthogroups distribute into 20 different orthogroups" should be "The 27 putative toxin transcripts were distributed among 20 different orthogroups."
Reply: Corrected

Line 253: Should "comprise" be "contain"?
Reply: We replaced comprise by contain, it suits slightly better.

Line 291: Remove parenthetical note
Reply: Removed

Line 300: comma after "In general"
Reply: Comma set.

Lines 313-315: "Facilitating a fast and efficient paralysis of prey asilidin1 probably embody a biologically important venom component in robber fly venom" 1 needs to be subscript and grammatical mistakes (e.g., embody is incorrect).
Reply: Corrected

Line 337: "…evolution without gene duplication These genes…" Missing period between sentences.

Reply: Corrected

Line 410: Add comma after "sexes"
Reply: Added

Line 416: Change “data is” to “data are”
Reply: Changed

Line 419: Please clarify “Own and published”
Reply: Done

Line 431: “water prepared” should be “water and prepared”
Reply: Corrected

Line 471: Add the number of MiSeq runs used to generate the data.
Reply: We like to point out that the number of runs is in many cases not really informative. Machines are often used with shared samples in sequencing facilities, which was as well the case for our library samples and sequencing. To avoid a misleading of the reader we refrain to mention numbers of sequence runs. We state very clear the generated output, which is the only precise “unit” to assess and evaluate our sequencing effort and depth.

Line 480: Please include parameter settings for MaSuRCA. If default, say “default settings”.
Reply: Done

Line 519: add "our" between "against" and "own"
Reply: Added

Line 545: "deselected" should be "excluded"
Reply: Changed

Line 546-547: "As outgroup taxa the lepidopterans Bombyx mori and Danaus plexippus were chosen" should be "The lepidopterans Bombyx mori and Danaus plexippus were chosen as outgroup taxa."
Reply: Corrected

Figure 1: Please move the male and female symbols from the edge of the figure.
Reply: We consider this point as rather personal preference related, but follow the reviewers and move the symbols more in the middle.

Figure 4 requires the reader to also reference Figure 3 for the node numbers to have meaning. We suggest the authors include a small mockup of the phylogeny with labeled nodes or combine Figure 4 with Figure 3 (although the latter strategy makes for a very complex figure). We also think the figure could be improved by differentiating the different "multi copy" sections with an informative label in the figure itself rather than through asterisks defined in the caption.
Reply: We changed the figure slightly by adding the node numbers more clearly which correspond to figure 3 (Adding a phylogeny mockup was too confusing, we tried that actually).

Supplemental Figure 4 - Resolution is not good enough to see gene names.
Reply: Done

Close
