# Supplementary material for: Toxins from scratch? Diverse, multimodal gene origins in the predatory robber fly Dasypogon diadema indicate a dynamic venom evolution in dipteran insects
Source: Gigascience. 2019 Jul 9;8(7):giz081. doi: 10.1093/gigascience/giz081 (PMC6615979; doi:10.1093/gigascience/giz081)
Supplement: giz081_Response_to_Reviewer_Comments_Revision_1 [file giz081_response_to_reviewer_comments_revision_1.pdf]

Author's Response To Reviewer Comments

Close

Letter to the editor:

Dear Nicole Nogoy and dear Laurie Goodman,

We would like to submit our second revision on our manuscript:

Toxins from scratch? - Diverse, multimodal gene origins in predatory robber flies indicate dynamic venom evolution in dipteran insects

We really appreciate that in this review round Laurie Goodman stepped in and provided some editing of our manuscript. We fully accept the editorial changes and phrasing, and thank for the effort to bring the issue of false negatives to a final point.
We only changed one phrasing in the discussion part provided by Laurie Goodman, instead of missed false negatives that might replace our top 30 candidates, we used the phrasing might be added to our top 30 candidates. Otherwise we fully agree with the wording and went once more over the manuscript and corrected few typos that were overlooked.

The change, that the reviewers demanded was that we should minimally take out the sentences about validity of our results, which is no longer in this latest manuscript version. We thank again for the effort of the editors and reviewers and hope that our manuscript is now in its final stage.

Thank you very much for your combined efforts and best regards on behalf of all authors,
bjoern v reumont

Response to reviewers

We see the major points and after some sugggestions of the editor-in-chief the discussion was changed accordingly to the open or not addressed demands of the reviewers.
The discussion and methods parts were now re-writen to address finally more clearly the false negatives and to state more explicitley that any false negatives that are not covered may impact on our results.

The sentence about validity is removed as demanded by the reviewers.

Best regards on behalf of all co-authors,
bjoern v reumont

Close
