# Supplementary material for: Toxins from scratch? Diverse, multimodal gene origins in the predatory robber fly Dasypogon diadema indicate a dynamic venom evolution in dipteran insects
Source: Gigascience. 2019 Jul 9;8(7):giz081. doi: 10.1093/gigascience/giz081 (PMC6615979; doi:10.1093/gigascience/giz081)
Supplement: giz081_Reviewer_2_Report_Original_Submission — Mark Margres -- 4/10/2019 Reviewed [file giz081_reviewer_2_report_original_submission.pdf]

Reviewer Report

Title: Toxins from scratch? - Diverse, multimodal gene origins in the predatory robber fly Dasypogon diadema indicate a dynamic venom evolution in dipteran insects

Version: Original Submission **Date:** 4/10/2019

Reviewer name: Mark Margres

Reviewer Comments to Author:

This work was collaboratively reviewed by myself (Mark Margres), Mark DiMeo, Erich Hofmann, Andrew Mason, Rhett Rautsaw, Erin Stiers, and Jason Strickland.

This manuscript is a resubmission of a manuscript we previously reviewed. Here, the authors report the findings of a comparative genomics study examining the molecular mechanisms of toxin evolution in robber flies. The new version of the manuscript is much improved in readability, which makes it much easier to follow the methodological approaches taken and parse their main findings (with a few exceptions). For example, the new detail regarding the genome assembly in the methods and Table 2 has alleviated our major concern regarding the quality of the genome. We still, however, have a few major and minor comments that need to be dealt with before we can recommend acceptance. We have also outlined potential solutions to our major concerns below.

Major comments:

1. We still find the authors sole use of Trinity for transcriptome assembly (with default settings) to be a major flaw in the manuscript. We discuss this in several parts below:

a. The authors state in their response to our previous comment "This is due to the large number of false positives arising from the ancient homology of toxins to physiological proteins and the rampant convergent evolution of toxin in independent venomous lineages …We rely for that reason on the proteomic analyses of venom as the reference-base-line to annotate our venom gland transcriptomes and the genome. As a consequence, the issue the reviewers find in our assembly exclusively based on Trinity is not an issue." Because the authors rely on proteomic analyses to confirm the secretion of toxins (an approach we appreciate and are glad the authors took), the false positive issue is not relevant. In other words, de novo assembled false positives do not matter when you are using the proteome to confirm secretion and the reference genome to estimate transcription. Therefore, false positives are not a valid reason to not include other assemblers because the authors' approach adequately eliminates these transcripts prior to genome annotation and toxin-specific analyses. We are most concerned with false negatives; the authors cannot annotate in the genome what they did not assemble in the transcriptome, nor can they proteomically-confirm toxins that were not assembled. For example, the authors state in the manuscript on lines 448-450: "For protein identification, MS/MS spectra were searched against sequence lists consisting of both the translated venom gland and body transcriptomes of D. diadema using ProteinPilot v5.0." Again on lines 523-525: "The annotated protein-coding genes of D. diadema were matched with the venom gland proteins identified via proteomics applying a strict threshold." As a result, toxins that were not assembled by Trinity cannot be identified in the proteome nor the genome, potentially biasing the authors' results in a substantial way. Recent work has shown

that different transcriptome assemblers have different biases when it comes to de novo assembling particular types of toxins [Holding et al. (2018). Evaluating the performance of de novo assembly methods for venom-gland transcriptomics. Toxins, 10(6), 249], and using a single assembler almost certainly guarantees an incomplete venom-gland transcriptome. As we stated in the previous review, missing toxins will directly affect the authors' main conclusions regarding the number of single-copy versus multi-copy toxins and, therefore, the modality of venom evolution, and this weakness cannot be ignored.

b. The authors state in their response "our assumption is (due to gene set completeness score from Busco)." BUSCO is not a good representation of completeness for toxin annotation. BUSCO loci are extremely conserved and valuable for assessing overall genome assembly quality, but certainly not for toxins. Toxins are typically part of large multi-gene complexes which evolve and duplicate rapidly (as this and other studies have shown) and, as a result, are much more difficult to assemble. A high completeness based on BUSCO does not reflect the completeness of toxin annotation (which in our experience needs to be done manually), so it is very possible that the authors have not accurately assembled the toxin regions and still receive a high completeness based on BUSCO. If manual annotation/verification of toxin genes has been performed, this detail must be added to the manuscript. If not, we strongly recommend the authors doing so.

c. The authors state in their response "If the sequence similarity between such toxin variants is so high that only one is recovered in the Trinity assembly, this assembled transcript can be utilized by Maker to annotate both variants on the genome (the one covered in the initial Trinity assembly and the one that was missed in this assembly)." Trinity most likely is missing more than just paralogs of already identified genes, and as we previously stated in our earlier review missing toxins would affect their downstream analyses and potentially the main conclusion regarding the multi-modal fashion of robber fly venom evolution.

We see two ways to address this flaw. First (and the most thorough and best course of action), the authors would incorporate other de novo assemblers into their workflow (e.g., VTBuilder, BinPacker, SOAPdenovo, Oases) and ensure that (1) their toxin identification from the venom-gland transcriptome is complete and (2) their inferences regarding the frequencies of different modalities (single vs multi-copy genes) are not erroneous. Yes, this would then require the authors to re-conduct all analyses, including genome annotation, which is why we made this a major point to address in our previous review. We still find this to be, by far, the best course of action. If, however, the editor wishes to pursue a timelier route, at minimum all of the points above would need to be thoroughly addressed in the Discussion. The authors must state how using a single assembler could lead to unassembled, missing toxins and, therefore, directly affect their main conclusions regarding the different modes of toxin evolution.

2. The naming convention is still unclear in the manuscript. The authors cite the work of Undheim et al. (2014) but appear to use a reduced form of this convention throughout the manuscript (excluding line 312 which appears to be the only correct usage of the naming convention). The result is that the manuscript reports several toxin variants that are all listed as the same toxin (e.g. Asilidin2 of which there are ten copies). Using this shortened taxonomy (that names toxins identically) is uninformative. For instance, if a reader wished to investigate a particularly highly expressed Asilidin2 variant, how would they distinguish which was the most highly expressed without having a corresponding table of

TPM values? It would seem that a small amount of effort to differentiate the toxin variations would provide a great deal of information. Figure 4 also illustrates how the lack of naming specificity is a limitation. Here, there are nine variants all labelled Asilidin2, and all variants pass through the same node, suggesting that all variants are identical (though the authors imply they are not).

The solution here is simple: uniquely name each toxin based on the conventions cited in the response and the manuscript. From Undheim et al. (2014) "Using the proposed nomenclature, the recently described voltage-gated potassium channel (KV) inhibitor k-SLPTX-Ssm1a would thus become k-SLPTX3-Ssm1a, indicating that it is a KV inhibiting member of Scolopendromorpha toxin family number three that was first discovered in S. subspinipes mutilans (Yang et al. 2012). As an example, using this system, one is able to convey that m-SLPTX15-Ssd1a (GenBank: KC144793) and k-SLPTX15-Ssd1a (GenBank: KC144556) are both members of the same toxin family, whereas k-SLPTX3-Ssm1a is not but at the same time provide information on pharmacological activity and organismal origin of these toxins." This nomenclature is quite different from Asilidin2 representing ten different variants. Given that convention, each toxin name should have three parts, and the full name should be included throughout the manuscript, including Figure 4 and the supplemental.

Minor comments:

Line 42: should be rewritten as "While homologs were identified for 15 out of 30 predominant venom proteins, the remaining 15 highly expressed venom proteins…"

Line 48: "yet" should be replaced with "been"

Line 57: Remove "a" before "gene duplication"

Line 58: Remove "a" before "neo"

Line 59: Change "which results" to "resulting"

Line 63: Move comma after "(Supp. Tab. 1)" to after "and". Also, the newly published Crotalus viridis genome (Schield et al. (2019). The origins and evolution of chromosomes, dosage compensation, and mechanisms underlying venom regulation in snakes. Genome research) can be added to Supp. Table 1.

Line 69 - Add a comma after "toxins"

Line 78: Capitalize "King Cobra"

Lines 91, 296, 397, 778, 799: Replace "genders" with "sexes"

Line 106-107: "12 species of fly (dipteran) and butterfly (lepidopteran) model organisms, 10 of which were dipterans" should be "10 dipteran and two butterfly (lepidopteran) species."

Line 114: "currently largest data set of comparative genomics" should be "largest comparative genomics data set to date"

Lines 115-116: "the promises of, but also the need for," should be "the potential and necessity of"

Line 141: Remove "up"

Lines 180, 432, 517: These should be numeric citations to be consistent with the main body of the manuscript.

Line 181: "iin" should be "in"

Line 190: "created" should be "recovered"

Line 215: "predicted if" should be "tested whether"

Line 246-246: "The 27 putative toxin transcripts that are assigned to orthogroups distribute into 20 different orthogroups" should be "The 27 putative toxin transcripts were distributed among 20 different orthogroups."

Line 253: Should "comprise" be "contain"?
Line 291: Remove parenthetical note
Line 300: comma after "In general"
Lines 313-315: "Facilitating a fast and efficient paralysis of prey asilidin1 probably embody a biologically important venom component in robber fly venom" 1 needs to be subscript and grammatical mistakes (e.g., embody is incorrect).
Line 337: "…evolution without gene duplication These genes…" Missing period between sentences.
Line 410: Add comma after "sexes"
Line 416: Change "data is" to "data are"
Line 419: Please clarify "Own and published"
Line 431: "water prepared" should be "water and prepared"
Line 471: Add the number of MiSeq runs used to generate the data.
Line 480: Please include parameter settings for MaSuRCA. If default, say "default settings".
Line 519: add "our" between "against" and "own"
Line 545: "deselected" should be "excluded"
Line 546-547: "As outgroup taxa the lepidopterans Bombyx mori and Danaus plexippus were chosen" should be "The lepidopterans Bombyx mori and Danaus plexippus were chosen as outgroup taxa."
Figure 1: Please move the male and female symbols from the edge of the figure.
Figure 4 requires the reader to also reference Figure 3 for the node numbers to have meaning. We suggest the authors include a small mockup of the phylogeny with labelled nodes or combine Figure 4 with Figure 3 (although the latter strategy makes for a very complex figure). We also think the figure could be improved by differentiating the different "multi copy" sections with an informative label in the figure itself rather than through asterisks defined in the caption.
Supplemental Figure 4 - Resolution is not good enough to see gene names.

Methods

Are the methods appropriate to the aims of the study, are they well described, and are necessary controls included? Choose an item.

Conclusions

Are the conclusions adequately supported by the data shown? Choose an item.

Reporting Standards

Does the manuscript adhere to the journal's guidelines on [minimum standards of reporting?](#) Choose an item.

Choose an item.

Statistics

Are you able to assess all statistics in the manuscript, including the appropriateness of statistical tests used? Choose an item.

Quality of Written English

Please indicate the quality of language in the manuscript: Choose an item.

Declaration of Competing Interests

Please complete a declaration of competing interests, considering the following questions:

- Have you in the past five years received reimbursements, fees, funding, or salary from an organisation that may in any way gain or lose financially from the publication of this manuscript, either now or in the future?
- Do you hold any stocks or shares in an organisation that may in any way gain or lose financially from the publication of this manuscript, either now or in the future?
- Do you hold or are you currently applying for any patents relating to the content of the manuscript?
- Have you received reimbursements, fees, funding, or salary from an organization that holds or has applied for patents relating to the content of the manuscript?
- Do you have any other financial competing interests?
- Do you have any non-financial competing interests in relation to this paper?

If you can answer no to all of the above, write 'I declare that I have no competing interests' below. If your reply is yes to any, please give details below.

I declare that I have no competing interests.

I agree to the open peer review policy of the journal. I understand that my name will be included on my report to the authors and, if the manuscript is accepted for publication, my named report including any attachments I upload will be posted on the website along with the authors' responses. I agree for my report to be made available under an Open Access Creative Commons CC-BY license (http://creativecommons.org/licenses/by/4.0/). I understand that any comments which I do not wish to be included in my named report can be included as confidential comments to the editors, which will not be published.

I agree to the open peer review policy of the journal

To further support our reviewers, we have joined with Publons, where you can gain additional credit to further highlight your hard work (see: https://publons.com/journal/530/gigascience). On publication of this paper, your review will be automatically added to Publons, you can then choose whether or not to claim your Publons credit. I understand this statement.

Yes Choose an item.
