# Supplementary material for: Toxins from scratch? Diverse, multimodal gene origins in the predatory robber fly Dasypogon diadema indicate a dynamic venom evolution in dipteran insects
Source: Gigascience. 2019 Jul 9;8(7):giz081. doi: 10.1093/gigascience/giz081 (PMC6615979; doi:10.1093/gigascience/giz081)
Supplement: giz081_Reviewer_2_Report_Revision_1 — Mark Margres -- 5/16/2019 Reviewed [file giz081_reviewer_2_report_revision_1.pdf]

Reviewer Report

Title: Toxins from scratch? - Diverse, multimodal gene origins in the predatory robber fly Dasypogon diadema indicate a dynamic venom evolution in dipteran insects

Version: Revision 1 **Date:** 5/16/2019

Reviewer name: Mark Margres

Reviewer Comments to Author:

The authors have made some revisions based on our previous review and recommended solutions. Unfortunately, the authors have misinterpreted our main concern regarding false negatives. To reiterate, the authors cannot annotate in the genome what they did not assemble in the transcriptome, nor can they proteomically-confirm toxins that were not assembled. In our previous review, we suggested two possible solutions: (1) incorporate other de novo assemblers into their workflow (e.g., VTBuilder, BinPacker, SOAPdenovo, Oases) and ensure that their toxin identification from the venom-gland transcriptome is complete and their inferences regarding the frequencies of different modalities (single vs multi-copy genes) are not erroneous, or (2) address the weaknesses of their approach in the Discussion. The authors chose to take a hybrid approach and perform a few additional assemblies and discuss caveats in the Discussion. However, the authors have misunderstood our concerns. The authors "compared the performance of Trinity against two further transcriptome assemblers, RNASpades [31] and Transabyss [32], for our top 30 predominant toxins" (Lines 320-323). Our concern was not in the toxins that the authors did identify, but in the toxins the authors may NOT have identified. We trust that the top 30 candidates identified by the authors are real, but this approach does not at all address the issue of false negatives. The approach actually highlights our concern regarding using a single assembler; the authors state on lines 323-325 that not all 30 candidates were recovered by the additional assemblers, exemplifying how using one assembler ensures an incomplete transcriptome. Lines 320-329 should therefore be removed as this analysis certainly does not demonstrate that the authors conclusions are "valid" as stated on line 327.

Further, the authors state in their response "From our perspective the reviewers do not acknowledge that it remains unknown to what extent their experience with snakes and scorpions can be transferred to rather unknown venomous species and novel venom proteins. The study in Toxins (Holding etal. 2018) they refer to when addressing the benefit of multiple assemblers (some of the reviewers are co-authors on this study) compares the results of different assemblers for well-known venom proteins of snakes and scorpions. The results are further assessed exclusively with the number of toxin transcripts that are recovered, which could be questioned as well because the assumption "the higher the better" (more toxin transcripts = better assembly) is difficult and possibly misleading without genome data." From our perspective, the authors do not acknowledge that their own new results show that multiple assemblers ensure a more complete assembly in robber flies (and most likely across all venomous taxa): "Except for few candidates the majority of the top 30 candidate toxins were recovered with identical or highly identical sequence similarity in the additional assemblies." So yes, the conclusions of Holding et al. hold in robber flies as expected. All toxins share characteristics that make assembly difficult (e.g.,

high expression, redundancy of paralogs), and finding shared biases for multiple assemblers across venomous lineages separated by hundreds of millions of years (snakes, scorpions, and now robber flies) suggests that these are general trends for toxin loci. The authors are also incorrect in their assumption that the article evaluated assembly quality simply by the number of toxin transcripts identified. The cited article uses multiple metrics to evaluate the quality of toxin transcripts (coverage distributions, chimerism, etc.), but yes, more high-quality toxin transcripts does indicate a better assembly.
The authors also state "A critical paragraph regarding the impact of multiple assemblers is included in this section and refers the reader to the supplement where more detailed results of the assembly comparison are shown. This includes a new supplementary table (Supp Tab 8 and Supp Tab. 9) in which we compare the assembly results for the top 30 toxins between Trinity, RNASpades and Transabyss based on the Transdecoder-translated protein CDS."
As discussed above, this does not address our concern regarding false negatives. We trust that the 30 toxins they discuss are real as they were proteomically-validated. What about MS data that did not match a transcript? What did the authors do with these data?
Minimally the authors need to remove the statements regarding the "validity" of their results as stated above and all information regarding the assembly comparison for the 30 proteomically-validated toxins. We strongly encourage the authors to also look at the MS data that did not match an assembled transcript to see what toxins may have been missed.

Methods

Are the methods appropriate to the aims of the study, are they well described, and are necessary controls included? Choose an item.

Conclusions

Are the conclusions adequately supported by the data shown? Choose an item.

Reporting Standards

Does the manuscript adhere to the journal’s guidelines on minimum standards of reporting? Choose an item.

Choose an item.

Statistics

Are you able to assess all statistics in the manuscript, including the appropriateness of statistical tests used? Choose an item.

Quality of Written English

Please indicate the quality of language in the manuscript: Choose an item.

Declaration of Competing Interests

Please complete a declaration of competing interests, considering the following questions:

- Have you in the past five years received reimbursements, fees, funding, or salary from an organisation that may in any way gain or lose financially from the publication of this manuscript, either now or in the future?
- Do you hold any stocks or shares in an organisation that may in any way gain or lose financially from the publication of this manuscript, either now or in the future?
- Do you hold or are you currently applying for any patents relating to the content of the manuscript?
- Have you received reimbursements, fees, funding, or salary from an organization that holds or has applied for patents relating to the content of the manuscript?
- Do you have any other financial competing interests?
- Do you have any non-financial competing interests in relation to this paper?

If you can answer no to all of the above, write 'I declare that I have no competing interests' below. If your reply is yes to any, please give details below.

'I declare that I have no competing interests

I agree to the open peer review policy of the journal. I understand that my name will be included on my report to the authors and, if the manuscript is accepted for publication, my named report including any attachments I upload will be posted on the website along with the authors' responses. I agree for my report to be made available under an Open Access Creative Commons CC-BY license (http://creativecommons.org/licenses/by/4.0/). I understand that any comments which I do not wish to be included in my named report can be included as confidential comments to the editors, which will not be published.

I agree to the open peer review policy of the journal

To further support our reviewers, we have joined with Publons, where you can gain additional credit to further highlight your hard work (see: https://publons.com/journal/530/gigascience). On publication of this paper, your review will be automatically added to Publons, you can then choose whether or not to claim your Publons credit. I understand this statement.

Yes Choose an item.
